# Hybrid-Reinforced Phytocannabinoid-Functionalized PMMA Denture Base Composites: An In Vitro Study

**DOI:** 10.3390/polym18192361

**Published:** 2026-09-28

**Authors:** Aliye İpek Kuşçu, Yeliz Hayran, Ali Aydın

**Affiliations:** 1 Department of Prosthodontics, Faculty of Dentistry, Yozgat Bozok University, 66100 Yozgat, Türkiye; 2 Department of Prosthodontics, Faculty of Dentistry, Bursa Uludağ University, 16059 Bursa, Türkiye; 3 Department of Basic Medical Science, Faculty of Medicine, Yozgat Bozok University, 66100 Yozgat, Türkiye

**Keywords:** polymethyl methacrylate, hemp fiber, silk fibroin, phytocannabinoids, denture base resin, natural fiber composites, antimicrobial biomaterials, hybrid reinforcement, SDG 3: Ensure healthy lives and promote well-being for all at all ages

## Abstract

Polymethyl methacrylate (PMMA) remains the material of choice for denture base fabrication; however, its limited mechanical durability and susceptibility to microbial colonization continue to compromise long-term clinical performance. This study aimed to engineer a multifunctional PMMA denture base composite through hybrid reinforcement with hemp fiber (HF), silk fibroin (SF), and phytocannabinoid-rich fractions (Frac1–Frac4) and to comprehensively evaluate its mechanical, structural, antimicrobial, and cytocompatibility properties. Twenty experimental groups were fabricated, including conventional PMMA, PMMA/HF, PMMA/SF, and hybrid PMMA/HF/SF systems with or without 1 wt% phytocannabinoid fractions. Flexural strength, elastic modulus, Charpy impact strength, and Vickers hardness were determined according to standardized protocols. Structural characterization was performed using ATR-FTIR, X-ray diffraction (XRD), and scanning electron microscopy (SEM). Bacterial metabolic viability of *Streptococcus mutans* and *Lactobacillus acidophilus* was evaluated using the MTT assay, while cytotoxicity was assessed using the LDH assay. Mechanical outcomes were analyzed using two-way factorial ANOVA to evaluate the main effects of the material system and phytocannabinoid condition and their interaction; significant interactions were followed by Tukey-adjusted simple-effects comparisons. Biological data were analyzed using the procedures specified for those outcomes (α = 0.05). The material system and phytocannabinoid condition significantly affected all mechanical properties (all main-effect *p*-values ≤ 6.068 × 10^−83^). Significant material system × phytocannabinoid condition interactions were observed for flexural strength, Vickers hardness, and impact strength, whereas the interaction was not significant for elastic modulus. The PMMA/HF/SF control group exhibited the highest flexural strength (138.04 ± 2.05 MPa), elastic modulus (2.519 ± 0.017 GPa), Vickers hardness (26.14 ± 0.22 VHN), and impact strength (24.22 ± 0.22 kJ/m^2^). Although phytocannabinoid incorporation produced a gradual reduction in mechanical performance, all hybrid-reinforced formulations remained mechanically superior to unmodified PMMA. ATR-FTIR and XRD analyses confirmed preservation of the characteristic PMMA chemical structure and amorphous polymer architecture without evidence of new crystalline phases. SEM demonstrated markedly reduced bacterial adhesion and biofilm formation on phytocannabinoid-containing composites. The PMMA/HF/SF formulations exhibited the greatest antibacterial performance, reducing bacterial viability to 14.59–19.40% for *L. acidophilus* and 15.21–24.13% for *S. mutans*, while maintaining low cytotoxicity (<5%). These findings demonstrate the feasibility of combining hybrid natural-fiber reinforcement with phytocannabinoid functionalization in PMMA and support further investigation of this approach for denture base applications.

## 1. Introduction

Polymethyl methacrylate (PMMA) remains the most widely used denture base material in removable prosthodontics owing to its favorable esthetic appearance, ease of processing, satisfactory biocompatibility, low cost, and acceptable clinical performance [1]. Despite the emergence of high-performance polymers and digitally manufactured denture materials, conventional heat-polymerized PMMA continues to represent the clinical gold standard because of its balanced mechanical properties, ease of repair, and long-term clinical reliability [2]. Consequently, considerable research has focused on improving the functional performance of PMMA while preserving its advantageous processing characteristics and biocompatibility [3].

Nevertheless, the long-term clinical success of PMMA dentures remains limited by their relatively poor mechanical performance [4]. Midline fractures, fatigue failure, accidental dropping, insufficient impact resistance, and limited fracture toughness continue to represent the most common causes of denture failure [5]. Repeated masticatory loading may initiate microcrack formation within the polymer matrix, which subsequently propagates under cyclic stress and ultimately results in catastrophic fracture [6]. Therefore, improving the mechanical durability of PMMA without compromising its biological performance has become one of the major research priorities in contemporary prosthetic biomaterials.

Numerous reinforcement strategies have been proposed to overcome these mechanical limitations, including the incorporation of metallic fillers, ceramic particles, nanomaterials, and reinforcing fibers [7]. Among these approaches, fiber reinforcement has attracted particular attention because fibers are capable of transferring applied stresses from the polymer matrix, bridging propagating cracks, and improving energy absorption during fracture [8]. Previous studies have demonstrated that appropriate fiber reinforcement may significantly enhance flexural strength, impact resistance, and fracture toughness while maintaining the clinical applicability of PMMA denture base materials [9].

Natural fibers have recently emerged as attractive alternatives to synthetic reinforcing materials because of their low density, high specific strength, sustainability, biocompatibility, and environmental compatibility [10]. Hemp fiber is particularly promising owing to its excellent tensile strength, relatively high elastic modulus, and ability to improve stress transfer within polymeric matrices [11]. Likewise, silk fibroin has received increasing attention because of its outstanding biocompatibility, favorable interfacial adhesion, and capacity to enhance toughness through its protein-based molecular architecture [12]. The combination of these two natural fibers may therefore provide complementary reinforcing mechanisms capable of improving the structural performance of PMMA-based composites.

Beyond mechanical optimization, modern denture base materials are increasingly expected to exhibit additional biological functions capable of reducing microbial colonization while maintaining cytocompatibility [13]. In this context, phytocannabinoids have recently attracted considerable interest because of their antimicrobial, antioxidant, and anti-inflammatory activities. Their incorporation into dental biomaterials has been proposed as a promising strategy to improve biological performance without relying on conventional antimicrobial agents [14]. Nevertheless, introducing bioactive compounds into polymer matrices may also influence the mechanical behavior of the material, making comprehensive characterization essential before clinical application.

Although natural fiber reinforcement and phytocannabinoid functionalization have individually demonstrated promising outcomes, their simultaneous incorporation into PMMA has not been comprehensively investigated from a multifunctional biomaterials perspective [15]. In particular, little is known regarding whether hybrid reinforcement using hemp fiber and silk fibroin can preserve or enhance the mechanical integrity of phytocannabinoid-functionalized PMMA while maintaining its structural stability and biological performance [16]. Addressing this knowledge gap is essential for the rational design of next-generation denture base materials intended for long-term clinical use.

Therefore, the aim of the present study was to engineer and comprehensively characterize a multifunctional PMMA denture base composite reinforced with hemp fiber, silk fibroin, and phytocannabinoid fractions. The developed composites were evaluated in terms of flexural strength, elastic modulus, impact strength, surface hardness, structural characteristics, antimicrobial activity, and cytocompatibility. The null hypothesis was that the incorporation of natural fibers and phytocannabinoid fractions would not significantly influence the mechanical, structural, or biological properties of PMMA denture base materials.

## 2. Materials and Methods

### 2.1. Study Design

This in vitro experimental study was designed to investigate the structure–property relationship of multifunctional PMMA denture base composites reinforced with hemp fiber (HF), silk fibroin (SF), and phytocannabinoid-rich fractions. The experimental design was developed to determine whether hybrid natural fiber reinforcement could compensate for potential alterations in mechanical performance associated with phytochemical functionalization while simultaneously preserving the structural and biological characteristics of the material.

A total of four material systems were investigated: conventional heat-polymerized PMMA (Control), PMMA reinforced with silk fibroin (PMMA/SF), PMMA reinforced with hemp fiber (PMMA/HF), and PMMA reinforced with both hemp fiber and silk fibroin (PMMA/HF/SF). Each material system was further subdivided according to the incorporated phytocannabinoid fraction (Control, Frac1, Frac2, Frac3, and Frac4), resulting in twenty experimental groups. The selected material systems were designed to compare the performance of the unreinforced, single-fiber, and hybrid reinforcement formulations while simultaneously evaluating the influence of phytocannabinoid incorporation within each material system.

Sample size estimation was performed before the experimental study using G*Power software (version 3.1, Heinrich Heine University Düsseldorf, Düsseldorf, Germany). The original a priori calculation was based on a one-way fixed-effects ANOVA framework with 20 experimental groups, using α = 0.05, a desired power of 0.80, and a large effect size assumption (Cohen’s f = 0.40). This prespecified calculation supported the inclusion of 10 specimens per experimental group, yielding 200 specimens for each mechanical test. Because the final mechanical dataset has a balanced 4 × 5 factorial structure, the inferential analysis was subsequently performed using two-way factorial ANOVA to estimate the main effects of the material system and phytocannabinoid condition and their interaction. The original sample-size calculation is reported here as performed and was not retrospectively redefined as a factorial power analysis.

### 2.2. Materials

Commercial heat-polymerized polymethyl methacrylate (PMMA) denture base resin (Meliodent^®^, Kulzer GmbH, Hanau, Germany) was used as the polymer matrix throughout the study [17]. Hemp fiber was selected as the primary reinforcing phase owing to its high specific strength, favorable stiffness-to-weight ratio, and efficient stress-transfer capability, whereas silk fibroin was incorporated as a secondary reinforcing component to enhance interfacial adhesion, fracture toughness, and crack resistance.

Four phytocannabinoid-rich fractions (Frac1–Frac4), isolated and chemically characterized by our research group, were used as bioactive functional agents. These fractions were incorporated into the PMMA matrix to impart biological functionality while allowing simultaneous evaluation of their influence on the structural and mechanical performance of the developed composites.

The experimental formulation was designed according to a multifunctional reinforcement concept in which each component fulfilled a distinct role within the composite architecture. Hemp fiber served as the primary reinforcing phase responsible for improving load-bearing capacity, and silk fibroin functioned as an interfacial modifier that enhanced stress transfer and fracture resistance, whereas phytocannabinoid fractions acted exclusively as bioactive functional additives rather than mechanical reinforcing fillers. Consequently, the composite system enabled simultaneous assessment of mechanical reinforcement and biological functionalization within a single PMMA platform. The composition, source, and functional role of all materials used for fabrication of the multifunctional PMMA composites are summarized in Table 1.

### 2.3. Preparation of Phytocannabinoid Fractions

Hemp flowers were obtained from the Hemp Research Institute of Yozgat Bozok University (Yozgat, Türkiye). The dried flowers were ground into a fine powder using a laboratory grinder and subjected to decarboxylation at 105 °C for 1 h in a drying oven to convert cannabinoid acids into their neutral forms.

Following decarboxylation, phytocannabinoids were extracted using a supercritical CO_2_ extraction system under optimized conditions of 230 bar pressure, 50 °C temperature, and a CO_2_ flow rate of 50 kg h^−1^. The resulting crude extract was mixed with ethanol at a 1:1 (m/v) ratio to facilitate the removal of waxy components. The mixture was subsequently stored at −80 °C for 48 h to promote wax precipitation. After cold precipitation, the sample was filtered through filter paper using a vacuum filtration system to remove the precipitated wax fractions. To further purify the cannabinoid-rich extract, molecular distillation was applied to remove volatile terpenes and chlorophyll derivatives co-extracted during the supercritical CO_2_ process. The fraction enriched in phytocannabinoids was collected within the distillation temperature range of 180–200 °C. The obtained distillate fractions were subsequently purified using flash chromatography (BUCHI Pure C815 Flash Chromatography System, BÜCHI Labortechnik AG, Flawil, Switzerland) equipped with C18 reverse-phase cartridges (40 g). An ethanol–water mobile phase was employed for chromatographic separation. The resulting eluates were monitored using UV detection between 254 and 400 nm, and four distinct phytocannabinoid fractions (Frac1, Frac2, Frac3, and Frac4) were collected according to their characteristic UV absorption profiles (Figure 1).

The phytocannabinoid-enriched fractions were analyzed by the GC-MS method (File S1). The sample preparation consisted of hot ethanol extraction (80 °C) followed by GC–MS analysis on a Shimadzu GCMS-QP2010 Ultra (Shimadzu Corporation, Kyoto, Japan) equipped with an Rtx-5MS (30 m × 0.25 mm, 0.25 µm) column. The temperature program ramped from 40 °C to 100 °C and then to 270 °C, with MS acquisition in full-scan mode (*m*/*z* 45–650) between 2.00–120.00 min.

GC–MS analysis revealed distinct compositional profiles among Frac1–Frac4 (Table 2; File S1). Relative abundances are reported as chromatographic peak-area percentages. Frac1 was dominated by 3-propyl-cannabidiol (61.41%), followed by cannabidiol (21.20%) and dronabinol (8.10%), whereas cannabichromene represented 0.12% of the total chromatographic peak area. Frac2 showed a different profile, with cannabichromene (36.14%) and cannabidiol (19.18%) among the major detected constituents. Δ8-Tetrahydrocannabinol was identified as two chromatographic peaks at RT 86.793 and 96.230 min, representing 17.28% and 5.66%, respectively, while 11-hydroxy-Δ9-tetrahydrocannabinol accounted for 2.82%. Frac3 was dominated by 1,3-benzenediol, 5-pentyl- (49.98%), with other prominent constituents including trans-Z-α-bisabolene epoxide (8.85%), illudol (8.68%), cannabispiran (8.15%), and cannabichromene (4.83%). In contrast, Frac4 was dominated by caryophyllene oxide (52.99%), followed by 3-propyl-cannabidiol (12.87%) and 2,4a,8,8-tetramethyldecahydrocyclopropa[d]naphthalene (9.81%); guaiol and cannabichromene accounted for 3.42% and 0.38%, respectively. These results demonstrate that the four fractions had distinct GC–MS compositional profiles rather than representing a concentration series of the same phytochemical mixture.

The GC–MS data were used for compositional profiling, and the relative abundances of detected constituents were expressed as chromatographic peak-area percentages. Absolute concentrations of individual phytocannabinoids were not determined using authentic calibration standards. Therefore, the reported percentages represent relative chemical composition rather than absolute phytocannabinoid concentrations.

### 2.4. Hemp Fiber (Hf) Extraction

Hemp fibers used in this study were supplied by the Yozgat Bozok University Hemp Research Institute. After harvesting, hemp stalks were dried under controlled conditions to equilibrate moisture content and subsequently separated from the fiber bundles. The obtained fibers were cut into pieces approximately 2–3 mm in length and boiled in distilled water for 12 h to remove soluble impurities. The samples were then filtered using filter paper and dried at 80 °C for 30 min. The dried fibers were treated with 30% NaOH solution at 90 °C for 5 h in order to remove non-cellulosic components such as hemicellulose and lignin [17]. After alkaline treatment, the fibers were filtered and dried in an oven at 80 °C for 24 h. The dried material was then subjected to 35% sulfuric acid treatment at boiling temperature for 4 h, followed by filtration and drying at 80 °C for 24 h. Subsequently, the dried fibers were ground into powder using a laboratory blender. The powdered material was further treated with 2 M HCl at boiling temperature for 5 h to remove residual inorganic impurities. The samples were filtered and dried in an oven at 90 °C for 24 h. Finally, the bleaching process was carried out by immersing the fibers in hydrogen peroxide (H_2_O_2_) solution for 1 h at room temperature. After bleaching, the samples were washed thoroughly with distilled water, filtered, dried at 90 °C for 24 h, and sieved through a 0.05 mm mesh filter to obtain purified hemp fiber powder.

### 2.5. Silk Fibroin (Sf) Extraction

Silk cocoons obtained from KOZABİRLİK Agricultural Cooperative were cut into small pieces using scissors to facilitate processing. To remove sericin proteins, 5 g of cocoon fragments was boiled in 0.5% (*w*/*v*) Na_2_CO_3_ solution for 60 min. After the degumming process, the resulting silk fibroin fibers (SF) were thoroughly rinsed three times with deionized water to eliminate residual sericin and other impurities. The purified fibroin fibers were subsequently dissolved in Ajisawa’s ternary solvent system (CaCl_2_/ethanol/water; molar ratio 1:2:8) under boiling conditions until complete dissolution occurred (approximately 1 h). The resulting fibroin solution was transferred into a 5 kDa molecular weight cut-off dialysis membrane and dialyzed sequentially to remove salts and solvent residues. Dialysis was performed against 4 M urea solution for 4 h, followed by 2 M urea for 4 h, 1 M urea for 4 h, and finally distilled water (0 M urea) for 24 h. The resulting silk fibroin solution was measured by UV-Vis and found to have a final concentration of approximately 23 mg/mL, after which it was lyophilized (freeze-dried) and stored under vacuum at +4 °C for later use [18].

### 2.6. Fabrication of Experimental Pmma Composites

The experimental composites were fabricated using the conventional compression molding heat-polymerization technique. Prior to monomer addition, reinforcing agents were dry blended with the PMMA polymer powder according to the designated experimental formulation. For the PMMA/HF group, hemp fiber was incorporated at 1 wt%. The 1 wt% concentration refers to the total mass of each phytochemical fraction incorporated into the PMMA formulation and should not be interpreted as an equivalent concentration of individual phytocannabinoids across Frac1–Frac4, because the fractions differed in chemical composition. For the PMMA/SF group, silk fibroin was incorporated at 1 wt%. In the hybrid reinforcement group (PMMA/HF/SF), hemp fiber and silk fibroin were incorporated at a combined concentration of 1 wt%. Subsequently, phytocannabinoid-rich fractions (Frac1–Frac4) were added at a constant concentration of 1 wt% to the designated experimental groups. The reinforcing concentration was standardized at 1 wt% based on previous studies reporting that low concentrations of natural fibers provide effective reinforcement of PMMA without negatively affecting polymerization kinetics, handling characteristics, or structural integrity. Higher reinforcement levels have been associated with fiber agglomeration, increased porosity, impaired wetting of the polymer matrix, and deterioration of mechanical performance. Likewise, phytocannabinoid-rich fractions were incorporated at a constant concentration of 1 wt% to facilitate direct comparison among the different phytochemical formulations while minimizing concentration-dependent variability and ensuring consistency throughout the experimental design [9].

Dry blending was performed using a laboratory mixer (WF-OD20, Weightlab Instruments, İstanbul, Türkiye) for 10 min until a visually homogeneous powder mixture was obtained. Particular attention was paid to minimizing fiber agglomeration and promoting uniform mixing of the reinforcing fibers and phytocannabinoid fractions before monomer addition. The modified polymer powder was then combined with the monomer liquid according to the manufacturer’s recommended powder-to-liquid ratio and mixed until the dough stage was reached. The dough was packed into dental stone molds using the conventional compression molding technique. Trial packing procedures were repeated until complete mold adaptation was achieved.

Polymerization was carried out according to the manufacturer’s recommended heat-curing cycle. Following polymerization, the flasks were allowed to cool slowly to room temperature before deflasking to minimize residual internal stresses generated during polymerization.

The specimens were finished using tungsten carbide burs followed by sequential polishing with silicon carbide abrasive papers under continuous water irrigation. Final polishing procedures were standardized for all specimens to minimize surface variability before mechanical and biological testing.

All specimens were fabricated by the same operator under identical laboratory conditions. Specimen dimensions were verified using a digital caliper with an accuracy of 0.01 mm. All specimens were visually inspected before testing. Samples exhibiting visible porosity, dimensional deviations, polymerization defects, or surface imperfections were excluded and replaced. Before testing, all specimens were immersed in distilled water at 37 ± 1 °C for 48 h to permit residual monomer release.

### 2.7. Experimental Groups

Twenty experimental groups were established according to the type of reinforcement and phytocannabinoid formulation. Four different PMMA material systems were evaluated: unmodified PMMA, PMMA reinforced with hemp fiber (PMMA/HF), PMMA reinforced with silk fibroin (PMMA/SF), and PMMA reinforced with a hybrid combination of hemp fiber and silk fibroin (PMMA/HF/SF). Each material system consisted of five subgroups according to the incorporated phytocannabinoid formulation (Control, Frac1, Frac2, Frac3, and Frac4).

The control subgroup contained no phytocannabinoid fraction, whereas the remaining experimental subgroups contained 1 wt% of the corresponding phytocannabinoid-rich fraction. A detailed description of the experimental groups is presented in Table 3.

### 2.8. Specimen Preparation

Specimens were fabricated according to the dimensional requirements specified by the corresponding ISO and ASTM standards for each mechanical and biological evaluation. Independent specimen sets were prepared for flexural strength, elastic modulus, Charpy impact strength, Vickers surface hardness, structural characterization (FTIR, XRD, and SEM), antimicrobial assessment, and cytocompatibility analysis to eliminate any influence of previous testing procedures on subsequent analyses.

Independent specimen sets were prepared for the different experimental assays to avoid potential effects of prior testing on subsequent measurements. For mechanical characterization, 10 independent specimens were used per experimental group. Flexural strength and elastic modulus were determined from the same three-point bending specimens (n = 10 per group; 20 groups, 200 specimens). Separate sets of 10 independent specimens per group were prepared for Charpy impact strength (200 specimens) and Vickers hardness (200 specimens). For Vickers hardness, three indentations were performed on each specimen and averaged to obtain a single specimen-level value; these indentations were considered technical measurements and were not treated as independent replicates. Biological experiments were conducted independently of the mechanical tests. The MTT bacterial viability and LDH cytotoxicity assays were each performed as three independent experiments (n = 3). Sample sizes and replicate types for the individual assays are additionally specified in the corresponding methodological subsections.

### 2.9. Mechanical Characterization

#### 2.9.1. Flexural Strength and Elastic Modulus

Flexural strength and elastic modulus were evaluated using a three-point bending test, which is considered the standard method for assessing the mechanical performance of denture base polymers under simultaneous tensile and compressive loading conditions. These parameters provide important information regarding the material’s resistance to functional deformation and fracture during mastication.

Bar-shaped specimens (64 × 10 × 3.3 mm) were fabricated according to ISO 1567:1999 [19]. specifications for denture base polymers. Before testing, all specimen dimensions were verified using a digital caliper with an accuracy of 0.01 mm. The specimens were positioned on two parallel supports with a span length of 50 mm and loaded at the midpoint using a universal testing machine (Instron Model 8871, Instron Corp., Canton, MA, USA). A crosshead speed of 5 mm/min was applied until specimen fracture occurred.

The maximum fracture load (N) was automatically recorded by the testing software, and flexural strength (FS, MPa) was calculated using the following equation:

***FS*** = **3*FL***/**2*bd*^2^**

where **F** represents the maximum fracture load (N), **L** is the support span (mm), **b** is the specimen width (mm), and **d** is the specimen thickness (mm).

Elastic modulus (E, GPa) was determined from the linear elastic portion of the load–deflection curve using the following equation:

***E*** = ***FL*^3^**/**4*Ybd*^3^**

where **F** is the applied load within the proportional limit (N), **L** is the support span (mm), **Y** is the corresponding deflection (mm), **b** is the specimen width (mm), and **d** is the specimen thickness (mm). Ten independent specimens were tested per experimental group (n = 10). Flexural strength and elastic modulus were obtained from the same specimens.

#### 2.9.2. Charpy Impact Strength Results

Charpy impact strength testing was performed to evaluate the ability of the experimental composites to absorb sudden impact energy and resist catastrophic fracture, which represents one of the most common causes of denture failure during accidental dropping.

Rectangular specimens (55 × 10 × 10 mm) incorporating a standardized 2-mm central notch were prepared according to ASTM D256 [20] and British Standard BS 2487 [21]. recommendations for denture base polymers. Impact testing was performed using a digital Charpy impact testing machine (XJU 5.5, Jinan Hensgrand Instrument Co., Ltd., Jinan, China). Each specimen was horizontally positioned on the supporting anvils, and impact loading was applied at the midpoint opposite the notch until fracture occurred.

The absorbed fracture energy was recorded automatically, and impact strength (kJ/m^2^) was calculated using the following equation:

***Impact Strength*** = ***Ec***/***WT***

where **Ec** represents the corrected absorbed fracture energy (kJ), **W** is the specimen width (m), and **T** is the specimen thickness (m). Ten independent specimens were tested per experimental group (n = 10).

#### 2.9.3. Vickers Surface Hardness Results

Surface hardness was determined using the Vickers microhardness test to evaluate the resistance of the experimental composites to localized surface deformation. Square specimens measuring 12 × 12 × 3 mm were fabricated and polished under standardized conditions before testing.

Hardness measurements were performed using a Vickers hardness tester (Wilson Hardness, ITW Test & Measurement GmbH, Shanghai, China) equipped with a diamond pyramidal indenter. A load of 25 gf was applied for 30 s under identical testing conditions for all experimental groups. Three indentations were made on each specimen at separate locations while maintaining sufficient spacing between adjacent indentations to avoid interaction of the deformation fields.

The Vickers hardness number (VHN) was calculated automatically by the instrument software, and the mean value of the three measurements was recorded as the final hardness value for each specimen. Ten independent specimens were tested per experimental group (n = 10). Three indentations were performed on each specimen and averaged to obtain one specimen-level hardness value; therefore, the three indentations were considered technical measurements and were not treated as independent replicates.

### 2.10. Structural Characterization Results

#### 2.10.1. Attenuated Total Reflection Fourier Transform Infrared Spectroscopy (ATR–FTIR)

ATR–FTIR analysis was experimentally performed using an FT/IR-6300 spectrometer (JASCO, Tokyo, Japan) equipped with a diamond ATR crystal. Spectra were collected over the range of 4000–400 cm^−1^ at a spectral resolution of 4 cm^−1^ using 32 accumulated scans per specimen under identical ambient conditions. ATR–FTIR measurements were performed for all four material systems (PMMA, PMMA/HF, PMMA/SF, and PMMA/HF/SF), including their corresponding Control, Frac1, Frac2, Frac3, and Frac4 formulations. The obtained spectra were compared to identify characteristic functional-group absorptions and to evaluate spectral changes associated with fiber reinforcement and phytocannabinoid incorporation.

#### 2.10.2. X-Ray Diffraction (XRD)

X-ray diffraction (XRD) analysis was performed using an X-ray diffractometer (Rigaku, Tokyo, Japan) to evaluate the crystalline structure of pure PMMA, PMMA/HF, PMMA/SF, and PMMA/HF/SF specimens containing phytocannabinoid fractions. Diffraction patterns were recorded over a 2θ range of 10–50° using Cu Kα radiation (λ = 1.5406 Å), operating at 40 kV and 30 mA, with a step size of 0.02° and a scanning speed of 4°/min at room temperature.

#### 2.10.3. Scanning Electron Microscopy (SEM)

Scanning electron microscopy (SEM) was performed to examine the morphology of *S. mutans* and *L. acidophilus* biofilms formed on the surfaces of PMMA/HF/SF specimens containing different phytocannabinoid fractions. Following biofilm formation, the specimens were washed twice with Dulbecco’s phosphate-buffered saline (DPBS) and fixed in 2.5% glutaraldehyde prepared in phosphate buffer for 16 h, followed by postfixation in 2% osmium tetroxide for 2 h. The specimens were subsequently dehydrated through a graded ethanol series (30%, 50%, 90%, 95%, and 100%), mounted on aluminum stubs, and sputter-coated with an approximately 10 nm thick gold layer. Surface morphology was examined using a scanning electron microscope (Zeiss LEO 440, Carl Zeiss, Oberkochen, Germany) operated at an accelerating voltage of 15 kV, a working distance of 10–12 mm, and magnifications ranging from 1000× to 25,000×.

### 2.11. Biological Characterization Results

#### 2.11.1. Susceptibility Testing Results

The MTT assay was used to estimate the relative metabolic activity of viable biofilm-associated bacterial cells on the experimental material surfaces. MTT-derived absorbance was therefore interpreted as relative bacterial viability and was not used as a direct measure of biofilm biomass or architecture. Thus, we performed an MTT assay according to the literature [22]. For MTT, HF/SF spiked samples containing different phytocannabinoids were collected, and a biofilm layer was formed on them. The samples were then added to a 24-well plate. Microbiologics provided the bacterial culture, which was obtained in KWIK-STIK format. Each KWIK-STIK unit includes a lyophilized microorganism pellet, a hydrating fluid ampoule, and an inoculating swab. During the experiment, 1-part 5 mg/mL MTT is mixed with nine parts medium (BHI Broth for *S. mutans* and MRS Broth for *L. acidophilus*) and used. At the end of the incubation period, the old medium was withdrawn. MTT solution prepared with the same medium (1 mL here) was added and incubated in the dark for at least 6 h in the incubator. Then, 900 µL of MTT solution was withdrawn, and 800 µL DMSO and 100 µL Sorenson’s glycine buffer (glycine 0.1M, NaCl 0.1M, pH 10.5) were added to the medium and left on the mixer in the dark for 15–20 min. Samples were loaded onto a 96-well plate without a lid and read at 570–630 nm on a microplate reader. The absorbance values obtained were used to calculate the percentage viability using the Excel™ program. All experiments were performed in three independent biological replicates (n = 3), and the results are presented as mean ± standard deviation (SD).

#### 2.11.2. Cytotoxicity Test

The Beas2B (RRID, CVCL-0168, American Type Culture Collection, Manassas, VA, USA)) normal lung epithelial cell line was used. The Beas2B cells used in this study were donated by Prof. Dr. Şaban Tekin. BEAS-2B cells were used as a non-tumorigenic epithelial screening model to evaluate the general cytotoxic potential of the experimental formulations; they were not intended to represent an oral tissue-specific cellular model. LDH-derived cytotoxicity was evaluated in BEAS-2B cells using the Cytotoxicity Detection Kit (LDH) (Roche, Basel, Switzerland, Cat. No. 11644793001) according to the manufacturer’s instructions. This colorimetric assay quantifies LDH activity released from the cytosol of membrane-damaged cells into the culture supernatant. BEAS-2B cells were cultured in supplemented DMEM containing 10% FBS and 2% PenStrep at 37 °C in a humidified atmosphere containing 5% CO_2_ and were seeded at 5000 cells per well. After approximately 16 h of pre-incubation, the experimental specimens, with dimensions as described in the Specimen Preparation section, were placed directly in contact with the cells in 100 µL of culture medium. Following 24 h of exposure, LDH release into the culture supernatant was determined using the manufacturer’s reaction procedure, and absorbance was measured at 490 nm. Low-release and high-release controls were included according to the manufacturer’s protocol and served as the negative and positive cytotoxicity controls, respectively. Cytotoxicity was calculated as: % Cytotoxicity = [(A_sample − A_low control)/(A_high control − A_low control)] × 100, where A_sample, A_low control, and A_high control represent the absorbance values of the experimental sample, low-release control, and high-release control, respectively. Each experimental condition was evaluated in three independent biological experiments (n = 3), with no additional technical replicates. Results are expressed as mean ± SD.

### 2.12. Statistical Analysis

All statistical analyses were conducted at a significance level of α = 0.05. Initial analyses were performed using SPSS version 17.0 (SPSS Inc., Chicago, IL, USA), whereas factorial reanalyses, exact probability calculations, post-hoc analyses, and robustness checks performed during revision were independently verified using Python-based (version 3.12.9) statistical libraries (SciPy (version 1.11.4), and statsmodels (version 0.14.4)). Normality was evaluated using the Shapiro–Wilk test and examination of model residuals, while homogeneity of variances was assessed using Levene’s test.

The mechanical dataset followed a balanced factorial design with two independent factors: material system at four levels (PMMA, PMMA/SF, PMMA/HF, and PMMA/HF/SF) and phytocannabinoid condition at five levels (Control, Frac1, Frac2, Frac3, and Frac4), with 10 independent specimens per experimental cell. Flexural strength, elastic modulus, Vickers hardness, and Charpy impact strength were analyzed separately using two-way factorial ANOVA, including the main effects of the material system and phytocannabinoid condition and their interaction. Effect sizes were expressed as partial eta squared (partial η^2^). When a significant interaction was detected, simple-effects analyses were performed to compare material systems within each phytocannabinoid condition and phytocannabinoid conditions within each material system, followed by Tukey-adjusted pairwise comparisons. For outcomes without a significant interaction, interpretation was based primarily on the factorial main effects, with Tukey-adjusted comparisons of marginal means where appropriate. Detailed factorial and post-hoc outputs are provided in Supplementary File.

For the mechanical outcomes, none of the 20 individual experimental cells showed a significant departure from normality (all cell-wise Shapiro–Wilk *p* > 0.05). Although Shapiro–Wilk tests of the pooled model residuals were statistically significant for flexural strength (*p* = 2.383 × 10^−5^), elastic modulus (*p* = 5.599 × 10^−5^), Vickers hardness (*p* = 4.836 × 10^−6^), and impact strength (*p* = 1.107 × 10^−6^), Q–Q diagnostics and standardized residuals indicated only modest departures from normality. Homogeneity of variances across the 20 experimental cells was supported for elastic modulus [F(19,180) = 0.142, *p* = 0.999994], Vickers hardness [F(19,180) = 0.003, *p* ≈ 1.000000], and impact strength [F(19,180) = 0.008, *p* ≈ 1.000000], but not for flexural strength [F(19,180) = 2.325, *p* = 0.002184]. Because the design was fully balanced with equal sample sizes in all cells (n = 10), factorial ANOVA was retained. For flexural strength, an additional HC3 heteroskedasticity-robust sensitivity analysis confirmed the inferential conclusions, including the significant material system × phytocannabinoid condition interaction (F = 5.212, *p* = 1.717 × 10^−7^).

For MTT-derived bacterial metabolic viability, *L. acidophilus* and *S. mutans* were analyzed separately. Each dataset followed a balanced 4 × 5 factorial design comprising four material systems and five phytocannabinoid conditions, with three independent biological experiments per experimental cell (n = 3). Factorial-model residuals did not show significant departures from normality for either *L. acidophilus* (Shapiro–Wilk *p* = 0.293766) or *S. mutans* (*p* = 0.085747), and homogeneity of variances was supported by Levene’s test (*p* = 0.997600 and *p* = 0.999503, respectively). Accordingly, MTT-derived viability was analyzed using two-way factorial ANOVA to evaluate the main effects of the material system and phytocannabinoid condition and their interaction. Because significant interactions were detected for both microorganisms, simple-effects analyses followed by Tukey-adjusted pairwise comparisons were performed. Effect sizes were expressed as partial η^2^.

For LDH-derived cytotoxicity, the dataset followed a balanced 4 × 5 factorial design comprising four material systems and five phytocannabinoid conditions, with three independent biological experiments per experimental cell (n = 3). Factorial-model residuals showed a statistically significant departure from normality (Shapiro–Wilk W = 0.8976, *p* = 0.000108), whereas homogeneity of variances across the 20 experimental cells was strongly supported by Levene’s test [F(19,40) = 0.305, *p* = 0.996395]. Given the fully balanced factorial design and the absence of variance heterogeneity, two-way factorial ANOVA was used, with the departure from residual normality explicitly considered when interpreting the results. The analysis included the main effects of the material system and phytocannabinoid condition and their interaction. Because the interaction was significant, simple-effects analyses followed by Tukey-adjusted pairwise comparisons were performed. Effect sizes were expressed as partial η^2^.

Differences were considered statistically significant at *p* < 0.05. Quantitative data are presented as mean ± standard deviation (SD).

## 3. Results

### 3.1. Mechanical Characterization Results

The mechanical properties of the experimental groups and the corresponding two-way factorial ANOVA results are summarized in Table 4. Overall, the material system and phytocannabinoid condition significantly affected all evaluated mechanical properties. Significant material system × phytocannabinoid condition interactions were observed for flexural strength, Vickers hardness, and impact strength, whereas no significant interaction was detected for elastic modulus.

#### 3.1.1. Flexural Strength

Two-way factorial ANOVA demonstrated significant main effects of the material system [F(3,180) = 4277.137, *p* = 5.173 × 10^−167^, partial η^2^ = 0.986] and phytocannabinoid condition [F(4,180) = 342.180, *p* = 6.068 × 10^−83^, partial η^2^ = 0.884] on flexural strength. A significant material system × phytocannabinoid condition interaction was also detected [F(12,180) = 2.778, *p* = 0.001700, partial η^2^ = 0.156], indicating that the magnitude of the phytocannabinoid-associated reduction in flexural strength varied according to the material system. Simple-effects analyses demonstrated significant differences among material systems at every phytocannabinoid condition (exact *p*-values ranging from 4.886 × 10^−111^ to 1.402 × 10^−100^), with the consistent ranking PMMA/HF/SF > PMMA/HF > PMMA/SF > PMMA. Within PMMA, PMMA/SF, and PMMA/HF, Control and Frac1 did not differ significantly (Tukey-adjusted *p* = 0.313398, 0.240001, and 0.116727, respectively); in PMMA/HF/SF, Control differed significantly from Frac1 (adjusted *p* = 0.004). Detailed pairwise results are provided in Supplementary File S2.

Among all groups, PMMA/HF/SF-Control exhibited the highest flexural strength (138.04 ± 2.05 MPa), whereas PMMA-Frac4 showed the lowest value (83.31 ± 0.85 MPa). Relative to PMMA-Control (94.59 ± 2.46 MPa), flexural strength was higher in PMMA/SF-Control (111.02 ± 2.88 MPa), PMMA/HF-Control (128.80 ± 3.35 MPa), and PMMA/HF/SF-Control (138.04 ± 2.05 MPa).

Within each material system, flexural strength generally decreased from Control toward Frac4. The significant interaction indicates that this reduction was not identical across material systems; nevertheless, PMMA/HF/SF consistently showed the highest flexural strength at each phytocannabinoid condition.

#### 3.1.2. Elastic Modulus

Elastic modulus was significantly affected by the material system [F(3,180) = 810.121, *p* = 3.073 × 10^−104^, partial η^2^ = 0.931] and phytocannabinoid condition [F(4,180) = 1428.581, *p* = 3.812 × 10^−135^, partial η^2^ = 0.969]. The material system × phytocannabinoid condition interaction was not significant [F(12,180) = 0.006, *p* = 1.000000, partial η^2^ < 0.001], indicating that the phytocannabinoid-associated reduction in elastic modulus followed an essentially parallel pattern across the four material systems.

The highest elastic modulus was recorded for PMMA/HF/SF-Control (2.519 ± 0.017 GPa), whereas PMMA-Frac4 demonstrated the lowest stiffness (2.086 ± 0.021 GPa). Tukey-adjusted comparisons of material-system marginal means indicated significant differences among all four material systems, including the smallest contrast between PMMA and PMMA/HF (adjusted *p* = 0.042656). All five phytocannabinoid-condition marginal means also differed significantly from one another (adjusted *p* < 0.001).

Because no interaction was detected for elastic modulus, the effects of the material system and phytocannabinoid condition were interpreted primarily as independent main effects rather than condition-specific simple effects.

#### 3.1.3. Vickers Surface Hardness

Vickers hardness was significantly affected by the material system [F(3,180) = 7369.351, *p* = 4.761 × 10^−188^, partial η^2^ = 0.992] and phytocannabinoid condition [F(4,180) = 1426.583, *p* = 4.307 × 10^−135^, partial η^2^ = 0.969]. A significant material system × phytocannabinoid condition interaction was also identified [F(12,180) = 9.480, *p* = 3.956 × 10^−14^, partial η^2^ = 0.387]. Simple-effects analyses showed significant differences among all four material systems at every phytocannabinoid condition (exact *p*-values ranging from 5.730 × 10^−132^ to 1.307 × 10^−117^), with PMMA/HF/SF > PMMA/HF > PMMA/SF > PMMA. Within every material system, Tukey-adjusted comparisons showed a significant stepwise decrease in hardness in the order Control > Frac1 > Frac2 > Frac3 > Frac4.

PMMA/HF/SF-Control exhibited the highest surface hardness (26.14 ± 0.22 VHN), whereas PMMA-Frac4 demonstrated the lowest hardness (16.54 ± 0.23 VHN). The significant interaction indicates that the magnitude of the phytocannabinoid-associated reduction in hardness depended on the material system, although the direction of change remained consistent across systems.

Although reductions in hardness were observed with increasing phytocannabinoid formulation, the hybrid reinforcement groups consistently exhibited significantly higher hardness values than the corresponding PMMA groups (*p* < 0.05).

#### 3.1.4. Charpy Impact Strength

Charpy impact strength was significantly affected by the material system [F(3,180) = 14511.601, *p* = 2.231 × 10^−214^, partial η^2^ = 0.996] and phytocannabinoid condition [F(4,180) = 2036.961, *p* = 1.192 × 10^−148^, partial η^2^ = 0.978]. A significant material system × phytocannabinoid condition interaction was also observed [F(12,180) = 11.417, *p* = 7.123 × 10^−17^, partial η^2^ = 0.432]. Simple-effects analyses confirmed differences among material systems at every phytocannabinoid condition (exact *p*-values ranging from 2.064 × 10^−157^ to 1.388 × 10^−145^). At each phytocannabinoid condition, all four material systems differed significantly (all Tukey-adjusted *p* < 0.001), with PMMA/HF/SF > PMMA/HF > PMMA/SF > PMMA. Within each material system, all Tukey-adjusted pairwise comparisons among Control and Frac1-Frac4 were significant, with impact strength decreasing in the order Control > Frac1 > Frac2 > Frac3 > Frac4.

The highest impact strength was obtained for PMMA/HF/SF-Control (24.22 ± 0.22 kJ/m^2^), whereas PMMA-Frac4 exhibited the lowest value (11.82 ± 0.20 kJ/m^2^). Compared with PMMA-Control (15.05 ± 0.21 kJ/m^2^), PMMA/SF-Control and PMMA/HF-Control exhibited higher impact strength values of 18.02 ± 0.22 and 21.15 ± 0.22 kJ/m^2^, respectively.

Although impact strength decreased progressively from Control to Frac4 within each material system, the significant interaction indicates that the magnitude of this decrease varied according to the material system. PMMA/HF/SF nevertheless maintained the highest impact strength at every phytocannabinoid condition.

### 3.2. Structural Characterization

#### 3.2.1. ATR-FTIR Analysis

The FTIR/ATR spectra presented in Figure 2 and Figure 3 provide detailed information about the chemical structure of the PMMA-based composites containing cannabinoid fractions (Frac1–Frac4) and different natural biopolymer components.

In both Figure 2A (PMMA/HF/SF) and Figure 2B (PMMA/SF), all samples retained the dominant PMMA absorptions, especially the strong ester carbonyl band at about 1720–1754 cm^−1^ and the aliphatic C–H stretching region at 2849–2917 cm^−1^ [23,24]. Bands around 1463–1499 cm^−1^ and 1365–1366 cm^−1^ were also maintained, consistent with CH bending modes of the methacrylate matrix. In the fibroin-containing systems, features in the amide region, particularly near 1597–1625 cm^−1^, supported the contribution of silk fibroin [25,26]. In PMMA/HF/SF, additional signals around 1006–1145 cm^−1^ were more pronounced, consistent with C–O and C–O–C vibrations from hemp-derived lignocellulosic constituents [27,28]. Compared with the control, fractions F1–F4 caused modest shifts in the carbonyl and fingerprint regions and altered relative intensities of peaks near 1217–1243 cm^−1^ and 1722–1756 cm^−1^, indicating matrix–fraction interactions without the appearance of clear new bands that would suggest formation of new covalent bonds [29].

FTIR/ATR spectra of PMMA/HF composites (Figure 3A) and pure PMMA systems (Figure 3B) exhibited characteristic vibrational bands of polymethyl methacrylate and hemp fiber constituents. A strong absorption band at 1722–1754 cm^−1^ corresponds to the C=O stretching vibration of PMMA ester groups, confirming the integrity of the polymer matrix across all samples [23,24]. The aliphatic C–H stretching bands at 2849 and 2917 cm^−1^ were consistently present in control and cannabinoid-containing fractions (Frac1–Frac4), reflecting hydrocarbon structures typical of PMMA and terpene-derived cannabinoid compounds. In PMMA/HF composites, additional peaks appeared within 1000–1150 cm^−1^, attributed to C–O–C and C–O stretching vibrations from lignocellulosic polysaccharides in hemp fibers [27]. Bands near 1463–1497 cm^−1^ and 1365 cm^−1^ correspond to CH_2_/CH_3_ bending modes of the methacrylate backbone. Minor spectral shifts and intensity changes were observed in cannabinoid fractions, particularly in the 1217–1243 cm^−1^ and 1720–1750 cm^−1^ regions, suggesting weak intermolecular interactions between cannabinoids and polymer chains without evidence of new covalent bond formation.

Comparative analysis between the control and cannabinoid-containing fractions revealed minor peak shifts and variations in band intensity. These spectral differences suggest weak intermolecular interactions, such as hydrogen bonding and van der Waals forces, between PMMA, fibroin, hemp fiber components, and cannabinoid molecules, rather than the formation of new covalent bonds. Overall, the FTIR data confirm the successful incorporation of cannabinoid fractions into the polymeric composite matrices while maintaining the fundamental structural features of the constituent materials.

#### 3.2.2. X-Ray Diffraction Analysis

The X-ray diffraction (XRD) patterns of pure and cannabinoid-fraction-incorporated polymer composites are presented in Figure 4. All materials, including PMMA/HF/SF (A), PMMA/SF (B), PMMA/HF (C), and pure PMMA (D), exhibited predominantly broad diffraction halos rather than sharp crystalline peaks, indicating that the matrices retained a largely amorphous polymer structure. The principal diffraction maximum for all samples appeared in the 2θ range of approximately 13–17°, which is characteristic of the amorphous packing of polymethyl methacrylate (PMMA) chains. A secondary broad halo was observed around 28–32°, corresponding to short-range ordering within the polymer backbone.

In the PMMA/HF/SF composite (Figure 4A), slight increases in diffraction intensity were observed for cannabinoid fractions, particularly in the Frac3 and control samples, suggesting minor variations in polymer chain packing caused by interactions between PMMA, silk fibroin, hemp fibers, and the incorporated bioactive compounds. The PMMA/SF system (Figure 4B) showed relatively similar diffraction profiles among control and cannabinoid fractions, indicating that the addition of Frac1–Frac4 did not significantly alter the structural organization of the PMMA–fibroin matrix. For PMMA/HF composites (Figure 4C), broader halos with moderate intensity differences were observed, reflecting the semi-disordered structure contributed by lignocellulosic hemp fibers. In contrast, pure PMMA samples (Figure 4D) displayed the most uniform diffraction pattern, confirming the typical amorphous nature of PMMA. Overall, the XRD patterns suggest that incorporation of cannabinoid fractions did not introduce new crystalline phases but slightly modified the short-range molecular ordering and packing density within the polymer composites. These findings indicate that cannabinoids are mainly physically dispersed within the polymer matrices without inducing significant crystallization or structural phase transformation.

#### 3.2.3. Scanning Electron Microscopy

Biofilm morphology, bacterial surface coverage, aggregation, and adhesion were assessed qualitatively by SEM. Scanning electron microscopy (SEM) at 25,000× magnification revealed marked differences in biofilm formation by *L. acidophilus* and *S.mutans* on the experimental PMMA-based composites (Figure 5 and Figure 6). Conventional PMMA specimens without phytocannabinoid incorporation exhibited dense bacterial colonization characterized by continuous surface coverage, extensive bacterial aggregation, and well-developed multilayered biofilm structures.

Compared with conventional PMMA, PMMA/HF and PMMA/SF specimens exhibited visibly reduced bacterial surface coverage and less organized biofilm architecture. The incorporation of phytocannabinoid fractions further decreased bacterial adhesion and disrupted biofilm formation in all material systems. Among all experimental groups, the hybrid PMMA/HF/SF composites demonstrated the lowest degree of bacterial colonization and the sparsest biofilm architecture for both *L. acidophilus* and *S. mutans*, indicating superior antibiofilm performance.

Overall, the SEM observations qualitatively corroborated the MTT viability results by demonstrating that phytocannabinoid incorporation markedly reduced bacterial attachment and biofilm development on the PMMA surface, while hybrid fiber reinforcement maintained this antibacterial performance without compromising the structural integrity of the composite.

### 3.3. Biological Characterization

#### 3.3.1. Susceptibility Testing

The relative metabolic viability of biofilm-associated bacteria was quantified using the MTT assay. Reductions in MTT-derived viability relative to the corresponding controls were interpreted as evidence of antibacterial activity (Table 5). For *L. acidophilus*, two-way factorial ANOVA demonstrated significant effects of the material system [F(3,40) = 2904.299, *p* = 8.095 × 10^−47^, partial η^2^ = 0.995], the phytocannabinoid condition [F(4,40) = 43,495.634, *p* = 3.558 × 10^−72^, partial η^2^ > 0.999], and their interaction [F(12,40) = 194.671, *p* = 1.638 × 10^−31^, partial η^2^ = 0.983] (Table 6). Similarly, *S. mutans* viability was significantly affected by the material system [F(3,40) = 2243.032, *p* = 1.381 × 10^−44^, partial η^2^ = 0.994], the phytocannabinoid condition [F(4,40) = 28,277.512, *p* = 1.951 × 10^−68^, partial η^2^ > 0.999], and the material system × phytocannabinoid condition interaction [F(12,40) = 56.598, *p* = 3.249 × 10^−21^, partial η^2^ = 0.944] (Table 6). Significant interactions were followed by Tukey-adjusted simple-effects comparisons. Detailed MTT assumption diagnostics, factorial ANOVA, simple-effects analyses, and Tukey-adjusted pairwise comparisons are provided in Supplementary File S3.

#### 3.3.2. Cytotoxic Activity of the Modified Pmma Samples

Two-way factorial ANOVA demonstrated significant effects of the material system [F(3,40) = 7.671, *p* = 0.000365, partial η^2^ = 0.365], the phytocannabinoid condition [F(4,40) = 46.390, *p* = 1.651 × 10^−14^, partial η^2^ = 0.823], and their interaction [F(12,40) = 4.113, *p* = 0.000350, partial η^2^ = 0.552] on LDH-derived cytotoxicity. Despite these statistically significant formulation-dependent effects, LDH-derived cytotoxicity remained low across all experimental conditions (1.31–4.73%). Simple-effects analyses showed that the effect of the material system was significant for Frac2 [F(3,8) = 6.396, *p* = 0.016123] and Control [F(3,8) = 28.781, *p* = 0.000123], but not for Frac1 (*p* = 0.130698), Frac3 (*p* = 0.066574), or Frac4 (*p* = 0.370923). Conversely, the phytocannabinoid condition had a significant simple effect within PMMA/HF/SF [F(4,10) = 22.967, *p* = 0.000050], PMMA/SF [F(4,10) = 10.786, *p* = 0.001194], PMMA/HF [F(4,10) = 20.977, *p* = 0.000075], and PMMA [F(4,10) = 6.359, *p* = 0.008215]. Tukey-adjusted pairwise comparisons were subsequently used to resolve these significant simple effects, with the corresponding multiple-comparison groupings presented in Table 7. Collectively, these findings indicate that the significant material system × phytocannabinoid condition interaction was driven by specific formulation-dependent differences rather than by a uniform effect of the material system across all phytocannabinoid conditions. Detailed LDH assumption diagnostics, simple-effects results, and Tukey-adjusted pairwise comparisons are provided in Supplementary File S4.

## 4. Discussion

The null hypothesis was rejected because the incorporation of hemp fiber, silk fibroin, and phytocannabinoid-rich fractions significantly affected the mechanical, structural, and biological properties of PMMA denture base resin. Hybrid reinforcement substantially improved the mechanical performance while preserving the physicochemical integrity and biological functionality of the composite, demonstrating that multifunctional reinforcement successfully enhanced PMMA without compromising its bioactive characteristics. The present study proposed a multifunctional composite design strategy in which hybrid natural fiber reinforcement and phytocannabinoid functionalization were simultaneously integrated into a PMMA denture base resin to overcome one of the major challenges in contemporary prosthetic biomaterials: improving mechanical durability without compromising biological performance. Unlike previous investigations that primarily focused on either mechanical reinforcement or antimicrobial modification separately, the present work combined these two approaches within a single composite architecture and comprehensively evaluated their influence on the mechanical, structural, and biological properties of PMMA [30]. The findings demonstrated that hybrid reinforcement with hemp fiber and silk fibroin substantially enhanced the mechanical behavior of the denture base resin while preserving the characteristic chemical structure, amorphous polymer architecture, antibacterial performance, and cytocompatibility of the material. Collectively, these observations suggest that multifunctional composite engineering represents a promising strategy for the development of next-generation denture base biomaterials [31,32].

One of the most important findings of the present investigation was that the PMMA/HF/SF formulation consistently produced superior mechanical performance compared with the corresponding single-fiber formulations and unmodified PMMA. The factorial analysis further showed strong main effects of the material system and phytocannabinoid condition for all four mechanical outcomes. Significant material system × phytocannabinoid condition interactions were detected for flexural strength, surface hardness, and impact strength, demonstrating that the magnitude of the phytocannabinoid-associated mechanical change depended on the reinforcement system for these outcomes. In contrast, elastic modulus showed no interaction, indicating essentially independent and parallel effects of the material system and phytocannabinoid condition. The superior performance of the hybrid formulation therefore supports a beneficial complementary effect of combined hemp fiber and silk fibroin reinforcement; however, it should not be interpreted as formal statistical proof of HF × SF synergy because the study was not designed as an independent HF-factor × SF-factor factorial experiment. This interpretation is consistent with composite material theory, in which different reinforcing constituents may contribute complementary load-transfer, crack-arrest, and interfacial mechanisms [30,31,32,33].

The substantial improvement observed in flexural strength is particularly relevant because flexural loading closely simulates the complex tensile and compressive stresses experienced by denture bases during mastication [33]. Conventional PMMA behaves as a relatively brittle polymer in which microscopic defects generated during processing gradually propagate under repeated functional loading until catastrophic fracture occurs [34]. The incorporation of hemp fiber altered this fracture mechanism by allowing a portion of the externally applied stress to be transferred from the PMMA matrix to the reinforcing fibers, thereby reducing stress accumulation within the polymer itself [35]. Silk fibroin further complemented this mechanism by improving the interfacial adhesion between the reinforcing phase and the surrounding polymer matrix, facilitating more homogeneous stress distribution throughout the composite [36]. Consequently, the hybrid reinforcement system demonstrated substantially greater resistance to bending failure than conventional PMMA. Similar reinforcing mechanisms have been described for natural fiber-reinforced polymer composites, in which efficient stress transfer, crack bridging, and interfacial strengthening collectively improve flexural performance [37]. However, unlike previous reports that investigated isolated fiber systems, the present study demonstrates that combining hemp fiber and silk fibroin within a phytocannabinoid-functionalized PMMA matrix provides a more balanced reinforcement strategy capable of simultaneously maintaining mechanical integrity and biological functionality [38].

From a clinical materials perspective, the mechanical findings should also be considered in relation to established denture-base requirements and reference values rather than solely through comparisons among the experimental formulations. ISO 20795-1:2013 [39]. specifies a minimum ultimate flexural strength of 65 MPa and a minimum flexural modulus of 2.0 GPa for heat-polymerized denture-base polymers. In the present study, flexural strength ranged from 83.31 to 138.04 MPa and elastic modulus from 2.076 to 2.519 GPa, indicating that all experimental formulations, including those showing phytocannabinoid-associated reductions, remained above these ISO thresholds. For Vickers hardness and Charpy impact strength, ISO 20795-1 does not establish directly comparable universal minimum acceptance values; therefore, these properties are more appropriately interpreted relative to published values for conventional heat-polymerized PMMA [19]. Previous investigations of heat-polymerized Meliodent have reported a Vickers hardness of approximately 18.1 VHN and impact-strength values of approximately 14.8–16.6 kJ/m^2^. In the present study, Vickers hardness ranged from 16.54 to 26.14 VHN and impact strength from 11.82 to 24.22 kJ/m^2^. Thus, although some unreinforced phytocannabinoid-containing PMMA formulations showed hardness or impact-strength values below conventional PMMA reference values, fiber reinforcement—particularly the hybrid PMMA/HF/SF system—maintained or exceeded these literature benchmarks. These comparisons indicate that the phytocannabinoid-associated reductions should be interpreted in the context of the reinforcing system: while biological functionalization reduced some mechanical parameters, the hybrid reinforcement preserved flexural properties above the applicable ISO requirements and maintained favorable hardness and impact performance relative to conventional heat-polymerized PMMA [40].

Elastic modulus followed a highly consistent pattern across the four material systems. The two-way ANOVA identified strong main effects of both the material system and phytocannabinoid condition but no material system × phytocannabinoid condition interaction. Thus, although phytocannabinoid incorporation progressively reduced stiffness, the magnitude of this effect was essentially parallel across reinforcement systems. From a biomechanical perspective, elastic modulus reflects the ability of a denture base material to resist elastic deformation during functional loading and therefore represents an important determinant of dimensional stability under occlusal forces [41]. The higher modulus of the PMMA/HF/SF formulation may reflect the contribution of hemp fibers together with favorable fiber–matrix interactions associated with silk fibroin [42,43]. The absence of statistical interaction for this endpoint argues against attributing the modulus response to a condition-specific synergistic effect and instead supports additive or independent contributions of the material system and phytocannabinoid condition.

The enhanced impact resistance observed in the PMMA/HF/SF composites can be explained by several potentially complementary toughening mechanisms operating simultaneously. Hemp fibers may act as crack-bridging elements that interrupt crack propagation and redistribute impact-induced stresses away from the crack tip [44], while silk fibroin may improve interfacial bonding and stress transfer between the reinforcing phase and the PMMA matrix [45]. The significant material system × phytocannabinoid condition interaction observed for impact strength indicates that the magnitude of the phytocannabinoid-associated reduction differed among material systems. Accordingly, the superior impact resistance of the hybrid formulation is more appropriately described as evidence of a beneficial complementary hybrid-reinforcement effect rather than formal proof of synergy between HF and SF.

An equally important observation was that phytocannabinoid incorporation produced a gradual reduction in impact strength within each reinforcement system. This trend was expected because the introduction of bioactive phytochemical fractions inevitably modifies the continuity of the polymer matrix and may locally interfere with stress transmission under high-rate loading conditions [46]. Nevertheless, despite this progressive reduction, all hybrid PMMA/HF/SF formulations retained substantially higher impact strength than conventional PMMA. This finding indicates that the reinforcing network successfully compensated for the mechanical alterations associated with phytocannabinoid incorporation, thereby preserving the structural reliability of the material. From a biomaterials engineering perspective, this balance between biological functionalization and mechanical robustness represents one of the principal design objectives for next-generation denture base composites [47].

Surface hardness demonstrated a response comparable to those of flexural and impact strength in that both main effects and the material system × phytocannabinoid condition interaction were significant. Hardness is directly associated with resistance to localized plastic deformation and therefore influences wear resistance, surface integrity, and long-term dimensional stability of denture base materials during clinical service [48]. PMMA/HF/SF exhibited the highest hardness at every phytocannabinoid condition, while hardness decreased from Control to Frac4 within each material system. The significant interaction indicates that the magnitude of this reduction varied among material systems. The superior hardness of the hybrid formulation may reflect restriction of localized matrix deformation by the reinforcing architecture, with hemp fibers contributing resistance to indentation and silk fibroin potentially supporting stress distribution at reinforcing interfaces [49,50].

Although phytocannabinoid incorporation produced a progressive reduction in hardness, the decrease remained relatively limited in comparison with the mechanical benefits provided by hybrid reinforcement. These observations indicate that the phytocannabinoid-rich fractions acted primarily as biofunctional additives rather than structural reinforcing agents [51]. Consequently, the overall mechanical behavior of the composite remained predominantly governed by the hybrid fiber network. Such findings are particularly important because they demonstrate that biological functionalization can be introduced into PMMA without eliminating the improvements achieved through rational composite design. Scanning electron microscopy provided qualitative evidence supporting the antimicrobial findings obtained from the MTT viability assay. Control specimens without phytocannabinoid incorporation exhibited dense bacterial colonization characterized by extensive surface coverage and multilayered biofilm formation for both *L. acidophilus* and *S. mutans*. In contrast, all phytocannabinoid-containing formulations demonstrated visibly reduced bacterial adhesion and less organized biofilm architecture. The reduction in bacterial surface colonization became more pronounced in the reinforced composite systems, particularly in the hybrid PMMA/HF/SF formulations, which exhibited the sparsest biofilm coverage among all experimental groups. These qualitative observations were consistent with the quantitative MTT results, confirming that phytocannabinoid incorporation effectively inhibited bacterial attachment and biofilm development on the PMMA surface.

The combined SEM and microbiological findings indicate that the antibacterial performance of the developed composites was primarily associated with the incorporated phytocannabinoid fractions, whereas the hybrid hemp fiber–silk fibroin reinforcement served principally to preserve and enhance the mechanical integrity of the material. Thus, the present multifunctional composite design successfully separated the biological and mechanical functions of the individual components without evidence that biological functionalization compromised the structural performance of the denture base resin. This complementary behavior represents an important advantage for the development of next-generation denture base biomaterials intended to simultaneously improve durability and reduce microbial colonization.

The structural characterization performed by ATR–FTIR and XRD analyses further supports the mechanical findings by demonstrating that the reinforcement strategy did not fundamentally alter the chemical structure or polymer architecture of PMMA. The ATR–FTIR spectra preserved all characteristic absorption bands associated with the polymer matrix, while no additional absorption peaks or substantial spectral shifts were detected following incorporation of hemp fiber, silk fibroin, or phytocannabinoid-rich fractions. These observations suggest that the reinforcing components were physically incorporated within the PMMA matrix rather than participating in chemical reactions capable of modifying the polymerization mechanism [52]. Preservation of the characteristic PMMA functional groups is particularly important because successful reinforcement should enhance material performance without compromising the intrinsic chemical stability of the denture base resin.

The XRD findings provide complementary evidence for this interpretation. All experimental formulations retained the broad amorphous diffraction halo characteristic of heat-polymerized PMMA, and no additional crystalline phases were detected following reinforcement or phytocannabinoid incorporation. Minor variations in diffraction intensity were observed among certain formulations; however, these changes were insufficient to indicate structural transformation of the polymer network. Instead, they most likely reflect minor variations in local molecular packing and the physical distribution of the incorporated reinforcing constituents within the polymer matrix. The preservation of the amorphous polymer architecture is advantageous because excessive crystallization could increase material brittleness and compromise the long-term clinical performance of denture base resins. Therefore, the combined ATR–FTIR and XRD results demonstrate that the proposed multifunctional reinforcement strategy enhanced mechanical performance while maintaining the physicochemical integrity of the PMMA matrix [53].

An important outcome of the present investigation is that the mechanical improvements achieved through hybrid reinforcement were not accompanied by deterioration of biological performance. Denture base materials are continuously exposed to complex oral biofilms; therefore, enhancement of mechanical durability alone is insufficient unless accompanied by appropriate biological behavior. The incorporation of phytocannabinoid-rich fractions reduced bacterial viability while maintaining the favorable mechanical characteristics provided by the hybrid fiber network. These findings indicate that the phytocannabinoid fractions primarily contributed biological functionality, whereas the reinforcing fibers remained responsible for the structural performance of the composite. Such functional separation between biological and mechanical roles represents an important advantage of the present composite architecture because it allows optimization of both properties without requiring excessive concentrations of either reinforcing or bioactive components [54,55].

Equally important, cytocompatibility evaluation demonstrated that all experimental formulations maintained acceptable levels of cell viability despite the incorporation of multiple functional constituents. This observation confirms that the reinforcement strategy did not introduce measurable biological toxicity and suggests that the selected concentrations of hemp fiber, silk fibroin, and phytocannabinoid-rich fractions were compatible with the biological requirements expected for denture base biomaterials. The simultaneous preservation of cytocompatibility and antibacterial activity is particularly relevant because numerous antimicrobial modifications reported previously have been associated with reductions in mechanical performance or increased cytotoxicity [56]. In contrast, the present study demonstrates that appropriately engineered multifunctional composites may overcome these traditional compromises by balancing structural reinforcement with biological functionalization.

From a clinical perspective, the proposed composite architecture may contribute to improving the long-term performance of removable prostheses by simultaneously increasing resistance to flexural loading, accidental impact, and surface deformation while reducing bacterial colonization. Such a combination of properties may decrease the frequency of denture fracture, improve structural durability during long-term clinical service, and contribute to better oral hygiene through suppression of microbial accumulation. Although the present investigation was limited to laboratory conditions, the observed balance between mechanical reliability and biological functionality represents an encouraging step toward the development of next-generation bioactive denture base materials.

The present study possesses several strengths. First, unlike previous investigations that focused exclusively on either reinforcement or antimicrobial modification, the current work integrated both approaches within a single multifunctional composite design. Second, the comprehensive evaluation included mechanical, structural, and biological characterization, allowing direct correlation between composite architecture and material performance. Finally, the hybrid reinforcement concept provided a rational strategy for compensating the mechanical alterations associated with phytocannabinoid incorporation while preserving the desirable biological characteristics of the material.

Nevertheless, several limitations should be acknowledged. The experimental conditions were limited to an in vitro environment and therefore cannot fully reproduce the complex mechanical and chemical challenges encountered in the oral cavity. Long-term aging, thermocycling, water sorption, fatigue loading, color stability, wear behavior, and residual monomer release were not evaluated and should be investigated in future studies. Furthermore, quantitative characterization of individual phytocannabinoid concentrations was beyond the scope of the present investigation because of current regulatory limitations. Future investigations should additionally explore different reinforcement ratios, long-term clinical aging protocols, and in vivo performance to optimize the multifunctional composite architecture proposed in this study. A limitation of this study is the use of BEAS-2B bronchial epithelial cells, which, although suitable for preliminary cytotoxicity screening, do not fully represent the oral cellular environment; therefore, future studies using oral-derived cell models are warranted.

Overall, the present findings demonstrate that hybrid reinforcement using hemp fiber and silk fibroin effectively compensated for the mechanical alterations associated with phytocannabinoid functionalization while preserving the chemical stability and biological compatibility of the PMMA matrix. Rather than functioning as independent modifications, the reinforcing fibers and phytocannabinoid-rich fractions acted through complementary mechanisms that collectively generated a mechanically balanced, structurally stable, and biologically functional denture base composite. This multifunctional design strategy may therefore provide a promising foundation for the future development of advanced polymeric biomaterials intended for long-term prosthodontic applications.

## 5. Conclusions

Within the limitations of this in vitro study, the material system and phytocannabinoid condition significantly influenced the mechanical performance of PMMA denture base resin. PMMA/HF/SF exhibited the most favorable flexural strength, elastic modulus, surface hardness, and impact strength across the tested conditions, supporting a beneficial complementary effect of hybrid hemp fiber/silk fibroin reinforcement. Significant material system × phytocannabinoid condition interactions for flexural strength, hardness, and impact strength showed that the magnitude of the phytocannabinoid-associated mechanical changes depended on the material system, whereas elastic modulus showed independent main effects without interaction. Although phytocannabinoid-rich fractions produced progressive reductions in several mechanical parameters, the hybrid-reinforced composites maintained superior performance compared with unmodified PMMA. The present design does not independently test HF × SF synergy; therefore, the hybrid effect is interpreted as complementary rather than as formal statistical evidence of synergism.

The phytocannabinoid fractions provided a clear biological advantage by reducing the viability and biofilm formation of *S.mutans* and *L. acidophilus*. ATR–FTIR and XRD analyses confirmed that the fundamental chemical structure and predominantly amorphous architecture of PMMA were preserved after reinforcement and phytocannabinoid incorporation. In addition, LDH assay results demonstrated low cytotoxicity across all experimental groups, supporting the cytocompatibility of the developed formulations.

Overall, within the limitations of this initial in vitro study, the PMMA/HF/SF–phytocannabinoid formulations demonstrated promising mechanical and antibacterial properties. However, these findings should not be interpreted as evidence of clinical suitability, and further investigations incorporating simulated oral aging, fatigue testing, oral-specific biological models, and ultimately clinical evaluation are required before their potential use as denture base materials can be established.

## Figures and Tables

**Figure 1:**
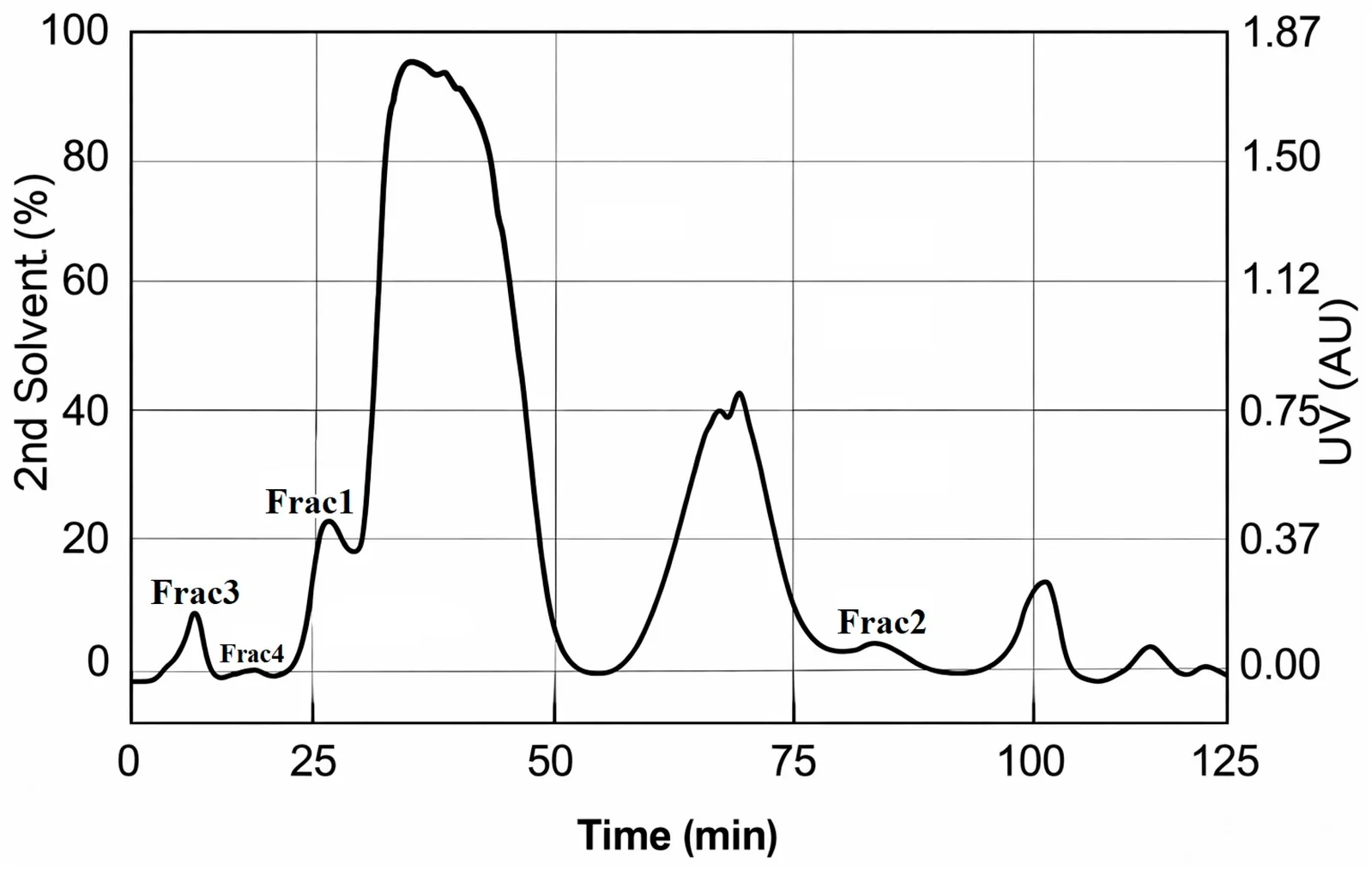
Flash chromatograms of fractions (Frac1, Frac2, Frac3, and Frac4).

**Figure 2:**
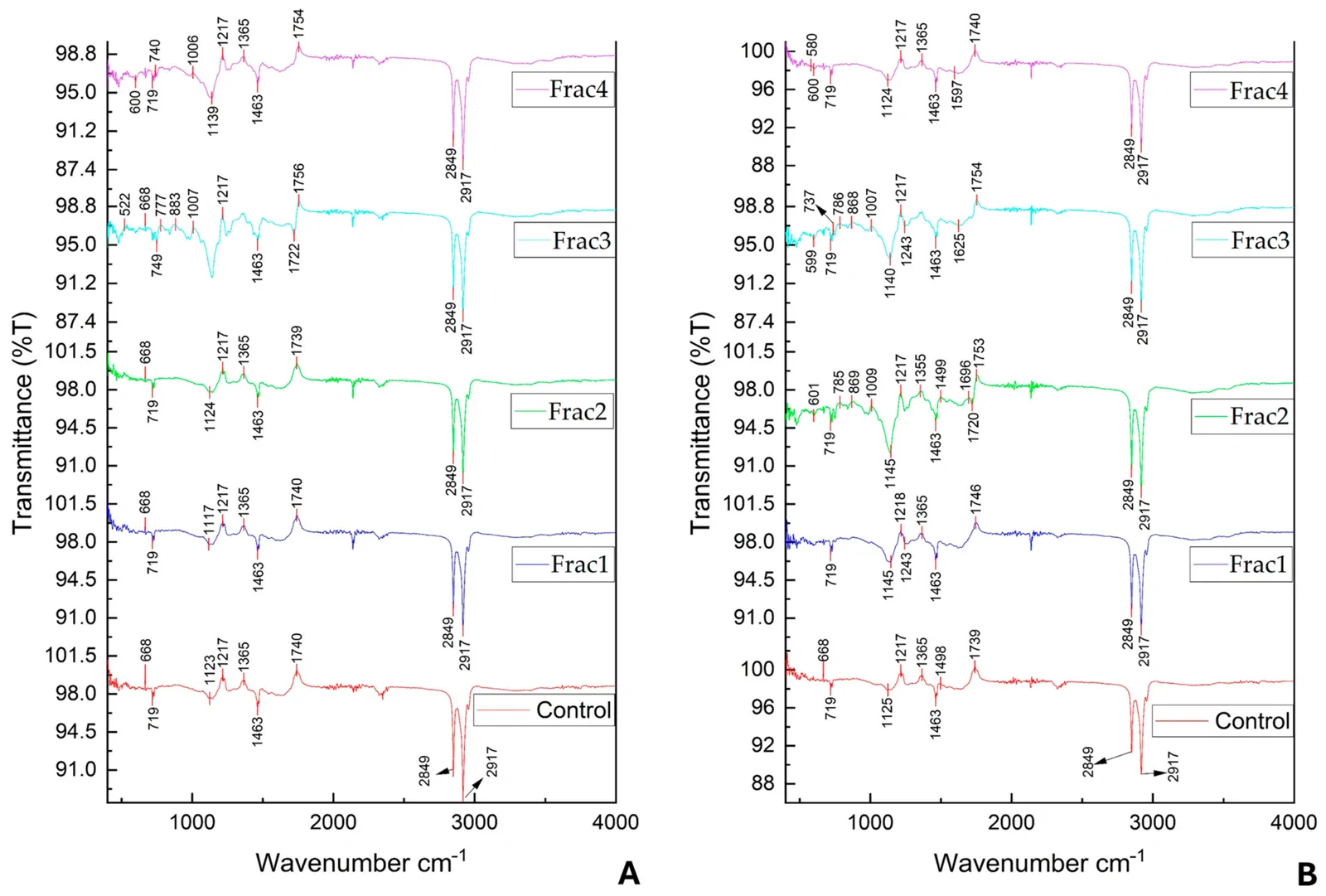
(**A**) FTIR/ATR spectra for PMMA/HF/SF. (**B**) FTIR/ATR spectra for PMMA/SF. Cannabinoid fractions: Control, Frac1, Frac2, Frac3, and Frac4.

**Figure 3:**
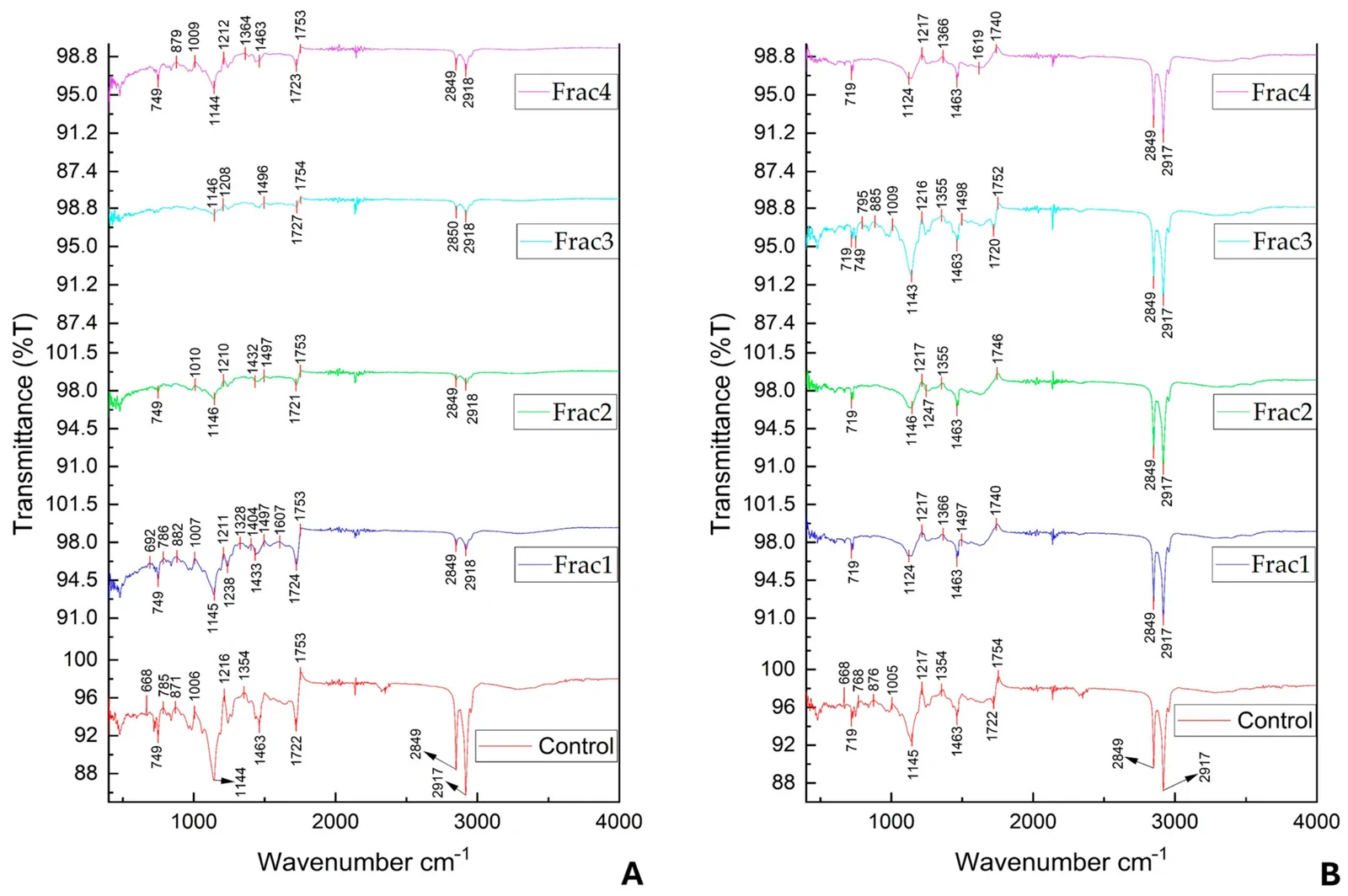
(**A**) FTIR/ATR spectra for PMMA/HF. (**B**) FTIR/ATR spectra for pure PMMA. Cannabinoid fractions: Control, Frac1, Frac2, Frac3, and Frac4.

**Figure 4:**
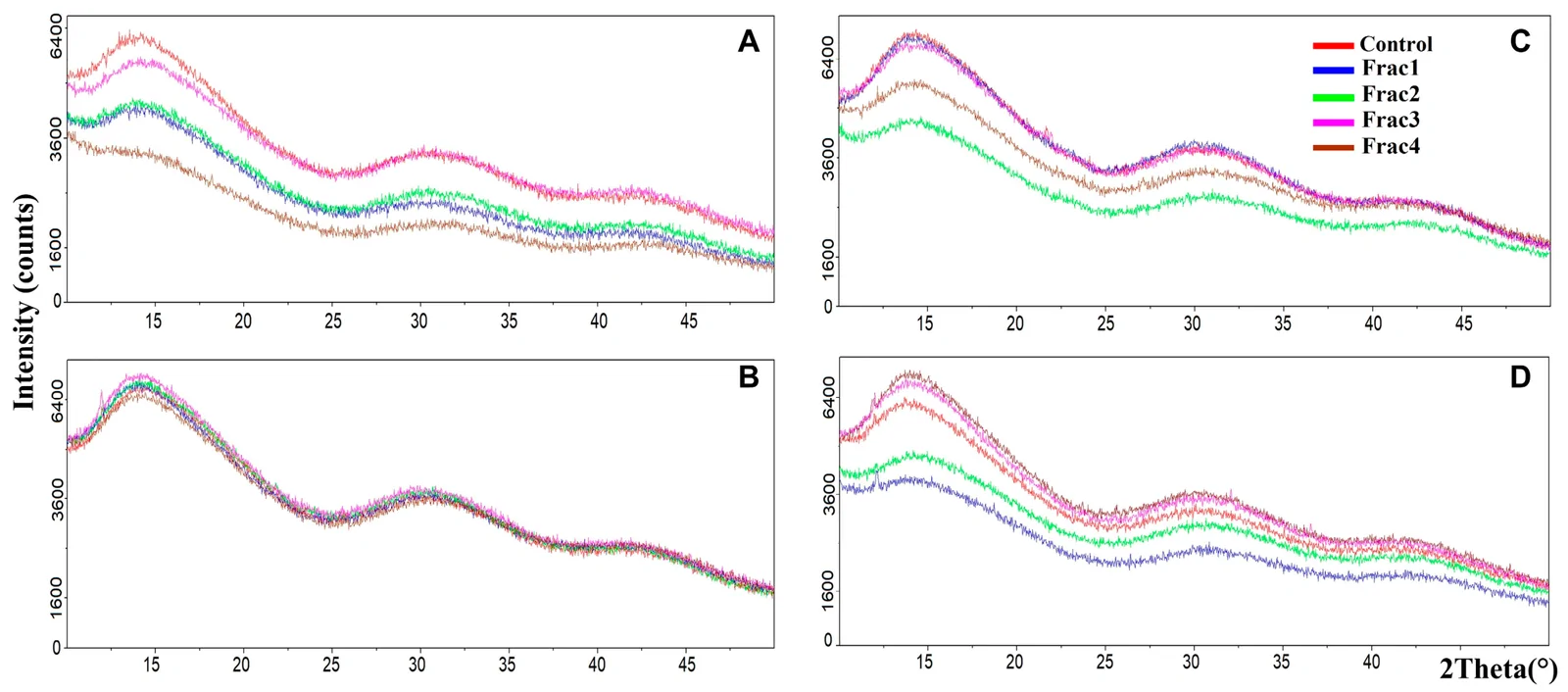
Typical X-ray diffraction pattern for pure and cannabinoid-fractionated (Frac1, Frac2, Frac3, and Frac4) PMMA/HF/SF (**A**), PMMA/SF (**B**), PMMA/HF (**C**), and PMMA (**D**).

**Figure 5:**
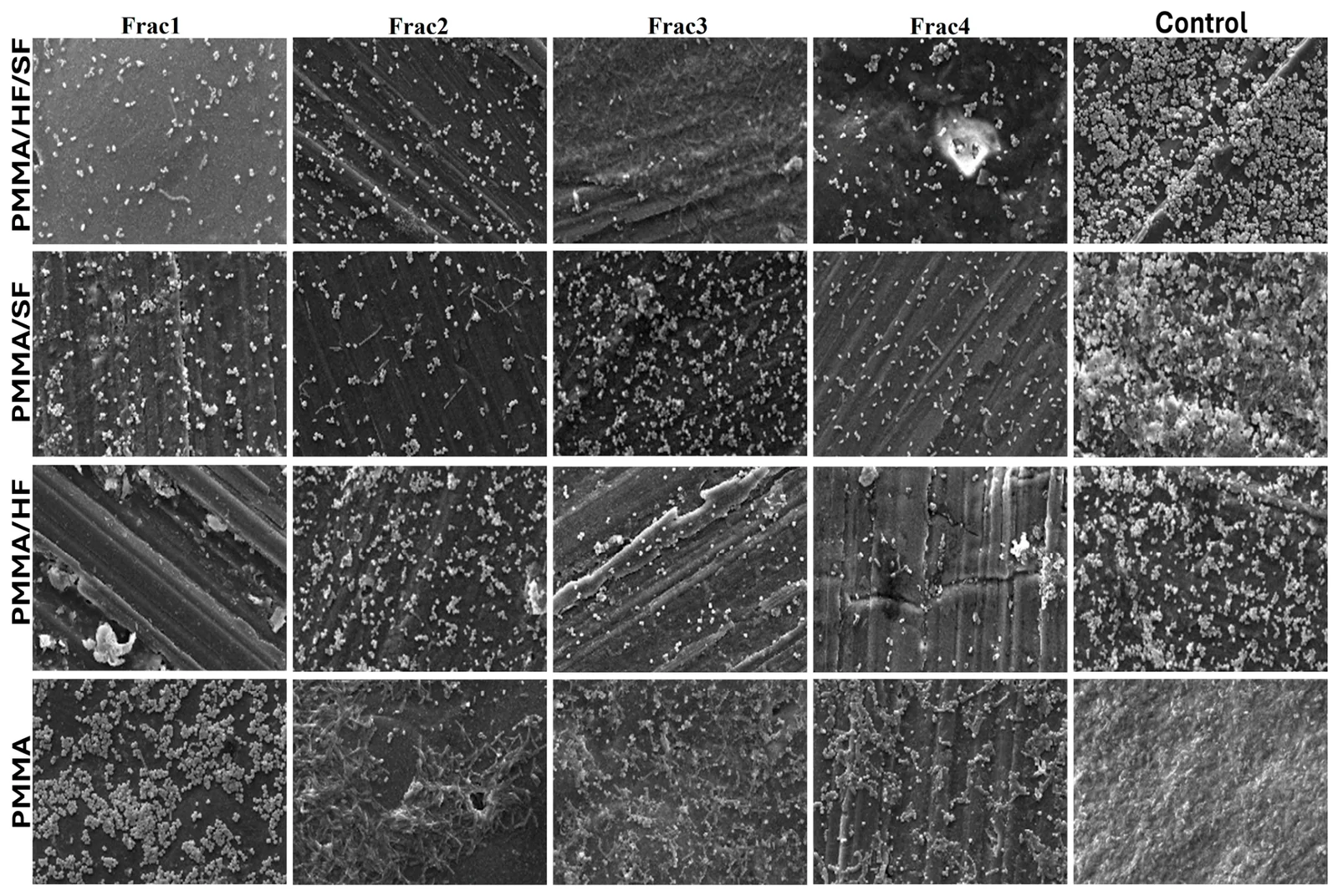
A scanning electron microscopic image (×25 K magnification) showing *L. acidophilus* biofilm formations on materials.

**Figure 6:**
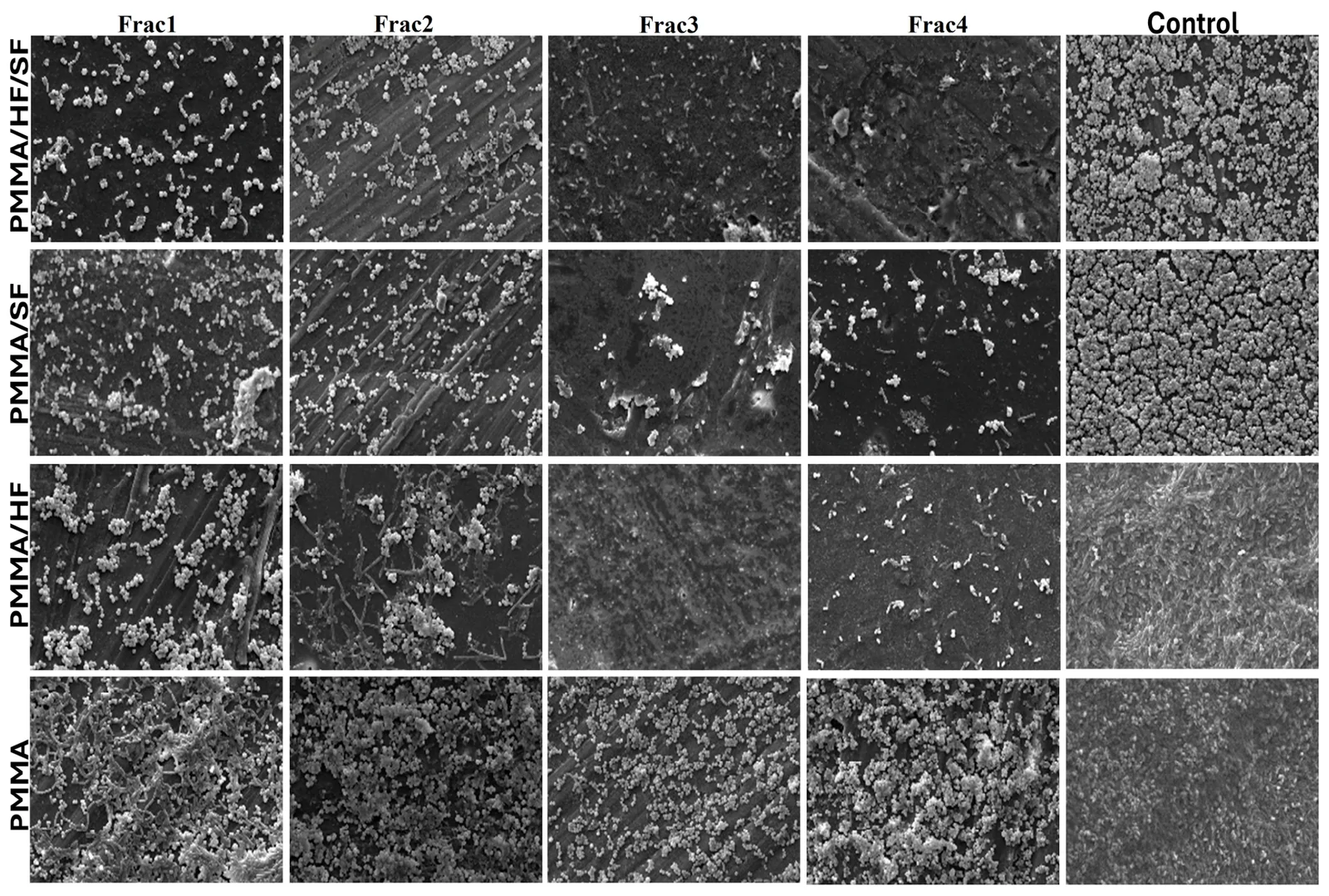
A scanning electron microscopic image (×25 K magnification) showing *S. mutans* biofilm formations on materials.

**Table 1:** Composition, source, and functional role of the materials used in the experimental PMMA composite formulations.

Material	Manufacturer	Country	Composition	Function in Composite
Heat-cured PMMA	Meliodent^®^ Kulzer GmbH	Germany	Poly(methyl methacrylate) polymer powder and methyl methacrylate monomer	Polymer matrix
Hemp fiber	Prepared *in-house* from hemp stalks supplied by the Hemp Research Institute, Yozgat Bozok University, Türkiye	Türkiye	Cellulose-rich natural fiber	Primary mechanical reinforcement
Silk fibroin	Extracted *in-house* from *Bombyx mori* cocoons obtained from KOZABİRLİK Agricultural Cooperative, Bursa, Türkiye	Türkiye	Fibrous protein	Toughness enhancement & interface improvement
Phytocannabinoid Fraction Frac1, Frac2, Frac3, and Frac4	Prepared *in-house* from *Cannabis sativa* L. supplied by the Hemp Research Institute, Yozgat Bozok University, Türkiye	Türkiye	Bioactive phytochemical fraction	Biological functionality

**Table 2:** Comparative GC–MS composition of the Frac1–Frac4 with retention time and relative abundance.

Compound	RT (min)	Frac1 (%)	Frac2 (%)	Frac3 (%)	Frac4 (%)
Cannabidiol	93.508; 98.773	21.20	19.18	–	–
Cannabichromene	72.892; 93.651; 93.150; 93.005	0.12	36.14	4.83	0.38
DELTA,8-Tetrahydrocannabinol	86.793; 96.230	–	17.28; 5.66	–	–
Dronabinol	102.392	8.10	–	–	–
11-Hydroxy-DELTA,9-tetrahydrocannabinol	96.803	–	2.82	–	–
3-PROPYL-CANNABIDIOL	84.994; 84.431	61.41	–	–	12.87
Hexahydrocannabinol	97.358	0.57	–	–	–
Cannabispiradienone	86.693	–	–	2.86	–
Cannabispiran	90.856	–	–	8.15	–
Caryophyllene oxide	65.625; 60.460; 68.638	0.23	–	3.63	52.99
(-)-Caryophyllene oxide	54.317	1.11	–	–	–
trans-Z-.alpha.-Bisabolene epoxide	54.083; 58.394; 66.341	1.35; 0.14	–	8.85	–
cis-Z-.alpha.-Bisabolene epoxide	66.888	–	–	–	0.56
Guaiol	63.475	–	–	–	3.42
Veridiflorol	62.766	0.99	–	–	–
trans-Sesquisabinene hydrate	56.418	–	–	3.20	–
Illudol	64.083	–	–	8.68	–
1,3-Benzenediol, 5-pentyl-	63.265	–	–	49.98	–
1,3-Benzenediol, 2-(3,7-dimethyl-2,6-octadienyl)-5-pentyl-	99.222; 90.767	2.06	0.47	–	–
Farnesene epoxide, E-	55.275	0.21	–	–	–
8-ACETOXYLINALOOL	66.963	1.01	–	–	–
4,4,8-Trimethyltricyclo[6.3.1.0(1,5)]dodecane-2,9-diol	67.212; 65.747	0.62	–	–	1.52
2-Cyclopentene-1-carboxylic acid, 1,3-dimethyl-2-ethyl-, ethyl ester	95.455	–	11.83	–	–
1,3-Benzenedicarboxylic acid, bis(2-ethylhexyl) ester	105.125	–	6.35	–	–
Caryophyllene oxide-related/other GC–MS identified constituents	—	—	—	—	—

**Table 3:** Composition of the experimental groups and reinforcement formulations.

Group Code	Material System	Hemp Fiber (wt%)	Silk Fibroin (wt%)	Phytocannabinoid Fraction	Phytocannabinoid Content (wt%)
PMMA-Control	PMMA	0	0	None	0
PMMA-Frac1	PMMA	0	0	Frac1	1.0
PMMA-Frac2	PMMA	0	0	Frac2	1.0
PMMA-Frac3	PMMA	0	0	Frac3	1.0
PMMA-Frac4	PMMA	0	0	Frac4	1.0
PMMA/HF-Control	PMMA + HF	1.0	0	None	0
PMMA/HF-Frac1	PMMA + HF	1.0	0	Frac1	1.0
PMMA/HF-Frac2	PMMA + HF	1.0	0	Frac2	1.0
PMMA/HF-Frac3	PMMA + HF	1.0	0	Frac3	1.0
PMMA/HF-Frac4	PMMA + HF	1.0	0	Frac4	1.0
PMMA/SF-Control	PMMA + SF	0	1.0	None	0
PMMA/SF-Frac1	PMMA + SF	0	1.0	Frac1	1.0
PMMA/SF-Frac2	PMMA + SF	0	1.0	Frac2	1.0
PMMA/SF-Frac3	PMMA + SF	0	1.0	Frac3	1.0
PMMA/SF-Frac4	PMMA + SF	0	1.0	Frac4	1.0
PMMA/HF/SF-Control	PMMA + HF/SF	0.5	0.5	None	0
PMMA/HF/SF-Frac1	PMMA + HF/SF	0.5	0.5	Frac1	1.0
PMMA/HF/SF-Frac2	PMMA + HF/SF	0.5	0.5	Frac2	1.0
PMMA/HF/SF-Frac3	PMMA + HF/SF	0.5	0.5	Frac3	1.0
PMMA/HF/SF-Frac4	PMMA + HF/SF	0.5	0.5	Frac4	1.0

**Table 4:** Mechanical properties of experimental groups and two-way factorial ANOVA results (Mean ± SD, n = 10) *.

Group	Flexural Strength (MPa)	Elastic Modulus (GPa)	Hardness (VHN)	Impact Strength (kJ/m^2^)
PMMA Control	94.59 ± 2.46 Da	2.366 ± 0.019	19.32 ± 0.21 Da	15.05 ± 0.21 Da
PMMA Frac1	92.92 ± 0.73 Da	2.317 ± 0.019	18.51 ± 0.22 Db	14.20 ± 0.21 Db
PMMA Frac2	90.02 ± 0.75 Db	2.247 ± 0.019	17.91 ± 0.24 Dc	13.44 ± 0.21 Dc
PMMA Frac3	86.76 ± 0.72 Dc	2.167 ± 0.019	17.20 ± 0.23 Dd	12.60 ± 0.21 Dd
PMMA Frac4	83.31 ± 0.85 Dd	2.086 ± 0.021	16.54 ± 0.23 De	11.82 ± 0.20 De
PMMA/SF Control	111.02 ± 2.88 Ca	2.436 ± 0.019	21.86 ± 0.22 Ca	18.02 ± 0.22 Ca
PMMA/SF Frac1	109.21 ± 2.79 Ca	2.386 ± 0.019	20.94 ± 0.22 Cb	17.12 ± 0.22 Cb
PMMA/SF Frac2	105.87 ± 2.70 Cb	2.316 ± 0.019	20.22 ± 0.22 Cc	16.22 ± 0.22 Cc
PMMA/SF Frac3	102.10 ± 2.61 Cc	2.236 ± 0.019	19.44 ± 0.22 Cd	15.28 ± 0.22 Cd
PMMA/SF Frac4	98.30 ± 2.45 Cd	2.156 ± 0.019	18.72 ± 0.22 Ce	14.36 ± 0.21 Ce
PMMA/HF Control	128.80 ± 3.35 Ba	2.356 ± 0.019	23.96 ± 0.22 Ba	21.15 ± 0.22 Ba
PMMA/HF Frac1	126.69 ± 0.99 Ba	2.307 ± 0.019	23.04 ± 0.22 Bb	20.21 ± 0.22 Bb
PMMA/HF Frac2	122.71 ± 0.99 Bb	2.237 ± 0.019	22.18 ± 0.22 Bc	19.12 ± 0.22 Bc
PMMA/HF Frac3	118.29 ± 0.97 Bc	2.157 ± 0.019	21.22 ± 0.22 Bd	18.00 ± 0.22 Bd
PMMA/HF Frac4	113.47 ± 1.16 Bd	2.076 ± 0.021	20.28 ± 0.22 Be	16.92 ± 0.22 Be
PMMA/HF/SF Control	138.04 ± 2.05 Aa	2.519 ± 0.017	26.14 ± 0.22 Aa	24.22 ± 0.22 Aa
PMMA/HF/SF Frac1	134.92 ± 1.80 Ab	2.471 ± 0.018	25.26 ± 0.22 Ab	23.14 ± 0.22 Ab
PMMA/HF/SF Frac2	130.63 ± 1.78 Ac	2.401 ± 0.018	24.32 ± 0.22 Ac	22.02 ± 0.22 Ac
PMMA/HF/SF Frac3	126.00 ± 1.68 Ad	2.321 ± 0.018	23.28 ± 0.22 Ad	20.88 ± 0.22 Ad
PMMA/HF/SF Frac4	120.93 ± 1.66 Ae	2.241 ± 0.018	22.22 ± 0.22 Ae	19.72 ± 0.22 Ae
Material system	F(3,180) = 4277.137; *p* = 5.173 × 10^−167^; η*p*^2^ = 0.986	F(3,180) = 810.121; *p* = 3.073 × 10^−104^; η*p*^2^ = 0.931	F(3,180) = 7369.351; *p* = 4.761 × 10^−188^; η*p*^2^ = 0.992	F(3,180) = 14,511.601; *p* = 2.231 × 10^−214^; η*p*^2^ = 0.996
Phytocannabinoid condition	F(4,180) = 342.180; *p* = 6.068 × 10^−83^; η*p*^2^ = 0.884	F(4,180) = 1428.581; *p* = 3.812 × 10^−135^; η*p*^2^ = 0.969	F(4,180) = 1426.583; *p* = 4.307 × 10^−135^; η*p*^2^ = 0.969	F(4,180) = 2036.961; *p* = 1.192 × 10^−148^; η*p*^2^ = 0.978
System × Condition	F(12,180) = 2.778; *p* = 0.001700; η*p*^2^ = 0.156	F(12,180) = 0.006; *p* = 1.000000; η*p*^2^ < 0.001	F(12,180) = 9.480; *p* = 3.956 × 10^−14^; η*p*^2^ = 0.387	F(12,180) = 11.417; *p* = 7.123 × 10^−17^; η*p*^2^ = 0.432
Post-hoc interpretation	Simple effects + Tukey	Main effects (no interaction)	Simple effects + Tukey	Simple effects + Tukey

* Data are presented as mean ± SD (n = 10 per experimental cell). Mechanical outcomes were analyzed using two-way factorial ANOVA. For flexural strength, Vickers hardness, and impact strength, uppercase letters compare material systems within the same phytocannabinoid condition and lowercase letters compare phytocannabinoid conditions within the same material system using Tukey-adjusted simple-effects comparisons; values sharing the same letter within the relevant comparison family are not significantly different (*p* ≥ 0.05). Elastic modulus showed no significant interaction and is therefore presented without cell-wise lettering; main effects and Tukey-adjusted marginal comparisons are described in the text. Detailed post-hoc results are provided in Supplementary File S2.

**Table 5:** MTT-derived relative bacterial viability (%) of the experimental groups (mean ± SD, n = 3 independent biological experiments) *.

Material System	*L. acidophilus* Frac1	Frac2	Frac3	Frac4	Control	*S. mutans* Frac1	Frac2	Frac3	Frac4	Control
PMMA/HF/SF	14.59 ± 0.39 Aa	15.22 ± 0.26 Aa	18.31 ± 0.32 Ab	19.40 ± 0.49 Ab	85.44 ± 0.66 Ac	24.13 ± 0.45 Ad	22.57 ± 0.44 Ac	16.95 ± 0.39 Ab	15.21 ± 0.39 Aa	84.39 ± 0.45 Ae
PMMA/SF	15.28 ± 0.23 Aa	22.29 ± 0.33 Bb	35.44 ± 0.37 Bc	22.47 ± 0.39 Bb	89.42 ± 0.83 Bd	27.94 ± 0.44 Bd	24.76 ± 0.46 Bc	19.24 ± 0.45 Bb	17.18 ± 0.43 Ba	92.59 ± 0.45 Be
PMMA/HF	17.64 ± 0.26 Ba	26.31 ± 0.43 Cb	37.46 ± 0.42 Cd	27.50 ± 0.37 Cc	91.52 ± 0.44 Ce	28.69 ± 0.42 Bb	28.10 ± 0.36 Cb	20.51 ± 0.41 Ba	21.19 ± 0.45 Ca	92.77 ± 0.42 Bc
PMMA	35.99 ± 0.76 Cc	30.86 ± 0.53 Da	39.05 ± 0.78 Dd	32.29 ± 0.51 Db	96.63 ± 0.40 De	44.94 ± 0.47 Cc	44.64 ± 0.44 Dc	29.67 ± 0.45 Ca	32.71 ± 0.41 Db	98.13 ± 0.45 Cd

* Data are presented as mean ± SD from three independent biological experiments (n = 3 per experimental cell). For each bacterial species, uppercase letters compare material systems within the same phytocannabinoid condition, whereas lowercase letters compare phytocannabinoid conditions within the same material system using Tukey-adjusted simple-effects comparisons following a significant material system × phytocannabinoid condition interaction. Values sharing the same letter within the relevant comparison family are not significantly different (adjusted *p* ≥ 0.05). MTT values represent relative bacterial metabolic viability and should not be interpreted as a direct measure of bacterial killing or total biofilm biomass.

**Table 6:** Two-way factorial ANOVA of MTT-derived bacterial viability *.

Bacterial Species	Effect	df	F	Exact *p*	Partial η^2^
*L. acidophilus*	Material system	3, 40	2904.299	8.095 × 10^−47^	0.995
	Phytocannabinoid condition	4, 40	43,495.634	3.558 × 10^−72^	>0.999
	Material system × condition	12, 40	194.671	1.638 × 10^−31^	0.983
*S. mutans*	Material system	3, 40	2243.032	1.381 × 10^−44^	0.994
	Phytocannabinoid condition	4, 40	28,277.512	1.951 × 10^−68^	>0.999
	Material system × condition	12, 40	56.598	3.249 × 10^−21^	0.944

* For each microorganism, MTT-derived viability was analyzed separately using a 4-material-system × 5-phytocannabinoid-condition two-way factorial ANOVA (n = 3 independent biological experiments per cell). Because the material system × phytocannabinoid condition interaction was significant for both bacterial species, significant omnibus effects were followed by simple-effects analyses and Tukey-adjusted pairwise comparisons. Exact *p*-values and partial η^2^ are reported.

**Table 7:** LDH-derived cytotoxicity (%) of the experimental formulations (mean ± SD, n = 3 independent biological experiments) *.

Material System	Frac1	Frac2	Frac3	Frac4	Control
PMMA/HF/SF	2.57 ± 0.23 Abc	3.80 ± 0.70 Bac	4.57 ± 0.55 Aa	4.27 ± 0.56 Aa	1.31 ± 0.18 Bb
PMMA/SF	3.01 ± 0.29 Ab	3.75 ± 0.63 Bab	4.50 ± 0.62 Aa	4.52 ± 0.45 Aa	2.51 ± 0.21 Ab
PMMA/HF	2.68 ± 0.21 Abc	2.28 ± 0.32 Abc	3.03 ± 0.77 Ac	4.73 ± 0.44 Aa	1.52 ± 0.29 Bb
PMMA	3.28 ± 0.56 Aab	2.74 ± 0.28 ABb	4.57 ± 0.81 Aa	3.99 ± 0.56 Aab	2.85 ± 0.27 Ab

* Values are presented as mean ± SD from three independent biological experiments (n = 3 per experimental cell). Uppercase letters compare material systems within the same phytocannabinoid condition; lowercase letters compare phytocannabinoid conditions within the same material system. Values sharing at least one letter within the relevant comparison family are not significantly different according to Tukey-adjusted simple-effects comparisons (adjusted *p* ≥ 0.05). Comparisons were performed following the significant material system × phytocannabinoid condition interaction detected by two-way factorial ANOVA.

## Data Availability

The data presented in this study are available from the corresponding author upon reasonable request.

## Supplementary Materials

The following supporting information can be downloaded at: https://www.mdpi.com/article/10.3390/polym18192361/s1, Supplementary File S1: GC–MS chromatograms and corresponding peak data for the phytocannabinoid-rich fractions (Frac1–Frac4); Supplementary File S2: Detailed statistical analyses of the mechanical properties, including assumption diagnostics, two-way factorial ANOVA, simple-effects analyses, Tukey-adjusted multiple comparisons, and supporting statistical outputs; Supplementary File S3: Detailed statistical analyses of MTT-derived bacterial metabolic viability for *L. acidophilus* and *S. mutans*, including assumption diagnostics, factorial ANOVA, simple-effects analyses, and Tukey-adjusted multiple comparisons; Supplementary File S4: Detailed statistical analyses of LDH-derived cytotoxicity, including assumption diagnostics, factorial ANOVA, simple-effects analyses, and Tukey-adjusted multiple comparisons; Supplementary File S5: Interpretation note.

## Abbreviations

**Abbreviation**	**Definition**
ANOVA	Analysis of Variance
ATR	Attenuated Total Reflectance
CBC	Cannabichromene
CBD	Cannabidiol
CO_2_	Carbon Dioxide
DPBS	Dulbecco’s Phosphate-Buffered Saline
FTIR	Fourier Transform Infrared Spectroscopy
FS	Flexural Strength
GC–MS	Gas Chromatography–Mass Spectrometry
HF	Hemp Fiber
LDH	Lactate Dehydrogenase
MMA	Methyl Methacrylate
MTT	3-(4,5-Dimethylthiazol-2-yl)-2,5-diphenyltetrazolium Bromide Assay
PMMA	Poly(methyl methacrylate)
RT	Room Temperature
SD	Standard Deviation
SEM	Scanning Electron Microscopy
SF	Silk Fibroin
THC	Δ9-Tetrahydrocannabinol
UV	Ultraviolet
VHN	Vickers Hardness Number
XRD	X-ray Diffraction
