# Supplementary material for: Hybrid-Reinforced Phytocannabinoid-Functionalized PMMA Denture Base Composites: An In Vitro Study
Source: Polymers (Basel). 2026 Sep 28;18(19):2361. doi: 10.3390/polym18192361 (PMC13667271; doi:10.3390/polym18192361)
Supplement: Supplementary file 1 [file polymers-18-02361-s001.zip › polymers-4441837-supplementary.pdf]

Supplementary File

GC–MS chromatograms and detailed statistical analyses for mechanical, MTT, and LDH outcomes

S1. The TIC patterns for all four Frac1-4 samples

Fraction 1 (Frac1)

Peak#	Ret.Time	Start Tm	End Tm	m/z	Area	Area%	Height	Height%	A/H	Mark	Name
1	54.083	53.785	54.155	TIC	122182481	1.35	9543025	5.31	12.80		trans-Z-.alpha.-Bisabolene epoxide
2	54.317	54.155	54.420	TIC	99851957	1.11	13718782	7.63	7.28	V	(-)-Caryophyllene oxide
3	55.275	55.130	55.360	TIC	18959431	0.21	2904549	1.62	6.53		Farnesene epoxide, E-
4	58.394	58.290	58.580	TIC	12493094	0.14	1343919	0.75	9.30		trans-Z-.alpha.-Bisabolene epoxide
5	62.139	62.010	62.330	TIC	43074269	0.48	4972403	2.77	8.66	V	Cholestane, 4,5-epoxy-, (4.alpha.,5.alpha.)-
6	62.766	62.620	62.950	TIC	89202541	0.99	8982085	4.99	9.93	V	Veridiflorol
7	64.072	63.960	64.240	TIC	19613437	0.22	2664082	1.48	7.36	V	Cyclohexanemethanol, 4-ethenyl-.alpha.,.alpha.,4-trimethyl-3-(1-met
8	65.322	65.210	65.430	TIC	16087417	0.18	2959670	1.65	5.44		4-Isopropenyl-4,7-dimethyl-1-oxaspiro[2.5]octane
9	65.625	65.450	65.760	TIC	21015609	0.23	2485036	1.38	8.46		Caryophyllene oxide
10	66.963	66.620	67.060	TIC	91262435	1.01	8549936	4.75	10.67		8-ACETOXYLINALOOL
11	67.212	67.060	67.560	TIC	55872959	0.62	5600846	3.11	9.98	V	4,4,8-Trimethyltricyclo[6.3.1.0(1,5)]dodecane-2,9-diol
12	72.892	72.685	73.085	TIC	10882851	0.12	1664122	0.93	6.54		Cannabichromene
13	84.994	83.435	87.060	TIC	5549309669	61.41	37353145	20.76	148.56		3-PROPYL-CANNABIDIOL
14	93.508	92.510	94.410	TIC	1915704834	21.20	33452146	18.60	57.27	V	Cannabidiol
15	97.358	97.060	97.560	TIC	51510295	0.57	3483435	1.94	14.79	V	Hexahydrocannabinol
16	99.222	98.960	99.560	TIC	186583336	2.06	14439579	8.03	12.92	V	1,3-Benzenediol, 2-(3,7-dimethyl-2,6-octadienyl)-5-pentyl-
17	102.392	101.660	102.710	TIC	732185313	8.10	25713662	14.30	28.47	V	Dronabinol

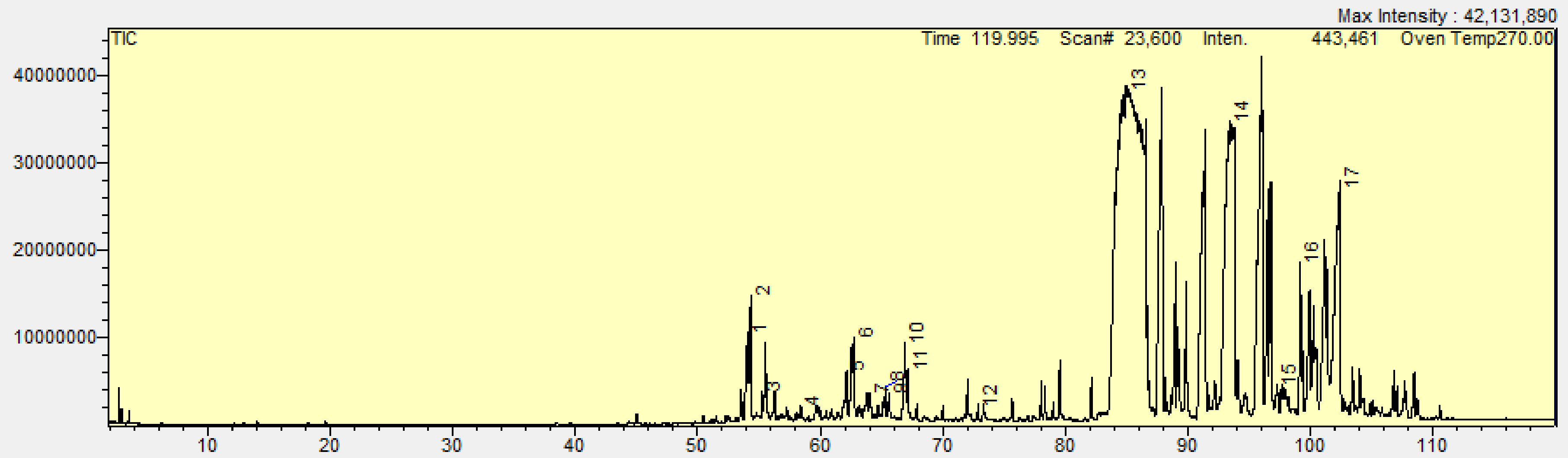

Fraction 2 (Frac2)

Peak#	Ret.Time	Start Tm	End Tm	m/z	Area	Area%	Height	Height%	A/H	Mark	Name
1	86.793	85.870	86.970	TIC	1262956622	17.28	34542433	16.43	36.56	MI	.DELTA.8-Tetrahydrocannabinol
2	90.767	90.650	91.100	TIC	34428827	0.47	4191148	1.99	8.21	V	1,3-Benzenediol, 2-(3,7-dimethyl-2,6-octadienyl)-5-pentyl-
3	91.294	91.100	91.550	TIC	19402328	0.27	1980751	0.94	9.80	V	1H-4-Oxabenzo(f)cyclobut(cd)inden-8-ol, 1a-.alpha.,2,3,3a,8b-.alpha.,8c-.alpha.-hexahydro-1,1,3a-trimethyl-6-pentyl-
4	93.651	92.650	94.875	TIC	2640453312	36.14	26289041	12.51	100.44	V	Cannabichromene
5	95.455	94.875	95.675	TIC	864108755	11.83	33341355	15.86	25.92	V	2-Cyclopentene-1-carboxylic acid, 1,3-dimethyl-2-ethyl-, ethyl ester
6	96.230	95.675	96.575	TIC	413454836	5.66	10755643	5.12	38.44	V	.DELTA.8-Tetrahydrocannabinol
7	96.803	96.575	96.925	TIC	206219705	2.82	16409889	7.81	12.57	V	11-Hydroxy-.DELTA.9-tetrahydrocannabinol
8	98.773	97.975	99.025	TIC	1401558686	19.18	43836708	20.86	31.97	V	Cannabidiol
9	105.125	104.725	105.275	TIC	464200939	6.35	38839658	18.48	11.95	V	1,3-Benzenedicarboxylic acid, bis(2-ethylhexyl) ester

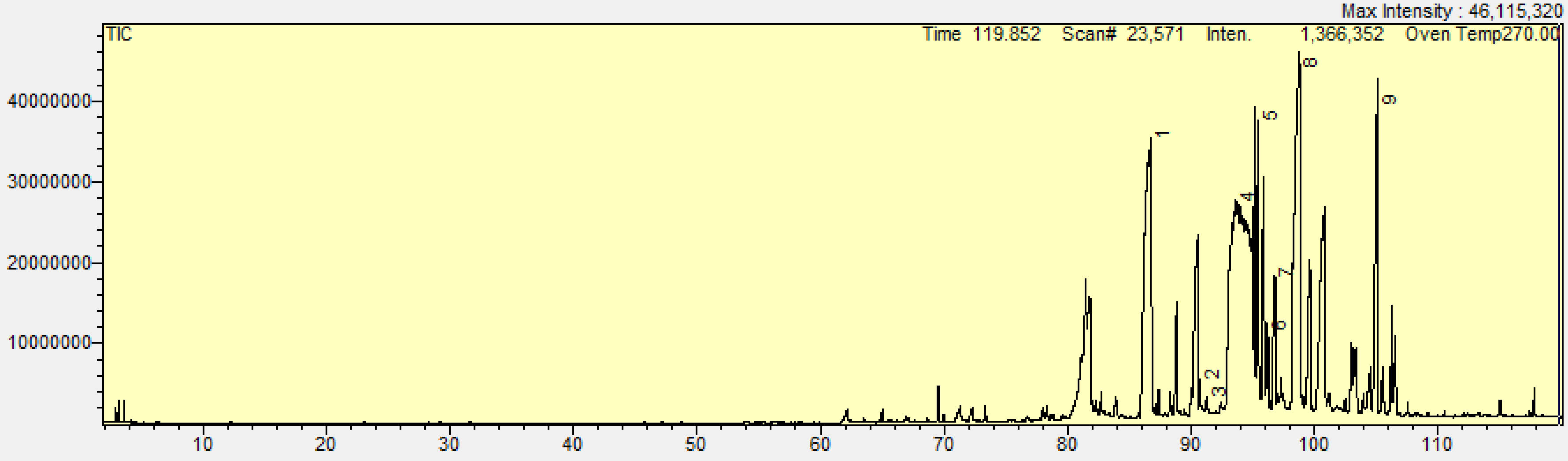

Fraction 3 (Frac3)

Print Edit View Similarity Search

Peak#	Ret.Time	Start Tm	End Tm	m/z	Area	Area%	Height	Height%	A/H	Mark	Name
1	35.704	35.550	35.830	TIC	21914923	0.79	2766146	2.59	7.92		1,2-Cyclohexanediol, 1-methyl-4-(1-methylethenyl)-
2	55.713	55.410	55.770	TIC	52015965	1.87	5208658	4.87	9.99		2-Cyclohexen-1-one, 4-(3-hydroxy-1-butenyl)-3,5,5-trimethyl-
3	56.418	56.110	56.600	TIC	89221321	3.20	6601528	6.17	13.52		trans-Sesquisabinene hydrate
4	60.460	60.380	60.540	TIC	101106323	3.63	15124117	14.14	6.69	V	Caryophyllene oxide
5	63.265	62.125	63.920	TIC	1392020256	49.98	21795735	20.39	63.87	MI	1,3-Benzenediol, 5-pentyl-
6	64.083	63.950	64.525	TIC	241811809	8.68	11624008	10.87	20.80	V	Illudol
7	65.503	64.875	65.600	TIC	172145651	6.18	3693465	3.45	46.61	V	1H-2-Benzopyran-1-one, 3,4-dihydro-8-hydroxy-6-methoxy-3-methyl-, (R)-
8	66.341	65.825	66.450	TIC	246512134	8.85	11837506	11.07	20.82	V	trans-Z-.alpha.-Bisabolene epoxide
9	86.693	86.250	87.100	TIC	79684336	2.86	2682339	2.51	29.71	V	Cannabispiradienone
10	90.856	89.425	90.975	TIC	226980221	8.15	11055007	10.34	20.53	V	Cannabispiran
11	93.150	92.725	93.525	TIC	134474118	4.83	13777639	12.88	9.76		Cannabichromene
12	96.675	96.225	97.575	TIC	27355907	0.98	770929	0.72	35.48	V	Phenol, 2-methoxy-4-propyl-

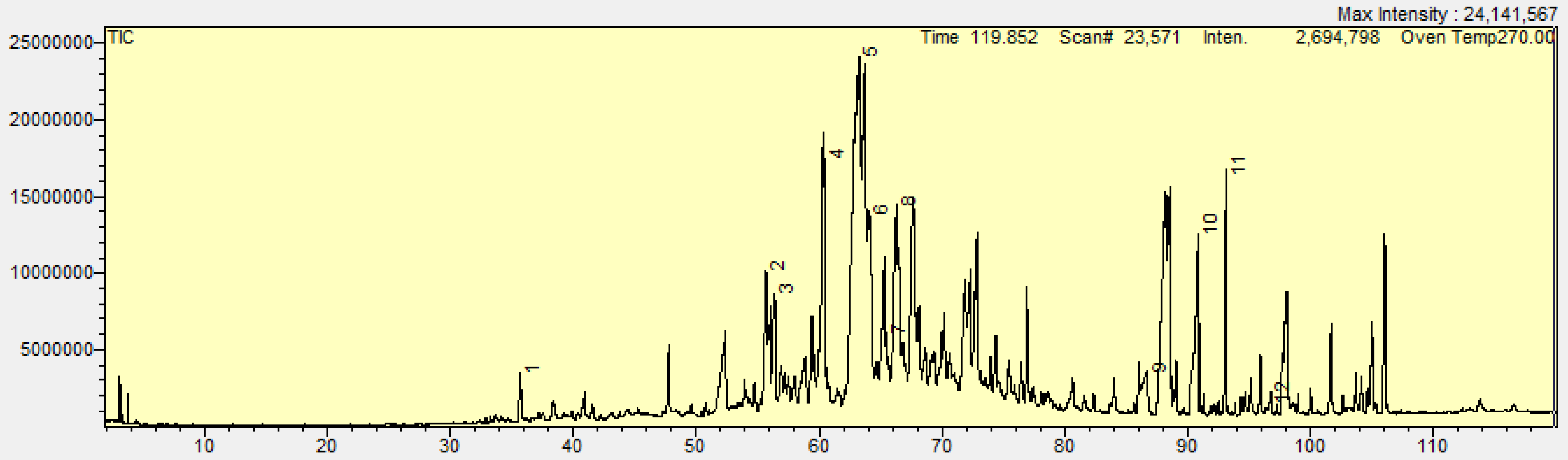

Fraction 4 (Frac4)

Peak#	Ret.Time	Start Tm	End Tm	m/z	Area	Area%	Height	Height%	A/H	Mark	Name
1	63.475	63.425	63.555	TIC	190491346	3.42	36268897	15.21	5.25	V	Guaiol
2	63.934	63.665	64.025	TIC	300200591	5.39	20892607	8.76	14.37	V	Cyclohexanemethanol, 4-ethenyl-.alpha.,.alpha.,4-trimethyl-3-(1-methylethenyl)-, [1R-(1.alpha.,3.alpha.,4.beta.)]-
3	64.158	64.025	64.205	TIC	167761170	3.01	20662145	8.66	8.12	V	2-Naphthalenemethanol, 2,3,4,4a,5,6,7,8-octahydro-.alpha.,.alpha.,4a,8-tetramethyl-, [2R-(2.alpha.,4a.beta.,8.beta.)]-
4	64.448	64.205	64.505	TIC	200664529	3.60	18536690	7.77	10.83	V	1H-Cycloprop[e]azulen-4-ol, decahydro-1,1,4,7-tetramethyl-, [1ar-(1a.alpha.,4.alpha.,4a.beta.,7.alpha.,7a.beta.,7b.alpha.)]- (C
5	64.954	64.505	65.330	TIC	546660813	9.81	27762372	11.64	19.69		2,4a,8,8-Tetramethyldecahydrocyclopropa[d]naphthalene
6	65.747	65.355	65.980	TIC	84776487	1.52	5818687	2.44	14.57		4,4,8-Trimethyltricyclo[6.3.1.0(1,5)]dodecane-2,9-diol
7	66.888	66.655	67.155	TIC	31237976	0.56	1926018	0.81	16.22	V	cis-Z-.alpha.-Bisabolene epoxide
8	68.638	67.155	69.055	TIC	2952036108	52.99	52363270	21.94	56.38		Caryophyllene oxide
9	69.254	69.055	69.755	TIC	52389610	0.94	5588803	2.34	9.37	V	trans-p-Mentha-2,8-dienol
10	72.652	72.505	73.055	TIC	37926338	0.68	1665225	0.70	22.78	V	3-Methyl-2-(2-methylene-cyclohexyl)-butan-2-ol
11	77.255	76.855	77.655	TIC	30074353	0.54	898509	0.38	33.47	V	Benzene, 1-[1,1-dimethylethyl]-4-[2-propenyloxy]-
12	84.431	83.705	84.655	TIC	716719656	12.87	29121447	12.21	24.61		3-PROPYL-CANNABIDIOL
13	86.841	86.405	87.355	TIC	238742317	4.29	14713992	6.17	16.23		Baimuxinal
14	93.005	92.805	93.305	TIC	21096299	0.38	2302236	0.97	9.16	V	Cannabichromene

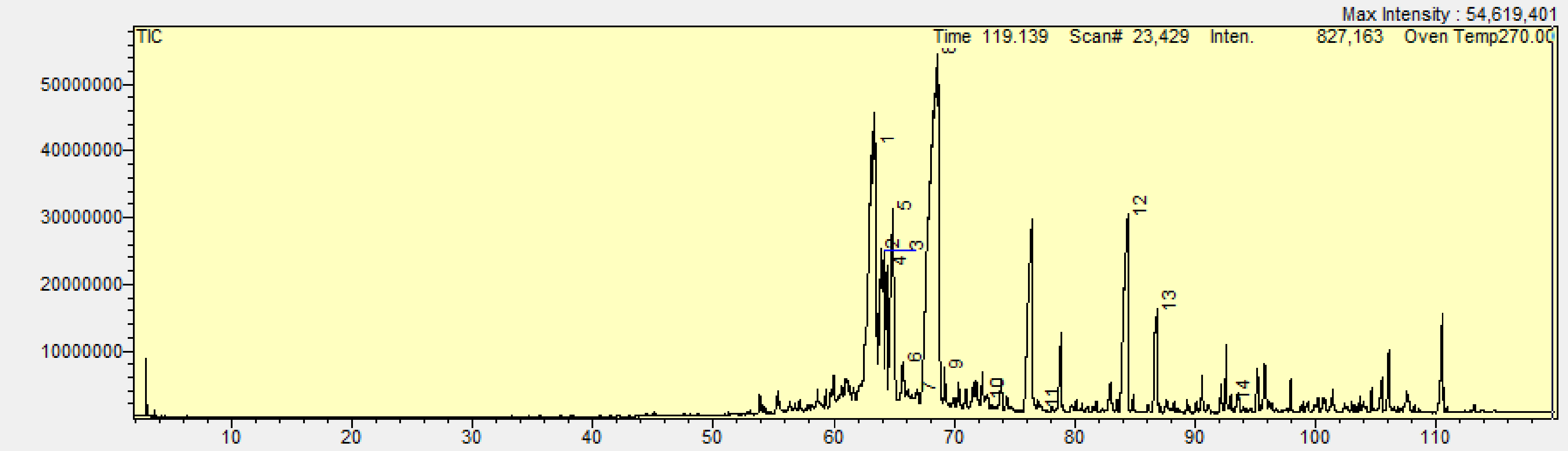

S2. Mechanical statistical analysis

Mechanical outcomes were analyzed within a balanced 4 material system × 5 phytocannabinoid condition factorial design. The tables below provide the complete factorial, simple-effects, Tukey-adjusted, and raw-data outputs used to support Table 4 and the corresponding Results section.

S2.1 Two-way factorial ANOVA

Outcome	Effect	df1	df2	F	Exact p	Partial eta squared
Flexural strength	Material system	3	180	4277.1375	5.172650e-167	0.9862
Flexural strength	Phytocannabinoid condition	4	180	342.1796	6.067518e-83	0.8838
Flexural strength	System × Condition	12	180	2.7783	0.0017	0.1563
Elastic modulus	Material system	3	180	810.1207	3.072542e-104	0.931
Elastic modulus	Phytocannabinoid condition	4	180	1428.5806	3.812408e-135	0.9695
Elastic modulus	System × Condition	12	180	0.005636	1	0.000376
Vickers hardness	Material system	3	180	7369.3512	4.760696e-188	0.9919
Vickers hardness	Phytocannabinoid condition	4	180	1426.5832	4.307218e-135	0.9694
Vickers hardness	System × Condition	12	180	9.4797	3.955892e-14	0.3872
Impact strength	Material system	3	180	14511.6014	2.230747e-214	0.9959
Impact strength	Phytocannabinoid condition	4	180	2036.961	1.191929e-148	0.9784
Impact strength	System × Condition	12	180	11.4165	7.123082e-17	0.4322

S2.2 Simple-effects analyses

Outcome	Simple effect	Level	df1	df2	F	Exact p
Flexural strength	Material system	F1	3	180	919.4388	7.302342e-109
Flexural strength	Material system	F2	3	180	859.8032	2.079546e-106
Flexural strength	Material system	F3	3	180	801.1891	7.773545e-104
Flexural strength	Material system	F4	3	180	732.3279	1.401660e-100
Flexural strength	Material system	Control	3	180	975.4917	4.886302e-111
Flexural strength	Phytocannabinoid condition	PMMA	4	180	54.9683	3.196645e-30
Flexural strength	Phytocannabinoid condition	PMMA/SF	4	180	70.6439	7.179523e-36
Flexural strength	Phytocannabinoid condition	PMMA/HF	4	180	101.8729	3.686627e-45
Flexural strength	Phytocannabinoid condition	PMMA/HF/SF	4	180	123.0294	2.137259e-50
Vickers hardness	Material system	F1	3	180	1678.2062	2.807578e-131
Vickers hardness	Material system	F2	3	180	1508.2172	2.951598e-127
Vickers hardness	Material system	F3	3	180	1346.4932	5.287427e-123
Vickers hardness	Material system	F4	3	180	1165.1755	1.307266e-117
Vickers hardness	Material system	Control	3	180	1709.1781	5.729861e-132
Vickers hardness	Phytocannabinoid condition	PMMA	4	180	238.5066	8.776551e-71
Vickers hardness	Phytocannabinoid condition	PMMA/SF	4	180	305.4727	4.680684e-79
Vickers hardness	Phytocannabinoid condition	PMMA/HF	4	180	424.577	1.783644e-90
Vickers hardness	Phytocannabinoid condition	PMMA/HF/SF	4	180	486.4661	2.614467e-95

Outcome	Simple effect	Level	df1	df2	F	Exact p
Impact strength	Material system	F1	3	180	3173.5713	1.547211e-155
Impact strength	Material system	F2	3	180	2910.5425	3.201769e-152
Impact strength	Material system	F3	3	180	2694.0842	2.899433e-149
Impact strength	Material system	F4	3	180	2446.5923	1.387573e-145
Impact strength	Material system	Control	3	180	3332.4772	2.063649e-157
Impact strength	Phytocannabinoid condition	PMMA	4	180	344.6942	3.391123e-83
Impact strength	Phytocannabinoid condition	PMMA/SF	4	180	446.8471	2.768635e-92
Impact strength	Phytocannabinoid condition	PMMA/HF	4	180	604.2523	3.982097e-103
Impact strength	Phytocannabinoid condition	PMMA/HF/SF	4	180	675.417	3.450802e-107

S2.3 Tukey-adjusted post-hoc comparisons

Outcome	Comparison family	Group 1	Group 2	Mean difference	q	Tukey-adjusted p
Flexural strength	Material system within F1	PMMA	PMMA/SF	-16.29	26.3951	0.001
Flexural strength	Material system within F1	PMMA	PMMA/HF	-33.765	54.7103	0.001
Flexural strength	Material system within F1	PMMA	PMMA/HF/SF	-41.992	68.0407	0.001
Flexural strength	Material system within F1	PMMA/SF	PMMA/HF	-17.475	28.3152	0.001
Flexural strength	Material system within F1	PMMA/SF	PMMA/HF/SF	-25.702	41.6456	0.001
Flexural strength	Material system within F1	PMMA/HF	PMMA/HF/SF	-8.227	13.3304	0.001
Flexural strength	Material system within F2	PMMA	PMMA/SF	-15.856	25.6919	0.001
Flexural strength	Material system within F2	PMMA	PMMA/HF	-32.697	52.9798	0.001
Flexural strength	Material system within F2	PMMA	PMMA/HF/SF	-40.618	65.8144	0.001
Flexural strength	Material system within F2	PMMA/SF	PMMA/HF	-16.841	27.2879	0.001
Flexural strength	Material system within F2	PMMA/SF	PMMA/HF/SF	-24.762	40.1225	0.001
Flexural strength	Material system within F2	PMMA/HF	PMMA/HF/SF	-7.921	12.8346	0.001
Flexural strength	Material system within F3	PMMA	PMMA/SF	-15.338	24.8526	0.001
Flexural strength	Material system within F3	PMMA	PMMA/HF	-31.528	51.0856	0.001
Flexural strength	Material system within F3	PMMA	PMMA/HF/SF	-39.24	63.5816	0.001
Flexural strength	Material system within F3	PMMA/SF	PMMA/HF	-16.19	26.2331	0.001
Flexural strength	Material system within F3	PMMA/SF	PMMA/HF/SF	-23.902	38.729	0.001
Flexural strength	Material system within F3	PMMA/HF	PMMA/HF/SF	-7.712	12.496	0.001
Flexural strength	Material system within F4	PMMA	PMMA/SF	-14.984	24.279	0.001
Flexural strength	Material system within F4	PMMA	PMMA/HF	-30.158	48.8658	0.001
Flexural strength	Material system within F4	PMMA	PMMA/HF/SF	-37.617	60.9518	0.001
Flexural strength	Material system within F4	PMMA/SF	PMMA/HF	-15.174	24.5868	0.001
Flexural strength	Material system within F4	PMMA/SF	PMMA/HF/SF	-22.633	36.6728	0.001
Flexural strength	Material system within F4	PMMA/HF	PMMA/HF/SF	-7.459	12.086	0.001
Flexural strength	Material system within Control	PMMA	PMMA/SF	-16.421	26.6074	0.001
Flexural strength	Material system within Control	PMMA	PMMA/HF	-34.2	55.4151	0.001
Flexural strength	Material system within Control	PMMA	PMMA/HF/SF	-43.445	70.3951	0.001
Flexural strength	Material system within Control	PMMA/SF	PMMA/HF	-17.779	28.8078	0.001
Flexural strength	Material system within Control	PMMA/SF	PMMA/HF/SF	-27.024	43.7877	0.001
Flexural strength	Material system within Control	PMMA/HF	PMMA/HF/SF	-9.245	14.9799	0.001
Flexural strength	Condition within PMMA	F1	F2	2.907	4.7103	0.009185
Flexural strength	Condition within PMMA	F1	F3	6.164	9.9877	0.001
Flexural strength	Condition within PMMA	F1	F4	9.612	15.5746	0.001

Outcome	Comparison family	Group 1	Group 2	Mean difference	q	Tukey-adjusted p
Flexural strength	Condition within PMMA	F1	Control	-1.671	2.7076	0.3134
Flexural strength	Condition within PMMA	F2	F3	3.257	5.2774	0.002345
Flexural strength	Condition within PMMA	F2	F4	6.705	10.8643	0.001
Flexural strength	Condition within PMMA	F2	Control	-4.578	7.4179	0.001
Flexural strength	Condition within PMMA	F3	F4	3.448	5.5869	0.001048
Flexural strength	Condition within PMMA	F3	Control	-7.835	12.6953	0.001
Flexural strength	Condition within PMMA	F4	Control	-11.283	18.2821	0.001
Flexural strength	Condition within PMMA/SF	F1	F2	3.341	5.4135	0.001656
Flexural strength	Condition within PMMA/SF	F1	F3	7.116	11.5302	0.001
Flexural strength	Condition within PMMA/SF	F1	F4	10.918	17.6907	0.001
Flexural strength	Condition within PMMA/SF	F1	Control	-1.802	2.9198	0.24
Flexural strength	Condition within PMMA/SF	F2	F3	3.775	6.1167	0.001
Flexural strength	Condition within PMMA/SF	F2	F4	7.577	12.2772	0.001
Flexural strength	Condition within PMMA/SF	F2	Control	-5.143	8.3333	0.001
Flexural strength	Condition within PMMA/SF	F3	F4	3.802	6.1605	0.001
Flexural strength	Condition within PMMA/SF	F3	Control	-8.918	14.4501	0.001
Flexural strength	Condition within PMMA/SF	F4	Control	-12.72	20.6105	0.001
Flexural strength	Condition within PMMA/HF	F1	F2	3.975	6.4408	0.001
Flexural strength	Condition within PMMA/HF	F1	F3	8.401	13.6124	0.001
Flexural strength	Condition within PMMA/HF	F1	F4	13.219	21.4191	0.001
Flexural strength	Condition within PMMA/HF	F1	Control	-2.106	3.4124	0.1167
Flexural strength	Condition within PMMA/HF	F2	F3	4.426	7.1716	0.001
Flexural strength	Condition within PMMA/HF	F2	F4	9.244	14.9783	0.001
Flexural strength	Condition within PMMA/HF	F2	Control	-6.081	9.8532	0.001
Flexural strength	Condition within PMMA/HF	F3	F4	4.818	7.8067	0.001
Flexural strength	Condition within PMMA/HF	F3	Control	-10.507	17.0248	0.001
Flexural strength	Condition within PMMA/HF	F4	Control	-15.325	24.8315	0.001
Flexural strength	Condition within PMMA/HF/SF	F1	F2	4.281	6.9366	0.001
Flexural strength	Condition within PMMA/HF/SF	F1	F3	8.916	14.4468	0.001
Flexural strength	Condition within PMMA/HF/SF	F1	F4	13.987	22.6635	0.001
Flexural strength	Condition within PMMA/HF/SF	F1	Control	-3.124	5.0619	0.004009
Flexural strength	Condition within PMMA/HF/SF	F2	F3	4.635	7.5102	0.001
Flexural strength	Condition within PMMA/HF/SF	F2	F4	9.706	15.7269	0.001
Flexural strength	Condition within PMMA/HF/SF	F2	Control	-7.405	11.9985	0.001
Flexural strength	Condition within PMMA/HF/SF	F3	F4	5.071	8.2167	0.001
Flexural strength	Condition within PMMA/HF/SF	F3	Control	-12.04	19.5087	0.001
Flexural strength	Condition within PMMA/HF/SF	F4	Control	-17.111	27.7254	0.001
Vickers hardness	Material system within F1	PMMA	PMMA/SF	-2.429	34.4766	0.001
Vickers hardness	Material system within F1	PMMA	PMMA/HF	-4.529	64.2834	0.001
Vickers hardness	Material system within F1	PMMA	PMMA/HF/SF	-6.749	95.7935	0.001
Vickers hardness	Material system within F1	PMMA/SF	PMMA/HF	-2.1	29.8068	0.001
Vickers hardness	Material system within F1	PMMA/SF	PMMA/HF/SF	-4.32	61.3169	0.001
Vickers hardness	Material system within F1	PMMA/HF	PMMA/HF/SF	-2.22	31.5101	0.001
Vickers hardness	Material system within F2	PMMA	PMMA/SF	-2.308	32.7591	0.001
Vickers hardness	Material system within F2	PMMA	PMMA/HF	-4.268	60.5788	0.001
Vickers hardness	Material system within F2	PMMA	PMMA/HF/SF	-6.408	90.9534	0.001
Vickers hardness	Material system within F2	PMMA/SF	PMMA/HF	-1.96	27.8197	0.001

Outcome	Comparison family	Group 1	Group 2	Mean difference	q	Tukey-adjusted p
Vickers hardness	Material system within F2	PMMA/SF	PMMA/HF/SF	-4.1	58.1943	0.001
Vickers hardness	Material system within F2	PMMA/HF	PMMA/HF/SF	-2.14	30.3746	0.001
Vickers hardness	Material system within F3	PMMA	PMMA/SF	-2.236	31.7372	0.001
Vickers hardness	Material system within F3	PMMA	PMMA/HF	-4.016	57.002	0.001
Vickers hardness	Material system within F3	PMMA	PMMA/HF/SF	-6.076	86.2411	0.001
Vickers hardness	Material system within F3	PMMA/SF	PMMA/HF	-1.78	25.2648	0.001
Vickers hardness	Material system within F3	PMMA/SF	PMMA/HF/SF	-3.84	54.5039	0.001
Vickers hardness	Material system within F3	PMMA/HF	PMMA/HF/SF	-2.06	29.2391	0.001
Vickers hardness	Material system within F4	PMMA	PMMA/SF	-2.178	30.9139	0.001
Vickers hardness	Material system within F4	PMMA	PMMA/HF	-3.738	53.0562	0.001
Vickers hardness	Material system within F4	PMMA	PMMA/HF/SF	-5.678	80.592	0.001
Vickers hardness	Material system within F4	PMMA/SF	PMMA/HF	-1.56	22.1422	0.001
Vickers hardness	Material system within F4	PMMA/SF	PMMA/HF/SF	-3.5	49.6781	0.001
Vickers hardness	Material system within F4	PMMA/HF	PMMA/HF/SF	-1.94	27.5358	0.001
Vickers hardness	Material system within Control	PMMA	PMMA/SF	-2.534	35.9669	0.001
Vickers hardness	Material system within Control	PMMA	PMMA/HF	-4.634	65.7737	0.001
Vickers hardness	Material system within Control	PMMA	PMMA/HF/SF	-6.814	96.7161	0.001
Vickers hardness	Material system within Control	PMMA/SF	PMMA/HF	-2.1	29.8068	0.001
Vickers hardness	Material system within Control	PMMA/SF	PMMA/HF/SF	-4.28	60.7492	0.001
Vickers hardness	Material system within Control	PMMA/HF	PMMA/HF/SF	-2.18	30.9423	0.001
Vickers hardness	Condition within PMMA	F1	F2	0.599	8.502	0.001
Vickers hardness	Condition within PMMA	F1	F3	1.307	18.5512	0.001
Vickers hardness	Condition within PMMA	F1	F4	1.969	27.9475	0.001
Vickers hardness	Condition within PMMA	F1	Control	-0.815	11.5679	0.001
Vickers hardness	Condition within PMMA	F2	F3	0.708	10.0492	0.001
Vickers hardness	Condition within PMMA	F2	F4	1.37	19.4454	0.001
Vickers hardness	Condition within PMMA	F2	Control	-1.414	20.0699	0.001
Vickers hardness	Condition within PMMA	F3	F4	0.662	9.3962	0.001
Vickers hardness	Condition within PMMA	F3	Control	-2.122	30.1191	0.001
Vickers hardness	Condition within PMMA	F4	Control	-2.784	39.5153	0.001
Vickers hardness	Condition within PMMA/SF	F1	F2	0.72	10.2195	0.001
Vickers hardness	Condition within PMMA/SF	F1	F3	1.5	21.2906	0.001
Vickers hardness	Condition within PMMA/SF	F1	F4	2.22	31.5101	0.001
Vickers hardness	Condition within PMMA/SF	F1	Control	-0.92	13.0582	0.001
Vickers hardness	Condition within PMMA/SF	F2	F3	0.78	11.0711	0.001
Vickers hardness	Condition within PMMA/SF	F2	F4	1.5	21.2906	0.001
Vickers hardness	Condition within PMMA/SF	F2	Control	-1.64	23.2777	0.001
Vickers hardness	Condition within PMMA/SF	F3	F4	0.72	10.2195	0.001
Vickers hardness	Condition within PMMA/SF	F3	Control	-2.42	34.3488	0.001
Vickers hardness	Condition within PMMA/SF	F4	Control	-3.14	44.5683	0.001
Vickers hardness	Condition within PMMA/HF	F1	F2	0.86	12.2066	0.001
Vickers hardness	Condition within PMMA/HF	F1	F3	1.82	25.8326	0.001
Vickers hardness	Condition within PMMA/HF	F1	F4	2.76	39.1747	0.001
Vickers hardness	Condition within PMMA/HF	F1	Control	-0.92	13.0582	0.001
Vickers hardness	Condition within PMMA/HF	F2	F3	0.96	13.626	0.001
Vickers hardness	Condition within PMMA/HF	F2	F4	1.9	26.9681	0.001
Vickers hardness	Condition within PMMA/HF	F2	Control	-1.78	25.2648	0.001

Outcome	Comparison family	Group 1	Group 2	Mean difference	q	Tukey-adjusted p
Vickers hardness	Condition within PMMA/HF	F3	F4	0.94	13.3421	0.001
Vickers hardness	Condition within PMMA/HF	F3	Control	-2.74	38.8908	0.001
Vickers hardness	Condition within PMMA/HF	F4	Control	-3.68	52.2329	0.001
Vickers hardness	Condition within PMMA/HF/SF	F1	F2	0.94	13.3421	0.001
Vickers hardness	Condition within PMMA/HF/SF	F1	F3	1.98	28.1036	0.001
Vickers hardness	Condition within PMMA/HF/SF	F1	F4	3.04	43.1489	0.001
Vickers hardness	Condition within PMMA/HF/SF	F1	Control	-0.88	12.4905	0.001
Vickers hardness	Condition within PMMA/HF/SF	F2	F3	1.04	14.7615	0.001
Vickers hardness	Condition within PMMA/HF/SF	F2	F4	2.1	29.8068	0.001
Vickers hardness	Condition within PMMA/HF/SF	F2	Control	-1.82	25.8326	0.001
Vickers hardness	Condition within PMMA/HF/SF	F3	F4	1.06	15.0454	0.001
Vickers hardness	Condition within PMMA/HF/SF	F3	Control	-2.86	40.5941	0.001
Vickers hardness	Condition within PMMA/HF/SF	F4	Control	-3.92	55.6394	0.001
Impact strength	Material system within F1	PMMA	PMMA/SF	-2.916	42.5397	0.001
Impact strength	Material system within F1	PMMA	PMMA/HF	-6.006	87.6177	0.001
Impact strength	Material system within F1	PMMA	PMMA/HF/SF	-8.94	130.42	0.001
Impact strength	Material system within F1	PMMA/SF	PMMA/HF	-3.09	45.078	0.001
Impact strength	Material system within F1	PMMA/SF	PMMA/HF/SF	-6.024	87.8803	0.001
Impact strength	Material system within F1	PMMA/HF	PMMA/HF/SF	-2.934	42.8023	0.001
Impact strength	Material system within F2	PMMA	PMMA/SF	-2.78	40.5556	0.001
Impact strength	Material system within F2	PMMA	PMMA/HF	-5.684	82.9202	0.001
Impact strength	Material system within F2	PMMA	PMMA/HF/SF	-8.58	125.1681	0.001
Impact strength	Material system within F2	PMMA/SF	PMMA/HF	-2.904	42.3646	0.001
Impact strength	Material system within F2	PMMA/SF	PMMA/HF/SF	-5.8	84.6125	0.001
Impact strength	Material system within F2	PMMA/HF	PMMA/HF/SF	-2.896	42.2479	0.001
Impact strength	Material system within F3	PMMA	PMMA/SF	-2.68	39.0968	0.001
Impact strength	Material system within F3	PMMA	PMMA/HF	-5.402	78.8063	0.001
Impact strength	Material system within F3	PMMA	PMMA/HF/SF	-8.278	120.7625	0.001
Impact strength	Material system within F3	PMMA/SF	PMMA/HF	-2.722	39.7095	0.001
Impact strength	Material system within F3	PMMA/SF	PMMA/HF/SF	-5.598	81.6656	0.001
Impact strength	Material system within F3	PMMA/HF	PMMA/HF/SF	-2.876	41.9561	0.001
Impact strength	Material system within F4	PMMA	PMMA/SF	-2.532	36.9377	0.001
Impact strength	Material system within F4	PMMA	PMMA/HF	-5.1	74.4006	0.001
Impact strength	Material system within F4	PMMA	PMMA/HF/SF	-7.896	115.1897	0.001
Impact strength	Material system within F4	PMMA/SF	PMMA/HF	-2.568	37.4629	0.001
Impact strength	Material system within F4	PMMA/SF	PMMA/HF/SF	-5.364	78.252	0.001
Impact strength	Material system within F4	PMMA/HF	PMMA/HF/SF	-2.796	40.7891	0.001
Impact strength	Material system within Control	PMMA	PMMA/SF	-2.972	43.3566	0.001
Impact strength	Material system within Control	PMMA	PMMA/HF	-6.1	88.989	0.001
Impact strength	Material system within Control	PMMA	PMMA/HF/SF	-9.174	133.8336	0.001
Impact strength	Material system within Control	PMMA/SF	PMMA/HF	-3.128	45.6324	0.001
Impact strength	Material system within Control	PMMA/SF	PMMA/HF/SF	-6.202	90.477	0.001
Impact strength	Material system within Control	PMMA/HF	PMMA/HF/SF	-3.074	44.8446	0.001
Impact strength	Condition within PMMA	F1	F2	0.762	11.1163	0.001
Impact strength	Condition within PMMA	F1	F3	1.6	23.3414	0.001
Impact strength	Condition within PMMA	F1	F4	2.378	34.6911	0.001
Impact strength	Condition within PMMA	F1	Control	-0.846	12.3418	0.001

Outcome	Comparison family	Group 1	Group 2	Mean difference	q	Tukey-adjusted p
Impact strength	Condition within PMMA	F2	F3	0.838	12.225	0.001
Impact strength	Condition within PMMA	F2	F4	1.616	23.5748	0.001
Impact strength	Condition within PMMA	F2	Control	-1.608	23.4581	0.001
Impact strength	Condition within PMMA	F3	F4	0.778	11.3497	0.001
Impact strength	Condition within PMMA	F3	Control	-2.446	35.6831	0.001
Impact strength	Condition within PMMA	F4	Control	-3.224	47.0329	0.001
Impact strength	Condition within PMMA/SF	F1	F2	0.898	13.1003	0.001
Impact strength	Condition within PMMA/SF	F1	F3	1.836	26.7842	0.001
Impact strength	Condition within PMMA/SF	F1	F4	2.762	40.2931	0.001
Impact strength	Condition within PMMA/SF	F1	Control	-0.902	13.1587	0.001
Impact strength	Condition within PMMA/SF	F2	F3	0.938	13.6839	0.001
Impact strength	Condition within PMMA/SF	F2	F4	1.864	27.1927	0.001
Impact strength	Condition within PMMA/SF	F2	Control	-1.8	26.2591	0.001
Impact strength	Condition within PMMA/SF	F3	F4	0.926	13.5088	0.001
Impact strength	Condition within PMMA/SF	F3	Control	-2.738	39.9429	0.001
Impact strength	Condition within PMMA/SF	F4	Control	-3.664	53.4518	0.001
Impact strength	Condition within PMMA/HF	F1	F2	1.084	15.8138	0.001
Impact strength	Condition within PMMA/HF	F1	F3	2.204	32.1527	0.001
Impact strength	Condition within PMMA/HF	F1	F4	3.284	47.9082	0.001
Impact strength	Condition within PMMA/HF	F1	Control	-0.94	13.7131	0.001
Impact strength	Condition within PMMA/HF	F2	F3	1.12	16.339	0.001
Impact strength	Condition within PMMA/HF	F2	F4	2.2	32.0944	0.001
Impact strength	Condition within PMMA/HF	F2	Control	-2.024	29.5268	0.001
Impact strength	Condition within PMMA/HF	F3	F4	1.08	15.7554	0.001
Impact strength	Condition within PMMA/HF	F3	Control	-3.144	45.8658	0.001
Impact strength	Condition within PMMA/HF	F4	Control	-4.224	61.6212	0.001
Impact strength	Condition within PMMA/HF/SF	F1	F2	1.122	16.3681	0.001
Impact strength	Condition within PMMA/HF/SF	F1	F3	2.262	32.9989	0.001
Impact strength	Condition within PMMA/HF/SF	F1	F4	3.422	49.9214	0.001
Impact strength	Condition within PMMA/HF/SF	F1	Control	-1.08	15.7554	0.001
Impact strength	Condition within PMMA/HF/SF	F2	F3	1.14	16.6307	0.001
Impact strength	Condition within PMMA/HF/SF	F2	F4	2.3	33.5532	0.001
Impact strength	Condition within PMMA/HF/SF	F2	Control	-2.202	32.1236	0.001
Impact strength	Condition within PMMA/HF/SF	F3	F4	1.16	16.9225	0.001
Impact strength	Condition within PMMA/HF/SF	F3	Control	-3.342	48.7543	0.001
Impact strength	Condition within PMMA/HF/SF	F4	Control	-4.502	65.6768	0.001

S2.4 Raw mechanical data used for reanalysis

System	Condition	Value	Outcome
PMMA	F1	92.84	Flexural strength
PMMA	F1	2.31	Elastic modulus
PMMA	F2	89.76	Flexural strength
PMMA	F2	2.24	Elastic modulus
PMMA	F3	86.42	Flexural strength
PMMA	F3	2.16	Elastic modulus
PMMA	F4	82.95	Flexural strength

System	Condition	Value	Outcome
PMMA	F4	2.08	Elastic modulus
PMMA	Control	94.6	Flexural strength
PMMA	Control	2.36	Elastic modulus
PMMA	F1	93.15	Flexural strength
PMMA	F1	2.32	Elastic modulus
PMMA	F2	90.24	Flexural strength
PMMA	F2	2.25	Elastic modulus
PMMA	F3	87.05	Flexural strength
PMMA	F3	2.17	Elastic modulus
PMMA	F4	83.41	Flexural strength
PMMA	F4	2.09	Elastic modulus
PMMA	Control	95.12	Flexural strength
PMMA	Control	2.35	Elastic modulus
PMMA	F1	91.72	Flexural strength
PMMA	F1	2.29	Elastic modulus
PMMA	F2	88.95	Flexural strength
PMMA	F2	2.22	Elastic modulus
PMMA	F3	85.84	Flexural strength
PMMA	F3	2.14	Elastic modulus
PMMA	F4	81.96	Flexural strength
PMMA	F4	2.05	Elastic modulus
PMMA	Control	90.52	Flexural strength
PMMA	Control	2.34	Elastic modulus
PMMA	F1	93.48	Flexural strength
PMMA	F1	2.33	Elastic modulus
PMMA	F2	90.62	Flexural strength
PMMA	F2	2.26	Elastic modulus
PMMA	F3	87.31	Flexural strength
PMMA	F3	2.18	Elastic modulus
PMMA	F4	84.02	Flexural strength
PMMA	F4	2.1	Elastic modulus
PMMA	Control	95.44	Flexural strength
PMMA	Control	2.35	Elastic modulus
PMMA	F1	92.26	Flexural strength
PMMA	F1	2.3	Elastic modulus
PMMA	F2	89.41	Flexural strength
PMMA	F2	2.23	Elastic modulus
PMMA	F3	86.13	Flexural strength
PMMA	F3	2.15	Elastic modulus
PMMA	F4	82.74	Flexural strength
PMMA	F4	2.07	Elastic modulus
PMMA	Control	92.42	Flexural strength
PMMA	Control	2.35	Elastic modulus
PMMA	F1	92.63	Flexural strength
PMMA	F1	2.31	Elastic modulus
PMMA	F2	89.87	Flexural strength
PMMA	F2	2.24	Elastic modulus

System	Condition	Value	Outcome
PMMA	F3	86.55	Flexural strength
PMMA	F3	2.16	Elastic modulus
PMMA	F4	83.12	Flexural strength
PMMA	F4	2.08	Elastic modulus
PMMA	Control	92.82	Flexural strength
PMMA	Control	2.38	Elastic modulus
PMMA	F1	94.11	Flexural strength
PMMA	F1	2.35	Elastic modulus
PMMA	F2	91.35	Flexural strength
PMMA	F2	2.28	Elastic modulus
PMMA	F3	88.06	Flexural strength
PMMA	F3	2.2	Elastic modulus
PMMA	F4	84.86	Flexural strength
PMMA	F4	2.12	Elastic modulus
PMMA	Control	98.63	Flexural strength
PMMA	Control	2.38	Elastic modulus
PMMA	F1	92.95	Flexural strength
PMMA	F1	2.32	Elastic modulus
PMMA	F2	89.94	Flexural strength
PMMA	F2	2.25	Elastic modulus
PMMA	F3	86.74	Flexural strength
PMMA	F3	2.17	Elastic modulus
PMMA	F4	83.27	Flexural strength
PMMA	F4	2.09	Elastic modulus
PMMA	Control	94.68	Flexural strength
PMMA	Control	2.38	Elastic modulus
PMMA	F1	92.34	Flexural strength
PMMA	F1	2.3	Elastic modulus
PMMA	F2	89.22	Flexural strength
PMMA	F2	2.23	Elastic modulus
PMMA	F3	85.96	Flexural strength
PMMA	F3	2.15	Elastic modulus
PMMA	F4	82.58	Flexural strength
PMMA	F4	2.07	Elastic modulus
PMMA	Control	93.7	Flexural strength
PMMA	Control	2.37	Elastic modulus
PMMA	F1	93.76	Flexural strength
PMMA	F1	2.34	Elastic modulus
PMMA	F2	90.81	Flexural strength
PMMA	F2	2.27	Elastic modulus
PMMA	F3	87.54	Flexural strength
PMMA	F3	2.19	Elastic modulus
PMMA	F4	84.21	Flexural strength
PMMA	F4	2.11	Elastic modulus
PMMA	Control	98.02	Flexural strength
PMMA	Control	2.4	Elastic modulus
PMMA/SF	F1	109.24	Flexural strength

System	Condition	Value	Outcome
PMMA/SF	F1	2.38	Elastic modulus
PMMA/SF	F2	105.82	Flexural strength
PMMA/SF	F2	2.31	Elastic modulus
PMMA/SF	F3	101.94	Flexural strength
PMMA/SF	F3	2.23	Elastic modulus
PMMA/SF	F4	98.15	Flexural strength
PMMA/SF	F4	2.15	Elastic modulus
PMMA/SF	Control	111.03	Flexural strength
PMMA/SF	Control	2.43	Elastic modulus
PMMA/SF	F1	109.86	Flexural strength
PMMA/SF	F1	2.37	Elastic modulus
PMMA/SF	F2	106.41	Flexural strength
PMMA/SF	F2	2.3	Elastic modulus
PMMA/SF	F3	102.55	Flexural strength
PMMA/SF	F3	2.22	Elastic modulus
PMMA/SF	F4	98.74	Flexural strength
PMMA/SF	F4	2.14	Elastic modulus
PMMA/SF	Control	111.65	Flexural strength
PMMA/SF	Control	2.42	Elastic modulus
PMMA/SF	F1	104.52	Flexural strength
PMMA/SF	F1	2.36	Elastic modulus
PMMA/SF	F2	101.33	Flexural strength
PMMA/SF	F2	2.29	Elastic modulus
PMMA/SF	F3	97.84	Flexural strength
PMMA/SF	F3	2.21	Elastic modulus
PMMA/SF	F4	94.25	Flexural strength
PMMA/SF	F4	2.13	Elastic modulus
PMMA/SF	Control	106.25	Flexural strength
PMMA/SF	Control	2.41	Elastic modulus
PMMA/SF	F1	110.24	Flexural strength
PMMA/SF	F1	2.37	Elastic modulus
PMMA/SF	F2	106.95	Flexural strength
PMMA/SF	F2	2.3	Elastic modulus
PMMA/SF	F3	103.12	Flexural strength
PMMA/SF	F3	2.22	Elastic modulus
PMMA/SF	F4	99.36	Flexural strength
PMMA/SF	F4	2.14	Elastic modulus
PMMA/SF	Control	112.03	Flexural strength
PMMA/SF	Control	2.42	Elastic modulus
PMMA/SF	F1	106.71	Flexural strength
PMMA/SF	F1	2.37	Elastic modulus
PMMA/SF	F2	103.46	Flexural strength
PMMA/SF	F2	2.3	Elastic modulus
PMMA/SF	F3	99.74	Flexural strength
PMMA/SF	F3	2.22	Elastic modulus
PMMA/SF	F4	96.05	Flexural strength
PMMA/SF	F4	2.14	Elastic modulus

System	Condition	Value	Outcome
PMMA/SF	Control	108.45	Flexural strength
PMMA/SF	Control	2.42	Elastic modulus
PMMA/SF	F1	107.15	Flexural strength
PMMA/SF	F1	2.4	Elastic modulus
PMMA/SF	F2	103.88	Flexural strength
PMMA/SF	F2	2.33	Elastic modulus
PMMA/SF	F3	100.22	Flexural strength
PMMA/SF	F3	2.25	Elastic modulus
PMMA/SF	F4	96.54	Flexural strength
PMMA/SF	F4	2.17	Elastic modulus
PMMA/SF	Control	108.92	Flexural strength
PMMA/SF	Control	2.45	Elastic modulus
PMMA/SF	F1	113.72	Flexural strength
PMMA/SF	F1	2.4	Elastic modulus
PMMA/SF	F2	110.18	Flexural strength
PMMA/SF	F2	2.33	Elastic modulus
PMMA/SF	F3	106.34	Flexural strength
PMMA/SF	F3	2.25	Elastic modulus
PMMA/SF	F4	102.25	Flexural strength
PMMA/SF	F4	2.17	Elastic modulus
PMMA/SF	Control	115.73	Flexural strength
PMMA/SF	Control	2.45	Elastic modulus
PMMA/SF	F1	109.41	Flexural strength
PMMA/SF	F1	2.4	Elastic modulus
PMMA/SF	F2	106.08	Flexural strength
PMMA/SF	F2	2.33	Elastic modulus
PMMA/SF	F3	102.26	Flexural strength
PMMA/SF	F3	2.25	Elastic modulus
PMMA/SF	F4	98.46	Flexural strength
PMMA/SF	F4	2.17	Elastic modulus
PMMA/SF	Control	111.12	Flexural strength
PMMA/SF	Control	2.45	Elastic modulus
PMMA/SF	F1	108.24	Flexural strength
PMMA/SF	F1	2.39	Elastic modulus
PMMA/SF	F2	104.96	Flexural strength
PMMA/SF	F2	2.32	Elastic modulus
PMMA/SF	F3	101.15	Flexural strength
PMMA/SF	F3	2.24	Elastic modulus
PMMA/SF	F4	97.42	Flexural strength
PMMA/SF	F4	2.16	Elastic modulus
PMMA/SF	Control	109.97	Flexural strength
PMMA/SF	Control	2.44	Elastic modulus
PMMA/SF	F1	113.05	Flexural strength
PMMA/SF	F1	2.42	Elastic modulus
PMMA/SF	F2	109.66	Flexural strength
PMMA/SF	F2	2.35	Elastic modulus
PMMA/SF	F3	105.82	Flexural strength

System	Condition	Value	Outcome
PMMA/SF	F3	2.27	Elastic modulus
PMMA/SF	F4	101.74	Flexural strength
PMMA/SF	F4	2.19	Elastic modulus
PMMA/SF	Control	115.01	Flexural strength
PMMA/SF	Control	2.47	Elastic modulus
PMMA/HF	F1	126.58	Flexural strength
PMMA/HF	F1	2.3	Elastic modulus
PMMA/HF	F2	122.38	Flexural strength
PMMA/HF	F2	2.23	Elastic modulus
PMMA/HF	F3	117.83	Flexural strength
PMMA/HF	F3	2.15	Elastic modulus
PMMA/HF	F4	112.99	Flexural strength
PMMA/HF	F4	2.07	Elastic modulus
PMMA/HF	Control	128.79	Flexural strength
PMMA/HF	Control	2.35	Elastic modulus
PMMA/HF	F1	127	Flexural strength
PMMA/HF	F1	2.31	Elastic modulus
PMMA/HF	F2	123.03	Flexural strength
PMMA/HF	F2	2.24	Elastic modulus
PMMA/HF	F3	118.69	Flexural strength
PMMA/HF	F3	2.16	Elastic modulus
PMMA/HF	F4	113.61	Flexural strength
PMMA/HF	F4	2.08	Elastic modulus
PMMA/HF	Control	129.5	Flexural strength
PMMA/HF	Control	2.34	Elastic modulus
PMMA/HF	F1	125.05	Flexural strength
PMMA/HF	F1	2.28	Elastic modulus
PMMA/HF	F2	121.27	Flexural strength
PMMA/HF	F2	2.21	Elastic modulus
PMMA/HF	F3	117.06	Flexural strength
PMMA/HF	F3	2.13	Elastic modulus
PMMA/HF	F4	111.63	Flexural strength
PMMA/HF	F4	2.04	Elastic modulus
PMMA/HF	Control	123.24	Flexural strength
PMMA/HF	Control	2.33	Elastic modulus
PMMA/HF	F1	127.45	Flexural strength
PMMA/HF	F1	2.32	Elastic modulus
PMMA/HF	F2	123.55	Flexural strength
PMMA/HF	F2	2.25	Elastic modulus
PMMA/HF	F3	119.04	Flexural strength
PMMA/HF	F3	2.17	Elastic modulus
PMMA/HF	F4	114.43	Flexural strength
PMMA/HF	F4	2.09	Elastic modulus
PMMA/HF	Control	129.94	Flexural strength
PMMA/HF	Control	2.34	Elastic modulus
PMMA/HF	F1	125.79	Flexural strength
PMMA/HF	F1	2.29	Elastic modulus

System	Condition	Value	Outcome
PMMA/HF	F2	121.9	Flexural strength
PMMA/HF	F2	2.22	Elastic modulus
PMMA/HF	F3	117.43	Flexural strength
PMMA/HF	F3	2.14	Elastic modulus
PMMA/HF	F4	112.7	Flexural strength
PMMA/HF	F4	2.06	Elastic modulus
PMMA/HF	Control	125.85	Flexural strength
PMMA/HF	Control	2.34	Elastic modulus
PMMA/HF	F1	126.29	Flexural strength
PMMA/HF	F1	2.3	Elastic modulus
PMMA/HF	F2	122.53	Flexural strength
PMMA/HF	F2	2.23	Elastic modulus
PMMA/HF	F3	118	Flexural strength
PMMA/HF	F3	2.15	Elastic modulus
PMMA/HF	F4	113.21	Flexural strength
PMMA/HF	F4	2.07	Elastic modulus
PMMA/HF	Control	126.39	Flexural strength
PMMA/HF	Control	2.37	Elastic modulus
PMMA/HF	F1	128.3	Flexural strength
PMMA/HF	F1	2.34	Elastic modulus
PMMA/HF	F2	124.4	Flexural strength
PMMA/HF	F2	2.27	Elastic modulus
PMMA/HF	F3	119.99	Flexural strength
PMMA/HF	F3	2.19	Elastic modulus
PMMA/HF	F4	115.55	Flexural strength
PMMA/HF	F4	2.11	Elastic modulus
PMMA/HF	Control	134.3	Flexural strength
PMMA/HF	Control	2.37	Elastic modulus
PMMA/HF	F1	126.72	Flexural strength
PMMA/HF	F1	2.31	Elastic modulus
PMMA/HF	F2	122.63	Flexural strength
PMMA/HF	F2	2.24	Elastic modulus
PMMA/HF	F3	118.26	Flexural strength
PMMA/HF	F3	2.16	Elastic modulus
PMMA/HF	F4	113.41	Flexural strength
PMMA/HF	F4	2.08	Elastic modulus
PMMA/HF	Control	128.9	Flexural strength
PMMA/HF	Control	2.37	Elastic modulus
PMMA/HF	F1	125.89	Flexural strength
PMMA/HF	F1	2.29	Elastic modulus
PMMA/HF	F2	121.64	Flexural strength
PMMA/HF	F2	2.22	Elastic modulus
PMMA/HF	F3	117.22	Flexural strength
PMMA/HF	F3	2.14	Elastic modulus
PMMA/HF	F4	112.48	Flexural strength
PMMA/HF	F4	2.06	Elastic modulus
PMMA/HF	Control	127.57	Flexural strength

System	Condition	Value	Outcome
PMMA/HF	Control	2.36	Elastic modulus
PMMA/HF	F1	127.82	Flexural strength
PMMA/HF	F1	2.33	Elastic modulus
PMMA/HF	F2	123.81	Flexural strength
PMMA/HF	F2	2.26	Elastic modulus
PMMA/HF	F3	119.36	Flexural strength
PMMA/HF	F3	2.18	Elastic modulus
PMMA/HF	F4	114.69	Flexural strength
PMMA/HF	F4	2.1	Elastic modulus
PMMA/HF	Control	133.47	Flexural strength
PMMA/HF	Control	2.39	Elastic modulus
PMMA/HF/SF	F1	134.62	Flexural strength
PMMA/HF/SF	F1	2.46	Elastic modulus
PMMA/HF/SF	F2	130.24	Flexural strength
PMMA/HF/SF	F2	2.39	Elastic modulus
PMMA/HF/SF	F3	125.61	Flexural strength
PMMA/HF/SF	F3	2.31	Elastic modulus
PMMA/HF/SF	F4	120.55	Flexural strength
PMMA/HF/SF	F4	2.23	Elastic modulus
PMMA/HF/SF	Control	137.84	Flexural strength
PMMA/HF/SF	Control	2.52	Elastic modulus
PMMA/HF/SF	F1	135.18	Flexural strength
PMMA/HF/SF	F1	2.47	Elastic modulus
PMMA/HF/SF	F2	130.92	Flexural strength
PMMA/HF/SF	F2	2.4	Elastic modulus
PMMA/HF/SF	F3	126.34	Flexural strength
PMMA/HF/SF	F3	2.32	Elastic modulus
PMMA/HF/SF	F4	121.08	Flexural strength
PMMA/HF/SF	F4	2.24	Elastic modulus
PMMA/HF/SF	Control	138.42	Flexural strength
PMMA/HF/SF	Control	2.51	Elastic modulus
PMMA/HF/SF	F1	132.84	Flexural strength
PMMA/HF/SF	F1	2.44	Elastic modulus
PMMA/HF/SF	F2	128.63	Flexural strength
PMMA/HF/SF	F2	2.37	Elastic modulus
PMMA/HF/SF	F3	124.11	Flexural strength
PMMA/HF/SF	F3	2.29	Elastic modulus
PMMA/HF/SF	F4	119.02	Flexural strength
PMMA/HF/SF	F4	2.21	Elastic modulus
PMMA/HF/SF	Control	135.21	Flexural strength
PMMA/HF/SF	Control	2.49	Elastic modulus
PMMA/HF/SF	F1	135.94	Flexural strength
PMMA/HF/SF	F1	2.47	Elastic modulus
PMMA/HF/SF	F2	131.55	Flexural strength
PMMA/HF/SF	F2	2.4	Elastic modulus
PMMA/HF/SF	F3	126.88	Flexural strength
PMMA/HF/SF	F3	2.32	Elastic modulus

System	Condition	Value	Outcome
PMMA/HF/SF	F4	121.92	Flexural strength
PMMA/HF/SF	F4	2.24	Elastic modulus
PMMA/HF/SF	Control	139.05	Flexural strength
PMMA/HF/SF	Control	2.51	Elastic modulus
PMMA/HF/SF	F1	133.42	Flexural strength
PMMA/HF/SF	F1	2.45	Elastic modulus
PMMA/HF/SF	F2	129.18	Flexural strength
PMMA/HF/SF	F2	2.38	Elastic modulus
PMMA/HF/SF	F3	124.63	Flexural strength
PMMA/HF/SF	F3	2.3	Elastic modulus
PMMA/HF/SF	F4	119.64	Flexural strength
PMMA/HF/SF	F4	2.22	Elastic modulus
PMMA/HF/SF	Control	136.47	Flexural strength
PMMA/HF/SF	Control	2.5	Elastic modulus
PMMA/HF/SF	F1	133.95	Flexural strength
PMMA/HF/SF	F1	2.48	Elastic modulus
PMMA/HF/SF	F2	129.74	Flexural strength
PMMA/HF/SF	F2	2.41	Elastic modulus
PMMA/HF/SF	F3	125.18	Flexural strength
PMMA/HF/SF	F3	2.33	Elastic modulus
PMMA/HF/SF	F4	120.11	Flexural strength
PMMA/HF/SF	F4	2.25	Elastic modulus
PMMA/HF/SF	Control	137.02	Flexural strength
PMMA/HF/SF	Control	2.53	Elastic modulus
PMMA/HF/SF	F1	138.12	Flexural strength
PMMA/HF/SF	F1	2.49	Elastic modulus
PMMA/HF/SF	F2	133.84	Flexural strength
PMMA/HF/SF	F2	2.42	Elastic modulus
PMMA/HF/SF	F3	129.03	Flexural strength
PMMA/HF/SF	F3	2.34	Elastic modulus
PMMA/HF/SF	F4	123.88	Flexural strength
PMMA/HF/SF	F4	2.26	Elastic modulus
PMMA/HF/SF	Control	141.66	Flexural strength
PMMA/HF/SF	Control	2.53	Elastic modulus
PMMA/HF/SF	F1	134.35	Flexural strength
PMMA/HF/SF	F1	2.48	Elastic modulus
PMMA/HF/SF	F2	130.05	Flexural strength
PMMA/HF/SF	F2	2.41	Elastic modulus
PMMA/HF/SF	F3	125.42	Flexural strength
PMMA/HF/SF	F3	2.33	Elastic modulus
PMMA/HF/SF	F4	120.36	Flexural strength
PMMA/HF/SF	F4	2.25	Elastic modulus
PMMA/HF/SF	Control	137.63	Flexural strength
PMMA/HF/SF	Control	2.53	Elastic modulus
PMMA/HF/SF	F1	133.18	Flexural strength
PMMA/HF/SF	F1	2.47	Elastic modulus
PMMA/HF/SF	F2	128.94	Flexural strength

System	Condition	Value	Outcome
PMMA/HF/SF	F2	2.4	Elastic modulus
PMMA/HF/SF	F3	124.36	Flexural strength
PMMA/HF/SF	F3	2.32	Elastic modulus
PMMA/HF/SF	F4	119.32	Flexural strength
PMMA/HF/SF	F4	2.24	Elastic modulus
PMMA/HF/SF	Control	136.18	Flexural strength
PMMA/HF/SF	Control	2.52	Elastic modulus
PMMA/HF/SF	F1	137.56	Flexural strength
PMMA/HF/SF	F1	2.5	Elastic modulus
PMMA/HF/SF	F2	133.26	Flexural strength
PMMA/HF/SF	F2	2.43	Elastic modulus
PMMA/HF/SF	F3	128.44	Flexural strength
PMMA/HF/SF	F3	2.35	Elastic modulus
PMMA/HF/SF	F4	123.41	Flexural strength
PMMA/HF/SF	F4	2.27	Elastic modulus
PMMA/HF/SF	Control	140.92	Flexural strength
PMMA/HF/SF	Control	2.55	Elastic modulus
PMMA	F1	18.42	Vickers hardness
PMMA	F2	17.86	Vickers hardness
PMMA	F3	17.14	Vickers hardness
PMMA	F4	16.48	Vickers hardness
PMMA	Control	19.26	Vickers hardness
PMMA	F1	18.55	Vickers hardness
PMMA	F2	17.94	Vickers hardness
PMMA	F3	17.22	Vickers hardness
PMMA	F4	16.56	Vickers hardness
PMMA	Control	19.38	Vickers hardness
PMMA	F1	18.18	Vickers hardness
PMMA	F2	17.52	Vickers hardness
PMMA	F3	16.88	Vickers hardness
PMMA	F4	16.2	Vickers hardness
PMMA	Control	19.02	Vickers hardness
PMMA	F1	18.66	Vickers hardness
PMMA	F2	18.02	Vickers hardness
PMMA	F3	17.34	Vickers hardness
PMMA	F4	16.68	Vickers hardness
PMMA	Control	19.44	Vickers hardness
PMMA	F1	18.34	Vickers hardness
PMMA	F2	17.74	Vickers hardness
PMMA	F3	17.02	Vickers hardness
PMMA	F4	16.36	Vickers hardness
PMMA	Control	19.14	Vickers hardness
PMMA	F1	18.46	Vickers hardness
PMMA	F2	17.88	Vickers hardness
PMMA	F3	17.16	Vickers hardness
PMMA	F4	16.5	Vickers hardness
PMMA	Control	19.28	Vickers hardness

System	Condition	Value	Outcome
PMMA	F1	18.92	Vickers hardness
PMMA	F2	18.36	Vickers hardness
PMMA	F3	17.64	Vickers hardness
PMMA	F4	16.98	Vickers hardness
PMMA	Control	19.72	Vickers hardness
PMMA	F1	18.52	Vickers hardness
PMMA	F2	17.96	Vickers hardness
PMMA	F3	17.24	Vickers hardness
PMMA	F4	16.58	Vickers hardness
PMMA	Control	19.34	Vickers hardness
PMMA	F1	18.3	Vickers hardness
PMMA	F2	17.68	Vickers hardness
PMMA	F3	16.96	Vickers hardness
PMMA	F4	16.3	Vickers hardness
PMMA	Control	19.1	Vickers hardness
PMMA	F1	18.74	Vickers hardness
PMMA	F2	18.14	Vickers hardness
PMMA	F3	17.42	Vickers hardness
PMMA	F4	16.76	Vickers hardness
PMMA	Control	19.56	Vickers hardness
PMMA/SF	F1	20.84	Vickers hardness
PMMA/SF	F2	20.12	Vickers hardness
PMMA/SF	F3	19.34	Vickers hardness
PMMA/SF	F4	18.62	Vickers hardness
PMMA/SF	Control	21.76	Vickers hardness
PMMA/SF	F1	20.96	Vickers hardness
PMMA/SF	F2	20.24	Vickers hardness
PMMA/SF	F3	19.46	Vickers hardness
PMMA/SF	F4	18.74	Vickers hardness
PMMA/SF	Control	21.88	Vickers hardness
PMMA/SF	F1	20.58	Vickers hardness
PMMA/SF	F2	19.86	Vickers hardness
PMMA/SF	F3	19.08	Vickers hardness
PMMA/SF	F4	18.36	Vickers hardness
PMMA/SF	Control	21.5	Vickers hardness
PMMA/SF	F1	21.08	Vickers hardness
PMMA/SF	F2	20.36	Vickers hardness
PMMA/SF	F3	19.58	Vickers hardness
PMMA/SF	F4	18.86	Vickers hardness
PMMA/SF	Control	22	Vickers hardness
PMMA/SF	F1	20.76	Vickers hardness
PMMA/SF	F2	20.04	Vickers hardness
PMMA/SF	F3	19.26	Vickers hardness
PMMA/SF	F4	18.54	Vickers hardness
PMMA/SF	Control	21.68	Vickers hardness
PMMA/SF	F1	20.88	Vickers hardness
PMMA/SF	F2	20.16	Vickers hardness

System	Condition	Value	Outcome
PMMA/SF	F3	19.38	Vickers hardness
PMMA/SF	F4	18.66	Vickers hardness
PMMA/SF	Control	21.8	Vickers hardness
PMMA/SF	F1	21.34	Vickers hardness
PMMA/SF	F2	20.62	Vickers hardness
PMMA/SF	F3	19.84	Vickers hardness
PMMA/SF	F4	19.12	Vickers hardness
PMMA/SF	Control	22.26	Vickers hardness
PMMA/SF	F1	20.94	Vickers hardness
PMMA/SF	F2	20.22	Vickers hardness
PMMA/SF	F3	19.44	Vickers hardness
PMMA/SF	F4	18.72	Vickers hardness
PMMA/SF	Control	21.86	Vickers hardness
PMMA/SF	F1	20.8	Vickers hardness
PMMA/SF	F2	20.08	Vickers hardness
PMMA/SF	F3	19.3	Vickers hardness
PMMA/SF	F4	18.58	Vickers hardness
PMMA/SF	Control	21.72	Vickers hardness
PMMA/SF	F1	21.2	Vickers hardness
PMMA/SF	F2	20.48	Vickers hardness
PMMA/SF	F3	19.7	Vickers hardness
PMMA/SF	F4	18.98	Vickers hardness
PMMA/SF	Control	22.12	Vickers hardness
PMMA/HF	F1	22.94	Vickers hardness
PMMA/HF	F2	22.08	Vickers hardness
PMMA/HF	F3	21.12	Vickers hardness
PMMA/HF	F4	20.18	Vickers hardness
PMMA/HF	Control	23.86	Vickers hardness
PMMA/HF	F1	23.06	Vickers hardness
PMMA/HF	F2	22.2	Vickers hardness
PMMA/HF	F3	21.24	Vickers hardness
PMMA/HF	F4	20.3	Vickers hardness
PMMA/HF	Control	23.98	Vickers hardness
PMMA/HF	F1	22.68	Vickers hardness
PMMA/HF	F2	21.82	Vickers hardness
PMMA/HF	F3	20.86	Vickers hardness
PMMA/HF	F4	19.92	Vickers hardness
PMMA/HF	Control	23.6	Vickers hardness
PMMA/HF	F1	23.18	Vickers hardness
PMMA/HF	F2	22.32	Vickers hardness
PMMA/HF	F3	21.36	Vickers hardness
PMMA/HF	F4	20.42	Vickers hardness
PMMA/HF	Control	24.1	Vickers hardness
PMMA/HF	F1	22.86	Vickers hardness
PMMA/HF	F2	22	Vickers hardness
PMMA/HF	F3	21.04	Vickers hardness
PMMA/HF	F4	20.1	Vickers hardness

System	Condition	Value	Outcome
PMMA/HF	Control	23.78	Vickers hardness
PMMA/HF	F1	22.98	Vickers hardness
PMMA/HF	F2	22.12	Vickers hardness
PMMA/HF	F3	21.16	Vickers hardness
PMMA/HF	F4	20.22	Vickers hardness
PMMA/HF	Control	23.9	Vickers hardness
PMMA/HF	F1	23.44	Vickers hardness
PMMA/HF	F2	22.58	Vickers hardness
PMMA/HF	F3	21.62	Vickers hardness
PMMA/HF	F4	20.68	Vickers hardness
PMMA/HF	Control	24.36	Vickers hardness
PMMA/HF	F1	23.04	Vickers hardness
PMMA/HF	F2	22.18	Vickers hardness
PMMA/HF	F3	21.22	Vickers hardness
PMMA/HF	F4	20.28	Vickers hardness
PMMA/HF	Control	23.96	Vickers hardness
PMMA/HF	F1	22.9	Vickers hardness
PMMA/HF	F2	22.04	Vickers hardness
PMMA/HF	F3	21.08	Vickers hardness
PMMA/HF	F4	20.14	Vickers hardness
PMMA/HF	Control	23.82	Vickers hardness
PMMA/HF	F1	23.3	Vickers hardness
PMMA/HF	F2	22.44	Vickers hardness
PMMA/HF	F3	21.48	Vickers hardness
PMMA/HF	F4	20.54	Vickers hardness
PMMA/HF	Control	24.22	Vickers hardness
PMMA/HF/SF	F1	25.16	Vickers hardness
PMMA/HF/SF	F2	24.22	Vickers hardness
PMMA/HF/SF	F3	23.18	Vickers hardness
PMMA/HF/SF	F4	22.12	Vickers hardness
PMMA/HF/SF	Control	26.04	Vickers hardness
PMMA/HF/SF	F1	25.28	Vickers hardness
PMMA/HF/SF	F2	24.34	Vickers hardness
PMMA/HF/SF	F3	23.3	Vickers hardness
PMMA/HF/SF	F4	22.24	Vickers hardness
PMMA/HF/SF	Control	26.16	Vickers hardness
PMMA/HF/SF	F1	24.9	Vickers hardness
PMMA/HF/SF	F2	23.96	Vickers hardness
PMMA/HF/SF	F3	22.92	Vickers hardness
PMMA/HF/SF	F4	21.86	Vickers hardness
PMMA/HF/SF	Control	25.78	Vickers hardness
PMMA/HF/SF	F1	25.4	Vickers hardness
PMMA/HF/SF	F2	24.46	Vickers hardness
PMMA/HF/SF	F3	23.42	Vickers hardness
PMMA/HF/SF	F4	22.36	Vickers hardness
PMMA/HF/SF	Control	26.28	Vickers hardness
PMMA/HF/SF	F1	25.08	Vickers hardness

System	Condition	Value	Outcome
PMMA/HF/SF	F2	24.14	Vickers hardness
PMMA/HF/SF	F3	23.1	Vickers hardness
PMMA/HF/SF	F4	22.04	Vickers hardness
PMMA/HF/SF	Control	25.96	Vickers hardness
PMMA/HF/SF	F1	25.2	Vickers hardness
PMMA/HF/SF	F2	24.26	Vickers hardness
PMMA/HF/SF	F3	23.22	Vickers hardness
PMMA/HF/SF	F4	22.16	Vickers hardness
PMMA/HF/SF	Control	26.08	Vickers hardness
PMMA/HF/SF	F1	25.66	Vickers hardness
PMMA/HF/SF	F2	24.72	Vickers hardness
PMMA/HF/SF	F3	23.68	Vickers hardness
PMMA/HF/SF	F4	22.62	Vickers hardness
PMMA/HF/SF	Control	26.54	Vickers hardness
PMMA/HF/SF	F1	25.26	Vickers hardness
PMMA/HF/SF	F2	24.32	Vickers hardness
PMMA/HF/SF	F3	23.28	Vickers hardness
PMMA/HF/SF	F4	22.22	Vickers hardness
PMMA/HF/SF	Control	26.14	Vickers hardness
PMMA/HF/SF	F1	25.12	Vickers hardness
PMMA/HF/SF	F2	24.18	Vickers hardness
PMMA/HF/SF	F3	23.14	Vickers hardness
PMMA/HF/SF	F4	22.08	Vickers hardness
PMMA/HF/SF	Control	26	Vickers hardness
PMMA/HF/SF	F1	25.52	Vickers hardness
PMMA/HF/SF	F2	24.58	Vickers hardness
PMMA/HF/SF	F3	23.54	Vickers hardness
PMMA/HF/SF	F4	22.48	Vickers hardness
PMMA/HF/SF	Control	26.4	Vickers hardness
PMMA	F1	14.12	Impact strength
PMMA	F2	13.36	Impact strength
PMMA	F3	12.52	Impact strength
PMMA	F4	11.74	Impact strength
PMMA	Control	14.96	Impact strength
PMMA	F1	14.24	Impact strength
PMMA	F2	13.48	Impact strength
PMMA	F3	12.66	Impact strength
PMMA	F4	11.86	Impact strength
PMMA	Control	15.08	Impact strength
PMMA	F1	13.88	Impact strength
PMMA	F2	13.12	Impact strength
PMMA	F3	12.28	Impact strength
PMMA	F4	11.52	Impact strength
PMMA	Control	14.72	Impact strength
PMMA	F1	14.36	Impact strength
PMMA	F2	13.58	Impact strength
PMMA	F3	12.74	Impact strength

System	Condition	Value	Outcome
PMMA	F4	11.98	Impact strength
PMMA	Control	15.22	Impact strength
PMMA	F1	14.06	Impact strength
PMMA	F2	13.3	Impact strength
PMMA	F3	12.46	Impact strength
PMMA	F4	11.68	Impact strength
PMMA	Control	14.9	Impact strength
PMMA	F1	14.14	Impact strength
PMMA	F2	13.38	Impact strength
PMMA	F3	12.54	Impact strength
PMMA	F4	11.76	Impact strength
PMMA	Control	14.98	Impact strength
PMMA	F1	14.58	Impact strength
PMMA	F2	13.82	Impact strength
PMMA	F3	12.98	Impact strength
PMMA	F4	12.2	Impact strength
PMMA	Control	15.44	Impact strength
PMMA	F1	14.2	Impact strength
PMMA	F2	13.44	Impact strength
PMMA	F3	12.6	Impact strength
PMMA	F4	11.82	Impact strength
PMMA	Control	15.04	Impact strength
PMMA	F1	14.02	Impact strength
PMMA	F2	13.26	Impact strength
PMMA	F3	12.42	Impact strength
PMMA	F4	11.64	Impact strength
PMMA	Control	14.86	Impact strength
PMMA	F1	14.42	Impact strength
PMMA	F2	13.66	Impact strength
PMMA	F3	12.82	Impact strength
PMMA	F4	12.04	Impact strength
PMMA	Control	15.28	Impact strength
PMMA/SF	F1	17.02	Impact strength
PMMA/SF	F2	16.12	Impact strength
PMMA/SF	F3	15.18	Impact strength
PMMA/SF	F4	14.26	Impact strength
PMMA/SF	Control	17.92	Impact strength
PMMA/SF	F1	17.14	Impact strength
PMMA/SF	F2	16.24	Impact strength
PMMA/SF	F3	15.32	Impact strength
PMMA/SF	F4	14.38	Impact strength
PMMA/SF	Control	18.04	Impact strength
PMMA/SF	F1	16.76	Impact strength
PMMA/SF	F2	15.88	Impact strength
PMMA/SF	F3	14.94	Impact strength
PMMA/SF	F4	14.02	Impact strength
PMMA/SF	Control	17.66	Impact strength

System	Condition	Value	Outcome
PMMA/SF	F1	17.26	Impact strength
PMMA/SF	F2	16.36	Impact strength
PMMA/SF	F3	15.42	Impact strength
PMMA/SF	F4	14.48	Impact strength
PMMA/SF	Control	18.18	Impact strength
PMMA/SF	F1	16.94	Impact strength
PMMA/SF	F2	16.04	Impact strength
PMMA/SF	F3	15.1	Impact strength
PMMA/SF	F4	14.18	Impact strength
PMMA/SF	Control	17.84	Impact strength
PMMA/SF	F1	17.06	Impact strength
PMMA/SF	F2	16.16	Impact strength
PMMA/SF	F3	15.22	Impact strength
PMMA/SF	F4	14.3	Impact strength
PMMA/SF	Control	17.96	Impact strength
PMMA/SF	F1	17.52	Impact strength
PMMA/SF	F2	16.62	Impact strength
PMMA/SF	F3	15.68	Impact strength
PMMA/SF	F4	14.74	Impact strength
PMMA/SF	Control	18.42	Impact strength
PMMA/SF	F1	17.12	Impact strength
PMMA/SF	F2	16.22	Impact strength
PMMA/SF	F3	15.28	Impact strength
PMMA/SF	F4	14.36	Impact strength
PMMA/SF	Control	18.02	Impact strength
PMMA/SF	F1	16.98	Impact strength
PMMA/SF	F2	16.08	Impact strength
PMMA/SF	F3	15.14	Impact strength
PMMA/SF	F4	14.22	Impact strength
PMMA/SF	Control	17.88	Impact strength
PMMA/SF	F1	17.38	Impact strength
PMMA/SF	F2	16.48	Impact strength
PMMA/SF	F3	15.54	Impact strength
PMMA/SF	F4	14.62	Impact strength
PMMA/SF	Control	18.28	Impact strength
PMMA/HF	F1	20.12	Impact strength
PMMA/HF	F2	19.04	Impact strength
PMMA/HF	F3	17.92	Impact strength
PMMA/HF	F4	16.84	Impact strength
PMMA/HF	Control	21.06	Impact strength
PMMA/HF	F1	20.24	Impact strength
PMMA/HF	F2	19.16	Impact strength
PMMA/HF	F3	18.04	Impact strength
PMMA/HF	F4	16.96	Impact strength
PMMA/HF	Control	21.18	Impact strength
PMMA/HF	F1	19.88	Impact strength
PMMA/HF	F2	18.8	Impact strength

System	Condition	Value	Outcome
PMMA/HF	F3	17.68	Impact strength
PMMA/HF	F4	16.6	Impact strength
PMMA/HF	Control	20.82	Impact strength
PMMA/HF	F1	20.38	Impact strength
PMMA/HF	F2	19.28	Impact strength
PMMA/HF	F3	18.16	Impact strength
PMMA/HF	F4	17.08	Impact strength
PMMA/HF	Control	21.32	Impact strength
PMMA/HF	F1	20.02	Impact strength
PMMA/HF	F2	18.94	Impact strength
PMMA/HF	F3	17.82	Impact strength
PMMA/HF	F4	16.74	Impact strength
PMMA/HF	Control	20.96	Impact strength
PMMA/HF	F1	20.14	Impact strength
PMMA/HF	F2	19.06	Impact strength
PMMA/HF	F3	17.94	Impact strength
PMMA/HF	F4	16.86	Impact strength
PMMA/HF	Control	21.08	Impact strength
PMMA/HF	F1	20.62	Impact strength
PMMA/HF	F2	19.52	Impact strength
PMMA/HF	F3	18.4	Impact strength
PMMA/HF	F4	17.32	Impact strength
PMMA/HF	Control	21.56	Impact strength
PMMA/HF	F1	20.18	Impact strength
PMMA/HF	F2	19.1	Impact strength
PMMA/HF	F3	17.98	Impact strength
PMMA/HF	F4	16.9	Impact strength
PMMA/HF	Control	21.12	Impact strength
PMMA/HF	F1	20.04	Impact strength
PMMA/HF	F2	18.96	Impact strength
PMMA/HF	F3	17.84	Impact strength
PMMA/HF	F4	16.76	Impact strength
PMMA/HF	Control	20.98	Impact strength
PMMA/HF	F1	20.46	Impact strength
PMMA/HF	F2	19.38	Impact strength
PMMA/HF	F3	18.26	Impact strength
PMMA/HF	F4	17.18	Impact strength
PMMA/HF	Control	21.4	Impact strength
PMMA/HF/SF	F1	23.04	Impact strength
PMMA/HF/SF	F2	21.92	Impact strength
PMMA/HF/SF	F3	20.78	Impact strength
PMMA/HF/SF	F4	19.62	Impact strength
PMMA/HF/SF	Control	24.12	Impact strength
PMMA/HF/SF	F1	23.16	Impact strength
PMMA/HF/SF	F2	22.04	Impact strength
PMMA/HF/SF	F3	20.9	Impact strength
PMMA/HF/SF	F4	19.74	Impact strength

System	Condition	Value	Outcome
PMMA/HF/SF	Control	24.24	Impact strength
PMMA/HF/SF	F1	22.82	Impact strength
PMMA/HF/SF	F2	21.7	Impact strength
PMMA/HF/SF	F3	20.56	Impact strength
PMMA/HF/SF	F4	19.4	Impact strength
PMMA/HF/SF	Control	23.9	Impact strength
PMMA/HF/SF	F1	23.28	Impact strength
PMMA/HF/SF	F2	22.16	Impact strength
PMMA/HF/SF	F3	21.02	Impact strength
PMMA/HF/SF	F4	19.86	Impact strength
PMMA/HF/SF	Control	24.36	Impact strength
PMMA/HF/SF	F1	22.96	Impact strength
PMMA/HF/SF	F2	21.84	Impact strength
PMMA/HF/SF	F3	20.7	Impact strength
PMMA/HF/SF	F4	19.54	Impact strength
PMMA/HF/SF	Control	24.04	Impact strength
PMMA/HF/SF	F1	23.08	Impact strength
PMMA/HF/SF	F2	21.96	Impact strength
PMMA/HF/SF	F3	20.82	Impact strength
PMMA/HF/SF	F4	19.66	Impact strength
PMMA/HF/SF	Control	24.16	Impact strength
PMMA/HF/SF	F1	23.56	Impact strength
PMMA/HF/SF	F2	22.42	Impact strength
PMMA/HF/SF	F3	21.28	Impact strength
PMMA/HF/SF	F4	20.12	Impact strength
PMMA/HF/SF	Control	24.64	Impact strength
PMMA/HF/SF	F1	23.12	Impact strength
PMMA/HF/SF	F2	22	Impact strength
PMMA/HF/SF	F3	20.86	Impact strength
PMMA/HF/SF	F4	19.7	Impact strength
PMMA/HF/SF	Control	24.2	Impact strength
PMMA/HF/SF	F1	22.98	Impact strength
PMMA/HF/SF	F2	21.86	Impact strength
PMMA/HF/SF	F3	20.72	Impact strength
PMMA/HF/SF	F4	19.56	Impact strength
PMMA/HF/SF	Control	24.06	Impact strength
PMMA/HF/SF	F1	23.42	Impact strength
PMMA/HF/SF	F2	22.3	Impact strength
PMMA/HF/SF	F3	21.16	Impact strength
PMMA/HF/SF	F4	20	Impact strength
PMMA/HF/SF	Control	24.5	Impact strength

S3. MTT bacterial metabolic viability statistical analysis

MTT-derived bacterial metabolic viability was analyzed separately for L. acidophilus and S. mutans using balanced 4 × 5 factorial models (n = 3 independent biological experiments per experimental cell).

S3.1 Assumption diagnostics

Species	Cell Shapiro p min	Cell Shapiro p max	Cells p<0.05	Residual Shapiro W	Residual p	Levene F	Levene p	Q-Q r	|Std resid|>3	Cell(s) p<0.05
L. acidophilus	0.0122	0.9284	1	0.9763	0.2938	0.2862	0.9976	0.9913	0	PMMA / Frac3 (p=0.012206)
S. mutans	0.2738	0.9845	0	0.9653	0.0857	0.2261	0.9995	0.9867	0	

S3.2 Two-way factorial ANOVA

Species	Effect	df1	df2	F	Exact p	Partial eta²
L. acidophilus	Material system	3	40	2904.2994	8.094594e-47	0.9954
L. acidophilus	Phytocannabinoid condition	4	40	43495.6336	3.558068e-72	0.9998
L. acidophilus	System × Condition	12	40	194.6712	1.638012e-31	0.9832
S. mutans	Material system	3	40	2243.0323	1.381463e-44	0.9941
S. mutans	Phytocannabinoid condition	4	40	28277.5121	1.950644e-68	0.9996
S. mutans	System × Condition	12	40	56.5976	3.248976e-21	0.9444

S3.3 Simple-effects analyses

Species	Simple effect	Level	F	Exact p
L. acidophilus	System within condition	Control	272.3191	1.210941e-26
L. acidophilus	System within condition	Frac1	1294.9212	7.478104e-40
L. acidophilus	System within condition	Frac2	551.8129	1.450787e-32
L. acidophilus	System within condition	Frac3	1160.5026	6.534986e-39
L. acidophilus	System within condition	Frac4	403.4284	6.386716e-30
L. acidophilus	Condition within system	PMMA/HF/SF	11843.5551	6.996773e-61
L. acidophilus	Condition within system	PMMA/SF	11447.0125	1.381753e-60
L. acidophilus	Condition within system	PMMA/HF	10991.2327	3.111832e-60
L. acidophilus	Condition within system	PMMA	9797.847	3.092600e-59
S. mutans	System within condition	Control	256.2554	3.848067e-26
S. mutans	System within condition	Frac1	678.4914	2.545714e-34
S. mutans	System within condition	Frac2	798.2614	1.046033e-35
S. mutans	System within condition	Frac3	248.2031	7.051819e-26
S. mutans	System within condition	Frac4	488.2112	1.577598e-31
S. mutans	Condition within system	PMMA/HF/SF	6773.3806	4.928166e-56
S. mutans	Condition within system	PMMA/SF	8020.9624	1.684039e-57
S. mutans	Condition within system	PMMA/HF	7512.6436	6.226720e-57
S. mutans	Condition within system	PMMA	6140.3181	3.496188e-55

S3.4 Tukey-adjusted pairwise comparisons

Species	Comparison family	Group A	Group B	Mean difference	Tukey-adjusted exact p	Decision
L. acidophilus	System within Control	PMMA/HF/SF	PMMA/SF	-3.9867	1.203526e-11	Significant
L. acidophilus	System within Control	PMMA/HF/SF	PMMA/HF	-6.0833	0	Significant
L. acidophilus	System within Control	PMMA/HF/SF	PMMA	-11.19	0	Significant
L. acidophilus	System within Control	PMMA/SF	PMMA/HF	-2.0967	3.062107e-05	Significant
L. acidophilus	System within Control	PMMA/SF	PMMA	-7.2033	0	Significant
L. acidophilus	System within Control	PMMA/HF	PMMA	-5.1067	5.662137e-15	Significant
L. acidophilus	System within Frac1	PMMA/HF/SF	PMMA/SF	-0.69	0.3229	Not significant
L. acidophilus	System within Frac1	PMMA/HF/SF	PMMA/HF	-3.0467	1.494874e-08	Significant
L. acidophilus	System within Frac1	PMMA/HF/SF	PMMA	-21.3967	0	Significant
L. acidophilus	System within Frac1	PMMA/SF	PMMA/HF	-2.3567	3.789028e-06	Significant
L. acidophilus	System within Frac1	PMMA/SF	PMMA	-20.7067	0	Significant
L. acidophilus	System within Frac1	PMMA/HF	PMMA	-18.35	0	Significant
L. acidophilus	System within Frac2	PMMA/HF/SF	PMMA/SF	-7.07	0	Significant
L. acidophilus	System within Frac2	PMMA/HF/SF	PMMA/HF	-11.0867	0	Significant
L. acidophilus	System within Frac2	PMMA/HF/SF	PMMA	-15.64	0	Significant
L. acidophilus	System within Frac2	PMMA/SF	PMMA/HF	-4.0167	9.693690e-12	Significant
L. acidophilus	System within Frac2	PMMA/SF	PMMA	-8.57	0	Significant
L. acidophilus	System within Frac2	PMMA/HF	PMMA	-4.5533	2.281508e-13	Significant
L. acidophilus	System within Frac3	PMMA/HF/SF	PMMA/SF	-17.1267	0	Significant
L. acidophilus	System within Frac3	PMMA/HF/SF	PMMA/HF	-19.15	0	Significant
L. acidophilus	System within Frac3	PMMA/HF/SF	PMMA	-20.7433	0	Significant
L. acidophilus	System within Frac3	PMMA/SF	PMMA/HF	-2.0233	5.488869e-05	Significant
L. acidophilus	System within Frac3	PMMA/SF	PMMA	-3.6167	1.840269e-10	Significant
L. acidophilus	System within Frac3	PMMA/HF	PMMA	-1.5933	0.001503	Significant
L. acidophilus	System within Frac4	PMMA/HF/SF	PMMA/SF	-3.0667	1.276669e-08	Significant
L. acidophilus	System within Frac4	PMMA/HF/SF	PMMA/HF	-8.0967	0	Significant
L. acidophilus	System within Frac4	PMMA/HF/SF	PMMA	-12.89	0	Significant
L. acidophilus	System within Frac4	PMMA/SF	PMMA/HF	-5.03	9.436896e-15	Significant
L. acidophilus	System within Frac4	PMMA/SF	PMMA	-9.8233	0	Significant
L. acidophilus	System within Frac4	PMMA/HF	PMMA	-4.7933	4.563017e-14	Significant
L. acidophilus	Condition within PMMA/HF/SF	Control	Frac1	70.8433	4.440892e-16	Significant
L. acidophilus	Condition within PMMA/HF/SF	Control	Frac2	70.2133	4.440892e-16	Significant
L. acidophilus	Condition within PMMA/HF/SF	Control	Frac3	67.1267	4.440892e-16	Significant
L. acidophilus	Condition within PMMA/HF/SF	Control	Frac4	66.0367	4.440892e-16	Significant
L. acidophilus	Condition within PMMA/HF/SF	Frac1	Frac2	-0.63	0.5198	Not significant
L. acidophilus	Condition within PMMA/HF/SF	Frac1	Frac3	-3.7167	1.447377e-10	Significant
L. acidophilus	Condition within PMMA/HF/SF	Frac1	Frac4	-4.8067	7.072121e-14	Significant
L. acidophilus	Condition within PMMA/HF/SF	Frac2	Frac3	-3.0867	1.807667e-08	Significant
L. acidophilus	Condition within PMMA/HF/SF	Frac2	Frac4	-4.1767	5.145884e-12	Significant
L. acidophilus	Condition within PMMA/HF/SF	Frac3	Frac4	-1.09	0.067	Not significant
L. acidophilus	Condition within PMMA/SF	Control	Frac1	74.14	4.440892e-16	Significant
L. acidophilus	Condition within PMMA/SF	Control	Frac2	67.13	4.440892e-16	Significant
L. acidophilus	Condition within PMMA/SF	Control	Frac3	53.9867	4.440892e-16	Significant
L. acidophilus	Condition within PMMA/SF	Control	Frac4	66.9567	4.440892e-16	Significant
L. acidophilus	Condition within PMMA/SF	Frac1	Frac2	-7.01	4.440892e-16	Significant
L. acidophilus	Condition within PMMA/SF	Frac1	Frac3	-20.1533	4.440892e-16	Significant

Species	Comparison family	Group A	Group B	Mean difference	Tukey-adjusted exact p	Decision
L. acidophilus	Condition within PMMA/SF	Frac1	Frac4	-7.1833	4.440892e-16	Significant
L. acidophilus	Condition within PMMA/SF	Frac2	Frac3	-13.1433	4.440892e-16	Significant
L. acidophilus	Condition within PMMA/SF	Frac2	Frac4	-0.1733	0.9923	Not significant
L. acidophilus	Condition within PMMA/SF	Frac3	Frac4	12.97	4.440892e-16	Significant
L. acidophilus	Condition within PMMA/HF	Control	Frac1	73.88	4.440892e-16	Significant
L. acidophilus	Condition within PMMA/HF	Control	Frac2	65.21	4.440892e-16	Significant
L. acidophilus	Condition within PMMA/HF	Control	Frac3	54.06	4.440892e-16	Significant
L. acidophilus	Condition within PMMA/HF	Control	Frac4	64.0233	4.440892e-16	Significant
L. acidophilus	Condition within PMMA/HF	Frac1	Frac2	-8.67	4.440892e-16	Significant
L. acidophilus	Condition within PMMA/HF	Frac1	Frac3	-19.82	4.440892e-16	Significant
L. acidophilus	Condition within PMMA/HF	Frac1	Frac4	-9.8567	4.440892e-16	Significant
L. acidophilus	Condition within PMMA/HF	Frac2	Frac3	-11.15	4.440892e-16	Significant
L. acidophilus	Condition within PMMA/HF	Frac2	Frac4	-1.1867	0.0378	Significant
L. acidophilus	Condition within PMMA/HF	Frac3	Frac4	9.9633	4.440892e-16	Significant
L. acidophilus	Condition within PMMA	Control	Frac1	60.6367	4.440892e-16	Significant
L. acidophilus	Condition within PMMA	Control	Frac2	65.7633	4.440892e-16	Significant
L. acidophilus	Condition within PMMA	Control	Frac3	57.5733	4.440892e-16	Significant
L. acidophilus	Condition within PMMA	Control	Frac4	64.3367	4.440892e-16	Significant
L. acidophilus	Condition within PMMA	Frac1	Frac2	5.1267	9.436896e-15	Significant
L. acidophilus	Condition within PMMA	Frac1	Frac3	-3.0633	2.172258e-08	Significant
L. acidophilus	Condition within PMMA	Frac1	Frac4	3.7	1.638510e-10	Significant
L. acidophilus	Condition within PMMA	Frac2	Frac3	-8.19	4.440892e-16	Significant
L. acidophilus	Condition within PMMA	Frac2	Frac4	-1.4267	0.007889	Significant
L. acidophilus	Condition within PMMA	Frac3	Frac4	6.7633	4.440892e-16	Significant
S. mutans	System within Control	PMMA/HF/SF	PMMA/SF	-8.2	0	Significant
S. mutans	System within Control	PMMA/HF/SF	PMMA/HF	-8.3833	0	Significant
S. mutans	System within Control	PMMA/HF/SF	PMMA	-13.7467	0	Significant
S. mutans	System within Control	PMMA/SF	PMMA/HF	-0.1833	0.983	Not significant
S. mutans	System within Control	PMMA/SF	PMMA	-5.5467	5.681011e-13	Significant
S. mutans	System within Control	PMMA/HF	PMMA	-5.3633	1.565303e-12	Significant
S. mutans	System within Frac1	PMMA/HF/SF	PMMA/SF	-3.8167	1.560999e-08	Significant
S. mutans	System within Frac1	PMMA/HF/SF	PMMA/HF	-4.56	1.621958e-10	Significant
S. mutans	System within Frac1	PMMA/HF/SF	PMMA	-20.8133	0	Significant
S. mutans	System within Frac1	PMMA/SF	PMMA/HF	-0.7433	0.4567	Not significant
S. mutans	System within Frac1	PMMA/SF	PMMA	-16.9967	0	Significant
S. mutans	System within Frac1	PMMA/HF	PMMA	-16.2533	0	Significant
S. mutans	System within Frac2	PMMA/HF/SF	PMMA/SF	-2.1933	0.000471	Significant
S. mutans	System within Frac2	PMMA/HF/SF	PMMA/HF	-5.5333	6.113998e-13	Significant
S. mutans	System within Frac2	PMMA/HF/SF	PMMA	-22.0733	0	Significant
S. mutans	System within Frac2	PMMA/SF	PMMA/HF	-3.34	3.238988e-07	Significant
S. mutans	System within Frac2	PMMA/SF	PMMA	-19.88	0	Significant
S. mutans	System within Frac2	PMMA/HF	PMMA	-16.54	0	Significant
S. mutans	System within Frac3	PMMA/HF/SF	PMMA/SF	-2.2867	0.000266	Significant
S. mutans	System within Frac3	PMMA/HF/SF	PMMA/HF	-3.5533	8.276641e-08	Significant
S. mutans	System within Frac3	PMMA/HF/SF	PMMA	-12.7167	0	Significant
S. mutans	System within Frac3	PMMA/SF	PMMA/HF	-1.2667	0.0707	Not significant
S. mutans	System within Frac3	PMMA/SF	PMMA	-10.43	0	Significant

Species	Comparison family	Group A	Group B	Mean difference	Tukey-adjusted exact p	Decision
S. mutans	System within Frac3	PMMA/HF	PMMA	-9.1633	0	Significant
S. mutans	System within Frac4	PMMA/HF/SF	PMMA/SF	-1.9633	0.001859	Significant
S. mutans	System within Frac4	PMMA/HF/SF	PMMA/HF	-5.98	5.517808e-14	Significant
S. mutans	System within Frac4	PMMA/HF/SF	PMMA	-17.4933	0	Significant
S. mutans	System within Frac4	PMMA/SF	PMMA/HF	-4.0167	4.466145e-09	Significant
S. mutans	System within Frac4	PMMA/SF	PMMA	-15.53	0	Significant
S. mutans	System within Frac4	PMMA/HF	PMMA	-11.5133	0	Significant
S. mutans	Condition within PMMA/HF/SF	Control	Frac1	60.26	4.440892e-16	Significant
S. mutans	Condition within PMMA/HF/SF	Control	Frac2	61.82	4.440892e-16	Significant
S. mutans	Condition within PMMA/HF/SF	Control	Frac3	67.4333	4.440892e-16	Significant
S. mutans	Condition within PMMA/HF/SF	Control	Frac4	69.1733	4.440892e-16	Significant
S. mutans	Condition within PMMA/HF/SF	Frac1	Frac2	1.56	0.0267	Significant
S. mutans	Condition within PMMA/HF/SF	Frac1	Frac3	7.1733	6.661338e-16	Significant
S. mutans	Condition within PMMA/HF/SF	Frac1	Frac4	8.9133	4.440892e-16	Significant
S. mutans	Condition within PMMA/HF/SF	Frac2	Frac3	5.6133	6.576961e-13	Significant
S. mutans	Condition within PMMA/HF/SF	Frac2	Frac4	7.3533	5.551115e-16	Significant
S. mutans	Condition within PMMA/HF/SF	Frac3	Frac4	1.74	0.0104	Significant
S. mutans	Condition within PMMA/SF	Control	Frac1	64.6433	4.440892e-16	Significant
S. mutans	Condition within PMMA/SF	Control	Frac2	67.8267	4.440892e-16	Significant
S. mutans	Condition within PMMA/SF	Control	Frac3	73.3467	4.440892e-16	Significant
S. mutans	Condition within PMMA/SF	Control	Frac4	75.41	4.440892e-16	Significant
S. mutans	Condition within PMMA/SF	Frac1	Frac2	3.1833	1.462977e-06	Significant
S. mutans	Condition within PMMA/SF	Frac1	Frac3	8.7033	4.440892e-16	Significant
S. mutans	Condition within PMMA/SF	Frac1	Frac4	10.7667	4.440892e-16	Significant
S. mutans	Condition within PMMA/SF	Frac2	Frac3	5.52	1.095013e-12	Significant
S. mutans	Condition within PMMA/SF	Frac2	Frac4	7.5833	4.440892e-16	Significant
S. mutans	Condition within PMMA/SF	Frac3	Frac4	2.0633	0.001658	Significant
S. mutans	Condition within PMMA/HF	Control	Frac1	64.0833	4.440892e-16	Significant
S. mutans	Condition within PMMA/HF	Control	Frac2	64.67	4.440892e-16	Significant
S. mutans	Condition within PMMA/HF	Control	Frac3	72.2633	4.440892e-16	Significant
S. mutans	Condition within PMMA/HF	Control	Frac4	71.5767	4.440892e-16	Significant
S. mutans	Condition within PMMA/HF	Frac1	Frac2	0.5867	0.7677	Not significant
S. mutans	Condition within PMMA/HF	Frac1	Frac3	8.18	4.440892e-16	Significant
S. mutans	Condition within PMMA/HF	Frac1	Frac4	7.4933	4.440892e-16	Significant
S. mutans	Condition within PMMA/HF	Frac2	Frac3	7.5933	4.440892e-16	Significant
S. mutans	Condition within PMMA/HF	Frac2	Frac4	6.9067	1.332268e-15	Significant
S. mutans	Condition within PMMA/HF	Frac3	Frac4	-0.6867	0.6497	Not significant
S. mutans	Condition within PMMA	Control	Frac1	53.1933	4.440892e-16	Significant
S. mutans	Condition within PMMA	Control	Frac2	53.4933	4.440892e-16	Significant
S. mutans	Condition within PMMA	Control	Frac3	68.4633	4.440892e-16	Significant
S. mutans	Condition within PMMA	Control	Frac4	65.4267	4.440892e-16	Significant
S. mutans	Condition within PMMA	Frac1	Frac2	0.3	0.9745	Not significant
S. mutans	Condition within PMMA	Frac1	Frac3	15.27	4.440892e-16	Significant
S. mutans	Condition within PMMA	Frac1	Frac4	12.2333	4.440892e-16	Significant
S. mutans	Condition within PMMA	Frac2	Frac3	14.97	4.440892e-16	Significant
S. mutans	Condition within PMMA	Frac2	Frac4	11.9333	4.440892e-16	Significant
S. mutans	Condition within PMMA	Frac3	Frac4	-3.0367	3.754441e-06	Significant

S3.5 Descriptive statistics

Species	Material system	Condition	Mean viability (%)	SD	n
L. acidophilus	PMMA	Control	96.6267	0.402	3
L. acidophilus	PMMA	Frac1	35.99	0.7562	3
L. acidophilus	PMMA	Frac2	30.8633	0.5294	3
L. acidophilus	PMMA	Frac3	39.0533	0.7823	3
L. acidophilus	PMMA	Frac4	32.29	0.5142	3
L. acidophilus	PMMA/HF	Control	91.52	0.4386	3
L. acidophilus	PMMA/HF	Frac1	17.64	0.2594	3
L. acidophilus	PMMA/HF	Frac2	26.31	0.4257	3
L. acidophilus	PMMA/HF	Frac3	37.46	0.4158	3
L. acidophilus	PMMA/HF	Frac4	27.4967	0.3669	3
L. acidophilus	PMMA/HF/SF	Control	85.4367	0.6616	3
L. acidophilus	PMMA/HF/SF	Frac1	14.5933	0.3927	3
L. acidophilus	PMMA/HF/SF	Frac2	15.2233	0.2641	3
L. acidophilus	PMMA/HF/SF	Frac3	18.31	0.3242	3
L. acidophilus	PMMA/HF/SF	Frac4	19.4	0.4877	3
L. acidophilus	PMMA/SF	Control	89.4233	0.8334	3
L. acidophilus	PMMA/SF	Frac1	15.2833	0.2303	3
L. acidophilus	PMMA/SF	Frac2	22.2933	0.3252	3
L. acidophilus	PMMA/SF	Frac3	35.4367	0.3707	3
L. acidophilus	PMMA/SF	Frac4	22.4667	0.3853	3
S. mutans	PMMA	Control	98.1333	0.6906	3
S. mutans	PMMA	Frac1	44.94	0.6646	3
S. mutans	PMMA	Frac2	44.64	0.5467	3
S. mutans	PMMA	Frac3	29.67	0.7519	3
S. mutans	PMMA	Frac4	32.7067	0.6062	3
S. mutans	PMMA/HF	Control	92.77	0.6751	3
S. mutans	PMMA/HF	Frac1	28.6867	0.5316	3
S. mutans	PMMA/HF	Frac2	28.1	0.6255	3
S. mutans	PMMA/HF	Frac3	20.5067	0.6752	3
S. mutans	PMMA/HF	Frac4	21.1933	0.4196	3
S. mutans	PMMA/HF/SF	Control	84.3867	0.7229	3
S. mutans	PMMA/HF/SF	Frac1	24.1267	0.38	3
S. mutans	PMMA/HF/SF	Frac2	22.5667	0.5316	3
S. mutans	PMMA/HF/SF	Frac3	16.9533	0.575	3
S. mutans	PMMA/HF/SF	Frac4	15.2133	0.355	3
S. mutans	PMMA/SF	Control	92.5867	1.0867	3
S. mutans	PMMA/SF	Frac1	27.9433	0.4775	3
S. mutans	PMMA/SF	Frac2	24.76	0.4681	3
S. mutans	PMMA/SF	Frac3	19.24	0.42	3

Species	Material system	Condition	Mean viability (%)	SD	n
S. mutans	PMMA/SF	Frac4	17.1767	0.6404	3

S3.6 Table 5 raw-data check

Species	Material system	Condition	Raw-data mean	Current Table 5	Difference	Correct rounded mean
L. acidophilus	PMMA/HF/SF	Frac1	14.5933	14.59	0.003333	14.59
L. acidophilus	PMMA/HF/SF	Frac2	15.2233	15.22	0.003333	15.22
L. acidophilus	PMMA/HF/SF	Frac3	18.31	18.31	0	18.31
L. acidophilus	PMMA/HF/SF	Frac4	19.4	19.4	3.552714e-15	19.4
L. acidophilus	PMMA/HF/SF	Control	85.4367	85.43	0.006667	85.44
L. acidophilus	PMMA/SF	Frac1	15.2833	15.28	0.003333	15.28
L. acidophilus	PMMA/SF	Frac2	22.2933	22.29	0.003333	22.29
L. acidophilus	PMMA/SF	Frac3	35.4367	35.43	0.006667	35.44
L. acidophilus	PMMA/SF	Frac4	22.4667	22.46	0.006667	22.47
L. acidophilus	PMMA/SF	Control	89.4233	89.42	0.003333	89.42
L. acidophilus	PMMA/HF	Frac1	17.64	17.64	-3.552714e-15	17.64
L. acidophilus	PMMA/HF	Frac2	26.31	26.31	3.552714e-15	26.31
L. acidophilus	PMMA/HF	Frac3	37.46	37.46	0	37.46
L. acidophilus	PMMA/HF	Frac4	27.4967	27.49	0.006667	27.5
L. acidophilus	PMMA/HF	Control	91.52	91.52	0	91.52
L. acidophilus	PMMA	Frac1	35.99	35.99	0	35.99
L. acidophilus	PMMA	Frac2	30.8633	30.86	0.003333	30.86
L. acidophilus	PMMA	Frac3	39.0533	39.05	0.003333	39.05
L. acidophilus	PMMA	Frac4	32.29	32.29	0	32.29
L. acidophilus	PMMA	Control	96.6267	96.6	0.0267	96.63
S. mutans	PMMA/HF/SF	Frac1	24.1267	24.13	-0.003333	24.13
S. mutans	PMMA/HF/SF	Frac2	22.5667	22.57	-0.003333	22.57
S. mutans	PMMA/HF/SF	Frac3	16.9533	16.95	0.003333	16.95
S. mutans	PMMA/HF/SF	Frac4	15.2133	15.21	0.003333	15.21
S. mutans	PMMA/HF/SF	Control	84.3867	84.39	-0.003333	84.39
S. mutans	PMMA/SF	Frac1	27.9433	27.94	0.003333	27.94
S. mutans	PMMA/SF	Frac2	24.76	24.76	0	24.76
S. mutans	PMMA/SF	Frac3	19.24	19.24	3.552714e-15	19.24
S. mutans	PMMA/SF	Frac4	17.1767	17.18	-0.003333	17.18
S. mutans	PMMA/SF	Control	92.5867	92.59	-0.003333	92.59
S. mutans	PMMA/HF	Frac1	28.6867	28.69	-0.003333	28.69
S. mutans	PMMA/HF	Frac2	28.1	28.1	-3.552714e-15	28.1
S. mutans	PMMA/HF	Frac3	20.5067	20.51	-0.003333	20.51
S. mutans	PMMA/HF	Frac4	21.1933	21.19	0.003333	21.19
S. mutans	PMMA/HF	Control	92.77	92.77	0	92.77
S. mutans	PMMA	Frac1	44.94	44.94	0	44.94
S. mutans	PMMA	Frac2	44.64	44.64	-7.105427e-15	44.64

Species	Material system	Condition	Raw-data mean	Current Table 5	Difference	Correct rounded mean
S. mutans	PMMA	Frac3	29.67	29.67	0	29.67
S. mutans	PMMA	Frac4	32.7067	32.71	-0.003333	32.71
S. mutans	PMMA	Control	98.1333	98.13	0.003333	98.13

S3.7 Raw data – L. acidophilus

Group code	Condition	PMMA/HF/SF	PMMA/SF	PMMA/HF	PMMA
1	Frac1	14.54	15.48	17.43	36.77
1	Frac1	15.01	15.34	17.56	35.26
1	Frac1	14.23	15.03	17.93	35.94
2	Frac2	15.51	22.58	26.23	30.26
2	Frac2	14.99	21.94	26.77	31.25
2	Frac2	15.17	22.36	25.93	31.08
3	Frac3	18.25	35.17	37.01	39.5
3	Frac3	18.02	35.86	37.54	39.51
3	Frac3	18.66	35.28	37.83	38.15
4	Frac4	19.83	22.45	27.84	32.11
4	Frac4	18.87	22.09	27.11	32.87
4	Frac4	19.5	22.86	27.54	31.89
5	Control	85.17	89.56	91.42	96.25
5	Control	86.19	90.18	92	97.05
5	Control	84.95	88.53	91.14	96.58

S3.8 Raw data – S. mutans

Group code	Condition	PMMA/HF/SF	PMMA/SF	PMMA/HF	PMMA
1	Frac1	24.12	28.39	29.26	45.66
1	Frac1	23.75	27.44	28.21	44.35
1	Frac1	24.51	28	28.59	44.81
2	Frac2	22.09	24.86	28.26	44.07
2	Frac2	22.47	25.17	27.41	45.16
2	Frac2	23.14	24.25	28.63	44.69
3	Frac3	17.58	19.42	20.49	29.44
3	Frac3	16.83	18.76	21.19	29.06
3	Frac3	16.45	19.54	19.84	30.51
4	Frac4	15.21	17.15	21.03	32.75
4	Frac4	14.86	16.55	21.67	33.29
4	Frac4	15.57	17.83	20.88	32.08
5	Control	84.51	92.77	92.28	97.46
5	Control	85.04	91.42	93.54	98.1
5	Control	83.61	93.57	92.49	98.84

S4. LDH-derived cytotoxicity statistical analysis

LDH-derived cytotoxicity was analyzed using the balanced 4 × 5 factorial dataset (n = 3 independent biological experiments per experimental cell). The residual-normality departure and variance-homogeneity result are retained explicitly in the diagnostic table.

S4.1 Assumption diagnostics

Diagnostic	Statistic	df	Exact p-value	Interpretation
Shapiro–Wilk test of factorial-model residuals	0.8976		0.000108	Significant departure from normality (p < 0.05)
Levene test across 20 cells	0.3053	19, 40	0.9964	Homogeneity of variance supported

S4.2 Two-way factorial ANOVA

Effect	Sum of squares	df	Error df	F	Exact p-value	Partial eta squared
Material system	5.4716	3	40	7.6713	0.000365	0.3652
Phytocannabinoid condition	44.1176	4	40	46.3898	1.651443e-14	0.8227
Material system × condition	11.7334	12	40	4.1126	0.00035	0.5523

S4.3 Simple-effects analyses

Simple effect tested	Condition / material system	df	Error df	F	Exact p-value
Material system	Frac1	3	8	2.53	0.1307
Material system	Frac2	3	8	6.3961	0.0161
Material system	Frac3	3	8	3.5714	0.0666
Material system	Frac4	3	8	1.1972	0.3709
Material system	Control	3	8	28.7807	0.000123
Condition	PMMA/HF/SF	4	10	22.9668	5.022386e-05
Condition	PMMA/SF	4	10	10.7856	0.001194
Condition	PMMA/HF	4	10	20.9768	7.486777e-05
Condition	PMMA	4	10	6.3585	0.008215

S4.4 Tukey-adjusted pairwise comparisons

Family	Condition / material system	Group 1	Group 2	Mean difference	Tukey adjusted p	95% CI lower	95% CI upper	Significant
Material system	Frac1	PMMA	PMMA/HF	-0.6	0.2349	-1.5195	0.3195	No
Material system	Frac1	PMMA	PMMA/HF/SF	-0.71	0.1396	-1.6295	0.2095	No
Material system	Frac1	PMMA	PMMA/SF	-0.27	0.785	-1.1895	0.6495	No
Material system	Frac1	PMMA/HF	PMMA/HF/SF	-0.11	0.9796	-1.0295	0.8095	No
Material system	Frac1	PMMA/HF	PMMA/SF	0.33	0.672	-0.5895	1.2495	No
Material system	Frac1	PMMA/HF/SF	PMMA/SF	0.44	0.4635	-0.4795	1.3595	No
Material system	Frac2	PMMA	PMMA/HF	-0.46	0.7047	-1.8109	0.8909	No
Material system	Frac2	PMMA	PMMA/HF/SF	1.06	0.132	-0.2909	2.4109	No
Material system	Frac2	PMMA	PMMA/SF	1.01	0.1556	-0.3409	2.3609	No
Material system	Frac2	PMMA/HF	PMMA/HF/SF	1.52	0.0286	0.1691	2.8709	Yes
Material system	Frac2	PMMA/HF	PMMA/SF	1.47	0.0337	0.1191	2.8209	Yes

Family	Condition / material system	Group 1	Group 2	Mean difference	Tukey adjusted p	95% CI lower	95% CI upper	Significant
Material system	Frac2	PMMA/HF/SF	PMMA/SF	-0.05	0.9994	-1.4009	1.3009	No
Material system	Frac3	PMMA	PMMA/HF	-1.54	0.1	-3.359	0.279	No
Material system	Frac3	PMMA	PMMA/HF/SF	0	1	-1.819	1.819	No
Material system	Frac3	PMMA	PMMA/SF	-0.07	0.9993	-1.889	1.749	No
Material system	Frac3	PMMA/HF	PMMA/HF/SF	1.54	0.1	-0.279	3.359	No
Material system	Frac3	PMMA/HF	PMMA/SF	1.47	0.1189	-0.349	3.289	No
Material system	Frac3	PMMA/HF/SF	PMMA/SF	-0.07	0.9993	-1.889	1.749	No
Material system	Frac4	PMMA	PMMA/HF	0.74	0.3431	-0.5825	2.0625	No
Material system	Frac4	PMMA	PMMA/HF/SF	0.28	0.9026	-1.0425	1.6025	No
Material system	Frac4	PMMA	PMMA/SF	0.53	0.597	-0.7925	1.8525	No
Material system	Frac4	PMMA/HF	PMMA/HF/SF	-0.46	0.6918	-1.7825	0.8625	No
Material system	Frac4	PMMA/HF	PMMA/SF	-0.21	0.9548	-1.5325	1.1125	No
Material system	Frac4	PMMA/HF/SF	PMMA/SF	0.25	0.9275	-1.0725	1.5725	No
Material system	Control	PMMA	PMMA/HF	-1.33	0.0007	-1.9617	-0.6983	Yes
Material system	Control	PMMA	PMMA/HF/SF	-1.54	0.0002	-2.1717	-0.9083	Yes
Material system	Control	PMMA	PMMA/SF	-0.34	0.3725	-0.9717	0.2917	No
Material system	Control	PMMA/HF	PMMA/HF/SF	-0.21	0.719	-0.8417	0.4217	No
Material system	Control	PMMA/HF	PMMA/SF	0.99	0.0045	0.3583	1.6217	Yes
Material system	Control	PMMA/HF/SF	PMMA/SF	1.2	0.0013	0.5683	1.8317	Yes
Condition	PMMA/HF/SF	Control	Frac1	1.26	0.0611	-0.0517	2.5717	No
Condition	PMMA/HF/SF	Control	Frac2	2.49	0.0007	1.1783	3.8017	Yes
Condition	PMMA/HF/SF	Control	Frac3	3.26	0.0001	1.9483	4.5717	Yes
Condition	PMMA/HF/SF	Control	Frac4	2.96	0.0002	1.6483	4.2717	Yes
Condition	PMMA/HF/SF	Frac1	Frac2	1.23	0.0687	-0.0817	2.5417	No
Condition	PMMA/HF/SF	Frac1	Frac3	2	0.0037	0.6883	3.3117	Yes
Condition	PMMA/HF/SF	Frac1	Frac4	1.7	0.0112	0.3883	3.0117	Yes
Condition	PMMA/HF/SF	Frac2	Frac3	0.77	0.3614	-0.5417	2.0817	No
Condition	PMMA/HF/SF	Frac2	Frac4	0.47	0.7627	-0.8417	1.7817	No
Condition	PMMA/HF/SF	Frac3	Frac4	-0.3	0.9385	-1.6117	1.0117	No
Condition	PMMA/SF	Control	Frac1	0.5	0.6981	-0.7672	1.7672	No
Condition	PMMA/SF	Control	Frac2	1.24	0.0558	-0.0272	2.5072	No
Condition	PMMA/SF	Control	Frac3	1.99	0.003	0.7228	3.2572	Yes
Condition	PMMA/SF	Control	Frac4	2.01	0.0028	0.7428	3.2772	Yes
Condition	PMMA/SF	Frac1	Frac2	0.74	0.366	-0.5272	2.0072	No
Condition	PMMA/SF	Frac1	Frac3	1.49	0.0204	0.2228	2.7572	Yes
Condition	PMMA/SF	Frac1	Frac4	1.51	0.0188	0.2428	2.7772	Yes
Condition	PMMA/SF	Frac2	Frac3	0.75	0.3543	-0.5172	2.0172	No
Condition	PMMA/SF	Frac2	Frac4	0.77	0.3317	-0.4972	2.0372	No
Condition	PMMA/SF	Frac3	Frac4	0.02	1	-1.2472	1.2872	No
Condition	PMMA/HF	Control	Frac1	1.16	0.0622	-0.052	2.372	No
Condition	PMMA/HF	Control	Frac2	0.76	0.3054	-0.452	1.972	No
Condition	PMMA/HF	Control	Frac3	1.51	0.0143	0.298	2.722	Yes
Condition	PMMA/HF	Control	Frac4	3.21	0	1.998	4.422	Yes
Condition	PMMA/HF	Frac1	Frac2	-0.4	0.8097	-1.612	0.812	No
Condition	PMMA/HF	Frac1	Frac3	0.35	0.8707	-0.862	1.562	No
Condition	PMMA/HF	Frac1	Frac4	2.05	0.0017	0.838	3.262	Yes
Condition	PMMA/HF	Frac2	Frac3	0.75	0.3163	-0.462	1.962	No

Family	Condition / material system	Group 1	Group 2	Mean difference	Tukey adjusted p	95% CI lower	95% CI upper	Significant
Condition	PMMA/HF	Frac2	Frac4	2.45	0.0004	1.238	3.662	Yes
Condition	PMMA/HF	Frac3	Frac4	1.7	0.0066	0.488	2.912	Yes
Condition	PMMA	Control	Frac1	0.43	0.857	-1.0094	1.8694	No
Condition	PMMA	Control	Frac2	-0.11	0.999	-1.5494	1.3294	No
Condition	PMMA	Control	Frac3	1.72	0.0185	0.2806	3.1594	Yes
Condition	PMMA	Control	Frac4	1.14	0.1423	-0.2994	2.5794	No
Condition	PMMA	Frac1	Frac2	-0.54	0.7331	-1.9794	0.8994	No
Condition	PMMA	Frac1	Frac3	1.29	0.0847	-0.1494	2.7294	No
Condition	PMMA	Frac1	Frac4	0.71	0.5161	-0.7294	2.1494	No
Condition	PMMA	Frac2	Frac3	1.83	0.0126	0.3906	3.2694	Yes
Condition	PMMA	Frac2	Frac4	1.25	0.0975	-0.1894	2.6894	No
Condition	PMMA	Frac3	Frac4	-0.58	0.6827	-2.0194	0.8594	No

S4.5 Descriptive statistics

Material system	Condition	Mean	SD	n
PMMA	Control	2.85	0.27	3
PMMA	Frac1	3.28	0.56	3
PMMA	Frac2	2.74	0.28	3
PMMA	Frac3	4.57	0.81	3
PMMA	Frac4	3.99	0.56	3
PMMA/HF	Control	1.52	0.29	3
PMMA/HF	Frac1	2.68	0.21	3
PMMA/HF	Frac2	2.28	0.32	3
PMMA/HF	Frac3	3.03	0.77	3
PMMA/HF	Frac4	4.73	0.44	3
PMMA/HF/SF	Control	1.31	0.18	3
PMMA/HF/SF	Frac1	2.57	0.23	3
PMMA/HF/SF	Frac2	3.8	0.7	3
PMMA/HF/SF	Frac3	4.57	0.55	3
PMMA/HF/SF	Frac4	4.27	0.56	3
PMMA/SF	Control	2.51	0.21	3
PMMA/SF	Frac1	3.01	0.29	3
PMMA/SF	Frac2	3.75	0.63	3
PMMA/SF	Frac3	4.5	0.62	3
PMMA/SF	Frac4	4.52	0.45	3

S4.6 Revised Table 7 grouping output

Material system	Frac1	Frac2	Frac3	Frac4	Control
PMMA/HF/SF	2.57 ± 0.23 Abc	3.80 ± 0.70 Bac	4.57 ± 0.55 Aa	4.27 ± 0.56 Aa	1.31 ± 0.18 Bb
PMMA/SF	3.01 ± 0.29 Ab	3.75 ± 0.63 Bab	4.50 ± 0.62 Aa	4.52 ± 0.45 Aa	2.51 ± 0.21 Ab
PMMA/HF	2.68 ± 0.21 Abc	2.28 ± 0.32 Abc	3.03 ± 0.77 Ac	4.73 ± 0.44 Aa	1.52 ± 0.29 Bb

Material system	Frac1	Frac2	Frac3	Frac4	Control
PMMA	3.28 ± 0.56 Aab	2.74 ± 0.28 ABb	4.57 ± 0.81 Aa	3.99 ± 0.56 Aab	2.85 ± 0.27 Ab

S4.7 Raw LDH data

Material system	Condition	Veri 1	Veri 2	Veri 3
PMMA/HF/SF	Frac1	2.7398	2.662	2.3082
PMMA/HF/SF	Frac2	4.3167	4.08	3.0033
PMMA/HF/SF	Frac3	4.9759	4.79	3.9441
PMMA/HF/SF	Frac4	4.6833	4.494	3.6327
PMMA/HF/SF	Control	1.4429	1.382	1.1051
PMMA/SF	Frac1	3.224	3.126	2.68
PMMA/SF	Frac2	4.215	4.002	3.033
PMMA/SF	Frac3	4.9576	4.748	3.7944
PMMA/SF	Frac4	4.8521	4.7	4.0079
PMMA/SF	Control	2.665	2.594	2.271
PMMA/HF	Frac1	2.835	2.764	2.441
PMMA/HF	Frac2	2.5162	2.408	1.9158
PMMA/HF	Frac3	3.5983	3.338	2.1537
PMMA/HF	Frac4	5.0548	4.906	4.2292
PMMA/HF	Control	1.734	1.636	1.19
PMMA	Frac1	3.6933	3.504	2.6427
PMMA	Frac2	2.9467	2.852	2.4213
PMMA	Frac3	5.1678	4.894	3.6482
PMMA	Frac4	4.4033	4.214	3.3527
PMMA	Control	3.0493	2.958	2.5427

S4.8 Analysis notes

Item	Details
Source	LDH istatistik(1).xlsx supplied by the user; only these raw values were used.
Design	4 material systems × 5 conditions × 3 independent biological experiments = 60 observations.
Coding	Conditions: Frac1, Frac2, Frac3, Frac4, Control.
Assumption finding	Residual Shapiro–Wilk W=0.897624, p=0.000107886; Levene F(19,40)=0.305269, p=0.996394662.
Important correction	The newly supplied raw dataset does NOT satisfy residual normality at alpha=0.05. This corrects the earlier LDH assumption statement.
ANOVA interpretation	Two-way factorial ANOVA still detects significant material-system, condition, and interaction effects; exact values are in TwoWay_ANOVA.
Post-hoc	Tukey-adjusted simple-effects pairwise comparisons are listed in Tukey_Pairwise.

S5. Interpretation note

The supplementary statistical outputs are provided to document the analyses performed on the supplied raw datasets. Main-text interpretations should be read together with the relevant assumption diagnostics and interaction-specific post-hoc analyses presented above.
